# Synergistic mechanisms of Sanghuang–Danshen phytochemicals on postprandial vascular dysfunction in healthy subjects: A network biology approach based on a clinical trial

**DOI:** 10.1038/s41598-019-46289-3

**Published:** 2019-07-05

**Authors:** Yeni Lim, Woochang Hwang, Ji Yeon Kim, Choong Hwan Lee, Yong-Jae Kim, Doheon Lee, Oran Kwon

**Affiliations:** 1 Department of Nutritional Science and Food Management, Ewha Womans University, Seoul, 03760 Republic of Korea; 2 Department of Bio and Brain Engineering, KAIST, Daejeon, 34141 Republic of Korea; 3 Department of Food Science and Technology, Seoul National University of Science and Technology, Seoul, 01811 Republic of Korea; 4 Department of Bioscience and Biotechnology, Konkuk University, Seoul, 05029 Republic of Korea; 5 Department of Neurology, Ewha Womans University School of Medicine, Seoul, 07985 Republic of Korea

**Keywords:** Atherosclerosis, Systems analysis

## Abstract

With the increased risk of cardiovascular disease, the use of botanicals for vascular endothelial dysfunction has intensified. Here, we explored the synergistic mechanisms of Sanghuang–Danshen (SD) phytochemicals on the homeostatic protection against high-fat-induced vascular dysfunction in healthy subjects, using a network biology approach, based on a randomised crossover clinical trial. Seventeen differential markers identified in blood samples taken at 0, 3 and 6 h post-treatment, together with 12SD phytochemicals, were mapped onto the network platform, termed the context-oriented directed associations. The resulting vascular sub-networks illustrated associations between 10 phytochemicals with 32 targets implicated in 143 metabolic/signalling pathways. The three key events included adhesion molecule production (ellagic acid, fumaric acid and cryptotanshinone; VCAM-1, ICAM-1 and PLA2G2A; fatty acid metabolism), platelet activation (ellagic acid, protocatechuic acid and tanshinone IIA; VEGFA, APAF1 and ATF3; mTOR, p53, Rap1 and VEGF signalling pathways) and endothelial inflammation (all phytochemicals, except cryptotanshinone; 29 targets, including TP53 and CASP3; MAPK and PI3K-Akt signalling pathways, among others). Our collective findings demonstrate a potential of SD to protect unintended risks of vascular dysfunction in healthy subjects, providing a deeper understanding of the complicated synergistic mechanisms of signature phytochemicals in SD.

## Introduction

Under normal homeostatic conditions, the vascular endothelium plays a critical role in maintaining normal vascular tone and blood fluidity, by regulating leukocyte adhesion, platelet activity and thrombosis^1,2^. However, vascular epithelial homeostasis is continuously challenged by various life risk factors, such as obesity, smoking, dietary high-fat intake and ageing, resulting in unintended endothelial dysfunction. Vascular endothelial dysfunction is characterised by a decrease in nitric oxide availability and exaggeration of pro-inflammatory and pro-coagulation activities, which are coupled with alterations in various endothelial cell signal transductions^3–5^. These features will eventually lead to the development of cardiovascular disease if not correctly managed at an early disease stage^1,6^. As an initial preventive measure for controlling unintended vascular endothelial dysfunction, botanical phytochemicals may provide a health benefit.

Our previous *in vitro* and *in vivo* studies demonstrated that a botanical food supplement, consisting of edible mushroom Sanghuang (*Phellinus baumii*) and the rhizome of Danshen (*Salvia miltiorrhiza* Bunge) (i.e., Sanghuang–Danshen, SD), effectively suppressed the development and progression of vascular endothelial dysfunction in a rat model injected with a collagen–epinephrine mixture, to induce platelet activation^7^. This observation motivated us to assess the efficacy of SD in a clinical trial. However, a traditional clinical setting has limited applicability in realising this intention. The first limitation is from the fact that the subtle and early changes that occur in response to botanical intervention can be easily masked due to the robust homeostasis and big interpersonal differences of healthy subjects. Therefore, the differences in measured efficiency cannot be captured by measuring statistical significance between groups at P = 0.05. This issue can be resolved by combining emerging nutrigenomic technologies, specifically metabolomics, in a current clinical setting, allowing analysis of physiological processes related to homeostatic protection against unintended damages confronted in daily life^8^. The other shortcoming is that traditional clinical trials are not designed to explain how botanical phytochemicals exert synergistic actions in the human body. This limitation can be overcome by applying emerging computational network biology to a traditional clinical setting^9^. Several computational network approaches are already available to the scientific community, for exploring the potential synergistic mechanisms involved in the modulation of disease by botanical products, including an anatomical context-specific network platform, termed context-oriented directed associations (CODA), recently constructed by our research group^10–12^.

Our present investigation aimed to explore how multiple phytochemicals in SD confer homeostatic protection against endothelial dysfunction in healthy subjects, by integrating clinical data and a content-rich biological network. To this end, we conducted a randomised crossover clinical trial of SD in healthy subjects, to identify critical biochemical, molecular and metabolomic markers related to postprandial lipemia-induced epithelial dysfunction. The resulting data and signature phytochemicals of SD were then mapped onto the CODA. Furthermore, the signalling and metabolic pathways associated with SD administration were extracted from the CODA, to decipher the underlying mechanisms.

## Results

### Subject characteristics

**Figure 1:**
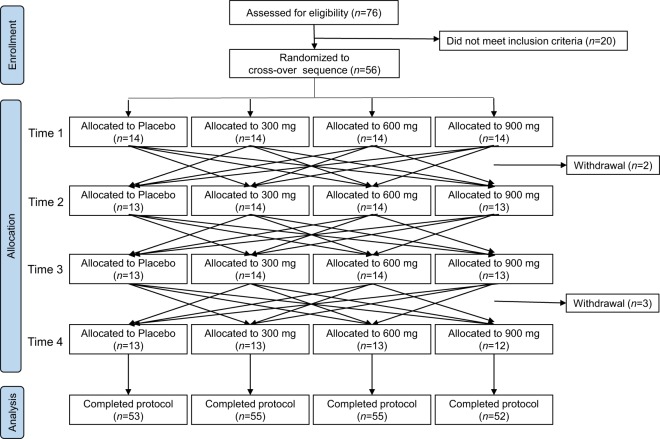
CONSORT flow diagram of the study, including enrolment of the subjects through to data analysis, as well as the primary reasons for exclusion. All subjects who completed the study were analysed.

The flow diagram of the Consolidated Standards of Reporting Trials (CONSORT) for this study is shown in Fig. 1. Fifty-six subjects were enrolled for a specific administration sequence encompassing four dose levels (0, 300, 600 and 900 mg) of SD, which contains 12 signature phytochemicals^7^. There were five withdrawals: two on the second visit, and three on the fourth visit, due to personal reasons. Therefore, 215 samples were analysed per protocol. Demographic and baseline clinical characteristics of the subjects demonstrated that the participants were healthy adults aged 31–64 years (Table 1).

**Table 1:** Demographic and baseline characteristics of the participants.

Characteristics	Value
Gender (male/female)	10/41
Average age (years)	44.5 ± 1.1
Alcohol consumption (*n*, current drinker/non-drinker)	29/22
Cigarette smoking (*n*, current smoker/non-smoker)	6/45
Total energy expenditure (kcal/day)	1694.1 ± 211.9
Waist (cm)	79.2 ± 1.0
Height (cm)	163.6 ± 1.0
Body weight (kg)	59.7 ± 1.3
Body mass index (kg/m^2^)	22.2 ± 0.4
Body temperature (°C)	36.5 ± 0.0
Systolic blood pressure (mmHg)	115.5 ± 2.0
Diastolic blood pressure (mmHg)	72.1 ± 1.5
Pulse (beats/min)	68.7 ± 1.7
Fasting blood glucose (mg/dL)	95.3 ± 1.4
Recommended food score	24.2 ± 1.0

Values are expressed as mean ± SEM.

### Identification of differential markers in the clinical setting

**Figure 2:**
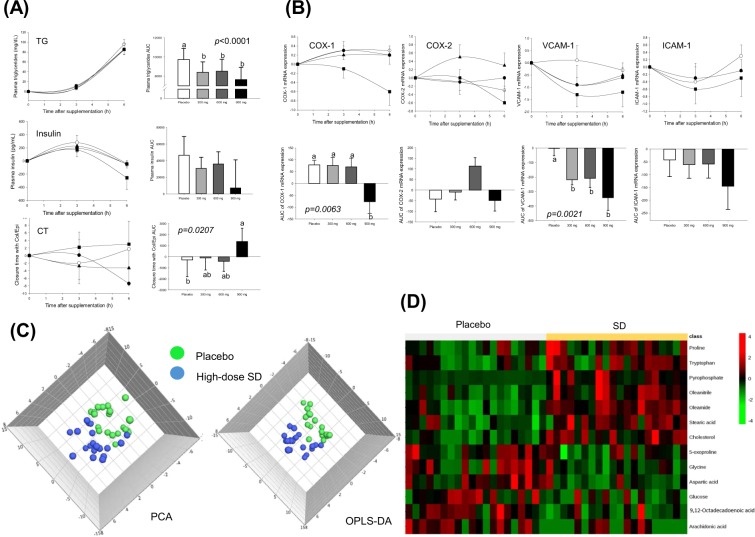
Biochemical, genetic and metabolic changes over 6 h, in blood samples taken after consumption of placebo (○) or SD (● 300, ▲ 600 and ■ 900 mg) together with a high-fat-load in healthy subjects. (**A**) Triglyceride and insulin levels in plasma, and the collagen/epinephrine-induced closure time in whole blood; (**B**) COX-1, COX-2, VCAM-1 and ICAM-1 gene expressions in PBMCs; (**C**) PCA and OPLS-DA score plots derived from GC–TOF–MS metabolites in plasma; and (**D**) heat map of differentially changed metabolites. COX, cyclooxygenase; ICAM-1, intercellular adhesion molecule-1; OPLS-DA, orthogonal partial least squares-discriminant; PBMCs, peripheral blood mononuclear cells; PCA, principal component analysis; analysis; SD, Sanghuang–Danshen; VCAM-1, vascular cell adhesion molecule-1. Values are expressed as mean ± SEM. The different letters indicate significant differences at P < 0.05.

The effects of SD on biochemical markers in blood over 6 h are shown in Fig. 2A. A high-fat intake yielded significant variations in the triglyceride (TG) and insulin levels in plasma, and the collagen/epinephrine (Col/Epi)-induced closure time (CT) in whole blood. SD consumption suppressed the extent of the TG fluctuations, resulting in a decrease in the areas under the curves (AUCs) (P < 0.0001). A similar tendency was shown in the insulin level, but this did not reach statistical significance because of the large variations within the groups. Consumption of the high-dose SD also effectively suppressed high-fat-induced platelet defects, as evidenced by the increase in the AUCs of the Col/Epi-induced CT (P = 0.0207).

Anti-platelet activation properties of SD were further analysed, by determining the changes in *cyclooxygenase* (*COX*)*-1*, *COX-2*, *intracellular adhesion molecule* (*ICAM*)*-1* and *vascular cell adhesion molecule* (*VCAM*)*-1* gene expressions in peripheral blood mononuclear cells (PBMCs) over 6 h. A high-fat load produced consistently high expression levels of the *COX-1*, *ICAM-1* and *VCAM-1* genes. In contrast, SD consumption suppressed high fat-induced *COX-1* (P = 0.0063, at a high-dose only) and *VCAM-1* (P = 0.0021) gene expressions (Fig. 2B).

Finally, the metabolic profiles were determined in the placebo and high-dose groups at 6-h, using gas chromatography–time-of-flight–mass spectrometry (GC–TOF–MS). Both principal component analysis (PCA) and orthogonal partial least squares-discriminant analysis (OPLS-DA) exhibited a significant separation of the clusters between the two groups, suggesting a considerable modification of plasma metabolites by SD administration (Fig. 2C). The heat map demonstrated the top 13 metabolites were significantly different between the two groups (Fig. 2D). The 7 metabolites (oleamide, cholesterol, oleonitrile, stearic acid, pyrophosphate, tryptophan and proline) were significantly increased, and the 6 metabolites (aspartic acid, 9,12-octadecadienoic acid, glucose, glycine, arachidonic acid and 5-oxoproline) were significantly decreased in the SD group compared with the placebo group (Supplementary Table S1).

### Identification of component-target-phenotype associations in vascular subnetwork

**Figure 3:**
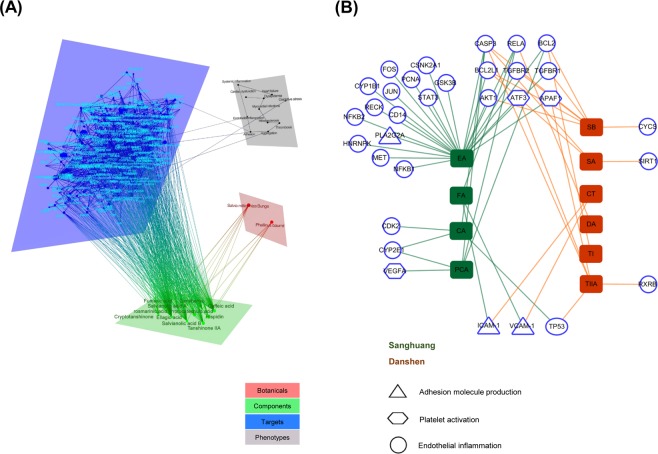
Component–target–phenotype network. (**A**) Overview of the four-layered network, and (**B**) the associations between phytochemicals and targets related to endothelial inflammation (round), platelet activation (hexagonal) and adhesion molecule production (triangle). Phytochemicals from Sanhuang (green) include CA, caffeic acid; EA, ellagic acid; FA, fumaric acid; PCA, protocatechuic acid. Those from Danshen (red) include CT, cryptotanshinone; DA, danshensu; SA, salvianolic acid A; SB, salvianolic acid B; TI, tanshinone I; TIIA, tanshinone IIA.

To understand the synergistic actions of SD phytochemicals against vascular endothelial damages from a network perspective, we first constructed a correlation network, by mapping 17 different markers obtained from the clinical setting, and 12 signature phytochemicals analysed from SD (Supplementary Fig. S1), onto the CODA. The resulting four-layered network consisted of 281 nodes and 1008 edges (Fig. 3A and Supplementary Table S2). Target proteins were further grouped by the three key functional events in adhesion molecule production (PLA2G2A/VCAM-1/ICAM-1, the triangle symbol), platelet activation (VEGFA/APAF1/ATF3, the hexagonal symbol) and endothelial inflammation (TP53/AKT1/BCL2/BCL2L1/CASP3/CD14/CDK2/CSNK2A1/CYP1B1/CYP2E1/FOS/GSK3B/HNRNPK/JUN/MET/NFKB1/NFKB2/PCNA/RECK/RELA/RXRB/SIRT1/STAT3/TGFBR1/TGFBR2, the round symbol). The resulting vascular subnetwork (Fig. 3B) identified that 10 out of 12 phytochemicals interacted directly with the above 32 target proteins (the target protein names are detailed in Supplementary Table S3).

Most of the phytochemicals, except danshensu and tanshinone I, had multiple numbers of target proteins, offering insight into their diverging results. Ellagic acid presented the broadest target scope (degree = 23), followed by salvianolic acid B (degree = 6), tanshinone IIA and protocatechuic acid (degree = 5), caffeic acid, fumaric acid and salvianolic acid A (degree = 3), and cryptotanshinone (degree = 2). Similarly, some targets were linked with more than two phytochemicals, implicating synergistic actions of phytochemicals on specific target proteins. CASP3 displayed a particularly high number of phytochemical connections (degree = 6), followed by BCL2 and RELA (degree = 4), BCL2L1 (degree = 3) and AKT1/APAF1/ATF3/CYP2E1/ICAM-1/TGFBR1/TGFBR2/TP53/VCAM-1 (degree = 2).

### Construction of the target-specific metabolic/signalling pathways associated with the vascular endothelial function

**Figure 4:**
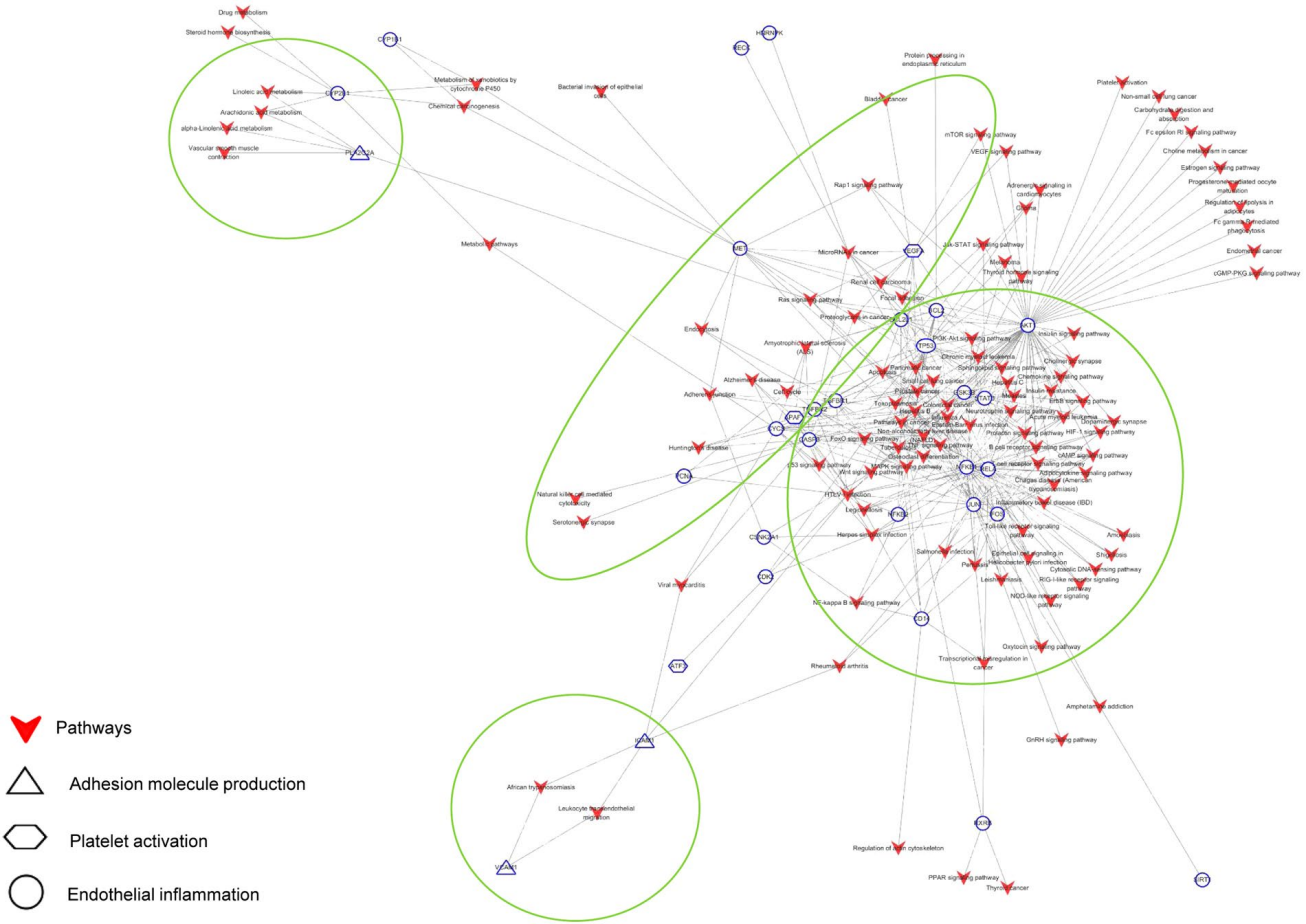
Target–pathway network. Targets related to endothelial inflammation (round), platelet activation (hexagonal), and adhesion molecule production (triangle) were linked with metabolic/signalling pathways ().

To explore the underlying mechanisms, we further analysed the metabolic/signalling pathways related to vascular endothelial dysfunction, using the CODA network platform. A total of 143 metabolic/signalling pathways, involving 32 targets and 10 SD phytochemicals were identified (Fig. 4). The functional annotation and pathway enrichment analysis revealed that α-linolenic acid metabolism was implicated in adhesion molecule production with PLA2G2A while arachidonic acid metabolism and linoleic acid metabolism were involved either in adhesion molecule production with PLA2G2A or in endothelial inflammation with CYP2E1. The mTOR, p53, PI3K-Akt, Rap1 and VEGF signalling pathways were closely related either to platelet activation with VEGFA/APAF1 or endothelial inflammation with AKT1/BCL2/CASP3/NFKB1/RELA/TP53. Of the metabolic/signalling pathways speculated to be involved in endothelial inflammation, the MAPK signalling pathway had the most number of target proteins (AKT1/CASP3/CD14/JUN/NFkB1/NFkB2/RELA/TGFBR1/TGFBR2/TP53), followed by the PI3K-Akt (AKT1/BCL2/BCL2L1/GSK3B/MET/NFkB1/RELA/TP53), Toll-like receptor (AKT1/CD14/FOS/JUN/NFkB1/RELA), TNF (AKT1/CASP3/JUN/NFkB1/RELA) and cAMP (AKT1/FOS/JUN/NFkB1/RELA) signalling pathways (Supplementary Table S4).

## Discussion

To the best of our knowledge, this is the first attempt to apply emerging analytical technologies and computational network biology to the traditional clinical setting, to overcome the current limitations in understanding how botanical phytochemicals exert homeostatic control against unintended damages confronted in daily life. As a result, we were able to provide a detailed description of the synergistic actions of SD phytochemicals against postprandial-lipaemia-induced vascular endothelial dysfunction in healthy adults.

The first hurdle facing botanical research in the traditional clinical setting of healthy adults is that subtle and early changes cannot be captured by testing the statistical significance of individual biomarkers between groups. Therefore, we employed a high-fat challenge model, to magnify the responses to botanical intervention. This strategy was based on a growing body of evidence suggesting that an extended postprandial state in daily life may result in a temporary and reversible perturbation of platelet hyperactivity in the bloodstream, along with hyperlipaemia, hyperglycaemia and hyperinsulinaemia^13–17^. We then adopted the analysis of metabolites and gene expressions, for understanding the physiological processes potentially affected by SD consumption, using a subtle and holistic approach^18–20^. Owing to the inherent sensitivity of metabolomics and gene expression, we could detect subtle alterations in biological pathways that might be useful for mining the underlying mechanisms in the following network analysis^21^. For the gene expression experiment, we decided to use PBMCs because over 80% of the gene expression in PBMCs is shared with most genes expressed in different human tissues, thereby serving as genetic “footprints” in blood^22^. Our recent animal study^7^ also confirmed that gene expression in PBMCs was remarkably compatible with that in the aorta, demonstrating that it can be applied to understand the events in the vascular endothelium.

The next hurdle encountered in a traditional clinical trial is that it is not designed to explain the synergistic actions of botanical phytochemicals. To date, preclinical studies have been carried out to investigate the synergistic effect of phytochemicals using more than two compounds individually and in combination^23^. More recently, high-throughput screening has been used in the identification of potential targets, bioactive components in botanicals and their synergistic interactions. However, these approaches are rarely successful in screening all possible cases, due to component diversity and target complexity^11^. The computational network approach is now available, providing increased opportunities to understand complex interactions between multiple phytochemicals in botanicals and multi-targets in the human body^24^. In our study, we constructed a vascular subnetwork to expand our knowledge on the synergistic effects of SD phytochemicals and fundamental biological mechanisms, by mapping the observed impact and omics data obtained from a comprehensive clinical trial to the CODA network platform.

The vascular subnetwork constructed in this study captured platelet activation, adhesion molecule production and endothelial inflammation, as key biological events related to the effects of SD against postprandial lipaemia-induced vascular dysfunction. The network demonstrated that fumaric acid in Sanghuang, together with cryptotanshinone in Danshen, were directly connected with VCAM-1 and ICAM-1, related to adhesion molecule production^25^. Meanwhile, ellagic acid (in Sanghuang) was also involved in adhesion molecule production, by regulating PLA2G2A, implicated in arachidonic acid, α-linolenic acid and linoleic acid metabolism. PLA2G2A, a member of the phospholipase A2 family, hydrolyses membrane phospholipids to fatty acids, which activate NF-kB activation and, ultimately, ICAM-1 expression^26,27^. In this way, fumaric acid, cryptotanshinone and ellagic acid would exert a synergistic influence on vascular health, by regulating adhesion molecule production, using common or separate mechanisms.

The protocatechuic acid in Sanghuang was recognised to bind to VEGFA, implicated in the mTOR, PI3K-Akt, Rap1 and VEGF signalling pathways. VEGFA is released during platelet activation, as a growth factor involved in microvascular development via its phosphorylation of downstream targets of Akt and mTOR in endothelial cells^28^. Also, VEGFA stimulation induces activation of Rap1, which regulates endothelial cell growth, migration, proliferation and tubule formation, by triggering Akt-eNOS signalling^29^. Concurrently, ellagic acid (in Sanghuang) and tanshinone IIA (in Danshen) were linked to APAF1, implicated in the p53 signalling pathway. In human platelets, APAF1, cytochrome c, and caspases-3 and -9 cooperate as an essential element of the mitochondrial death pathway^30,31^.

All phytochemicals, except cryptotanshinone in Danshen, were linked to 29 different target proteins related to endothelial inflammation. Among them, ellagic acid, in Sanghuang, was highly integrated with many different signalling pathways in endothelial inflammation, and thereby presented the broadest impact. This finding is compatible with previous studies that report protective effects of ellagic acid against oxidant-induced endothelial inflammation and atherosclerosis^32,33^. Conversely, the target interacting the greatest number of phytochemicals (ellagic acid, fumaric acid and protocatechuic acid in Sanghuang, and danshensu, salvianolic acid A, salvianolic acid B and tanshinone IIA in Danshen) was TP53, which is a transcriptional factor inducing cell cycle arrest, apoptosis or changes in metabolism in response to cellular stress^34^. Activation of TP53 is speculated to be influenced by several mechanisms, including MAPK, PI3K-Akt, sphingolipid, thyroid hormone, p53 and Wnt signalling pathways, suggesting its role as a hub. The next rank was CASP3. Ellagic acid, caffeic acid and protocatechuic acid in Sanghuang, and salvianolic acid A, salvianolic acid B and tanshinone IIA in Danshen, were connected to CASP3, mediated by the MAPK, TNF and p53 signalling pathways. CASP3 is involved in the sequential activation of caspases responsible for the execution of cell apoptosis^35^.

In summary, we demonstrated that the strategy of applying a high-fat challenge with metabolite and gene analyses to a traditional crossover clinical trial allowed detecting subtle and early effects of SD on maintaining vascular homeostasis in healthy subjects. Also, subsequent mapping of the outcomes observed in a clinical trial onto an *in silico* network model (CODA), enabled a deep understanding of the complicated synergistic mechanisms of SD phytochemicals. However, it is worth mentioning the limitations of our study. This study describes the acute outcomes after a single administration of SD. The results of another clinical trial has been recently published by our research group to report the vascular endothelial effects of 4-week SD consumption at 900 mg/day in healthy chronic smokers^36^. In addition, the contribution of this study is limited to provide a qualitative description of the relationship between the phytochemicals and targets. An extensive statistical analysis is currently under way to quantify. Even with these limitations, our approach is a novel and useful tool to overcome the inherent limitations of traditional clinical trials, for evaluating the effectiveness of botanicals in healthy subjects, although further refinement is necessary. This *in silico* model may also be used reversely, to find botanicals containing bioactive phytochemicals.

## Subjects and Methods

### Study products

The SD and a colour-matched placebo were provided by Pulmuone Foods Co., Ltd. (Seoul, Korea). Details on the preparation of SD are described in our previous publication^7^. The following 12 signature phytochemicals were quantified using a high-performance liquid chromatograph coupled with a diode array detector and MS/MS: caffeic acid, ellagic acid, fumaric acid, hispidin and protocatechuic acid from Sanghuang, and cryptotanshinone, danshensu, rosmarinic acid, salvianolic acid A, salvianolic acid B, tanshinone I and tanshinone IIA from Danshen (Supplementary Fig. S1)^7^. For standardisation purposes, protocatechuic acid (0.45–1.5 μg/g) and tanshinone IIA (50 μg/g) were chosen as marker components. The gelatine capsules were used for a double-blind challenge.

### Subjects and study design

Based on a previous study^37^, the estimated sample size of 56 subjects per group provides a statistical power of 80% to detect a difference in platelet aggregation, using a two-sided significance level of *α* = 0.05 and assuming a 20% attrition rate. The study followed a randomised, double-blinded, placebo-controlled crossover design. Subjects were recruited through poster advertisements. Eligible subjects were apparently healthy adults aged between 30 and 65 years old. Subjects were excluded if any of the following criteria were present: (1) body mass index >35 kg/m^2^; (2) a history of body weight change ≥10% in the previous 8 weeks; (3) exercising >10 h/week; (4) cigarette smoking >1 pack/day; (5) alcohol consumption >140 g/week; (6) use of medication or dietary supplements in the previous 4 weeks; (7) a history of platelet dysfunction, hypertension, stroke, diabetes and thyroid disease; (8) a history of hypersensitivity in test material; and (9) pregnancy or breastfeeding. Written informed consents were received from all participants before their participation in this study.

Fifty-six eligible subjects were enrolled in the trial and randomly assigned to receive one of the test samples in four sequences (placebo, 300, 600 or 900 mg SD), using computer-generated block randomisation (block size of four), performed by an independent researcher. Each session began with the collection of venous blood at fasting (*t* = 0) and 3 and 6 h after consuming each a high-fat-loaded test sample (total 900 kcal; 58.9% energy from fat, 33.3% energy from carbohydrate, and 7.6% energy from protein). Treatment visits were scheduled 1 week apart. During the entire trial, the subjects were advised to maintain their regular diet and lifestyle, which were monitored through a mobile phone app. The study was conducted according to the Declaration of Helsinki and approved by the Institutional Review Board of Ewha Womans University Medical Centre (EUMC 2014-04-012-014) and Ewha Womans University (79-1). The study was also registered in the World Health Organisation (WHO) International Clinical Trials Registry Platform, under the following identification: KCT0001193 (05/08/2014).

### Biochemical analysis of plasma

Plasma TG was measured by an automatic analyser (Hitachi 7600, Hitachi Co., Tokyo, Japan). Plasma insulin was determined by a human insulin enzyme-linked immunosorbent assay kit (Abcam, Cambridge, UK). CT was measured in whole blood collected in 3.8% trisodium citrate, by using a Col/Epi cartridge in a PFA-100 instrument (Siemens Healthcare Diagnostics, Marburg, Germany).

### Target gene analysis in PBMCs

Total RNA was extracted from PBMCs using TRIzol (Invitrogen, San Diego, CA, USA). The concentration and quality of the RNA were measured using a BioSpec-nano (Shimadzu Corp., Tokyo, Japan), and total RNA was reverse-transcribed using a high-capacity cDNA reverse transcription kit (Applied Biosystems, Foster City, CA, USA). The TaqMan method was used to quantify the expression of COX-1 (Ptgs1; Hs00377726_m1), COX-2 (Ptgs2; Hs00153133_m1), ICAM-1 (Icam; Hs00164932_m1), VCAM-1 (Vcam; Hs01003372_m1) and β-actin (Actb; Hs01060665_g1). The relative amounts of these mRNAs were normalised to the amount of β-actin, and the relative amounts of the RNAs were calculated using the comparative C_T_ method.

### Metabolomic analysis in plasma

Each plasma sample of the placebo and high-dose SD groups was treated with ice-cold MetOH (containing 2-chloro-l-phenylalanine as an internal standard), sonicated, left at 4 °C for 1 h and then centrifuged (13,500 *g*/4 °C/10 min). The supernatant was filtered, dried, oximated (30 °C/90 min) with methoxyamine hydrochloride in pyridine, and trimethylsilylated with *N*-methyl-*N*-trimethylsilyl-trifluoroacetamide (37 °C/30 min). The GC–TOF–MS analysis was performed by using an Agilent 7890 gas chromatograph system (Agilent Technologies, Palo Alto, CA, USA) coupled with an Agilent 7693 auto-sampler (Agilent Technologies) and equipped with a Pegasus® HT TOF–MS (LECO, St. Joseph, MI, USA) system. An Rtx-5MS column (30 m × 0.25 mm, 0.25 μm particle size; Restek Corp., Bellefonte, PA, USA) was used with a constant flow (1.5 mL/min) of helium as carrier gas. One microliter of the sample was injected into the GC. The oven temperature was initially maintained at 75 °C for 2 min and then ramped at 15 °C/min to 300 °C, and held for 3 min. The temperatures of the front inlet and transfer lines were 250 and 240 °C, respectively. The electron ionisation was carried out at −70 eV and full-scan data were acquired over a range of 50–1000 *m/z*.

### Statistical analysis

The postprandial time-series data for each subject were normalised to the respective *t* = 0 baseline value before the mean values were computed. The response of each marker was quantified as the AUC, using the trapezoidal method^38^. Data were analysed by one-way ANOVA for repeated measures, followed by Tuckey’s multiple comparison test. Data were analysed using the SAS version 9.4 (SAS Institute, Cary, NC, USA), and a significance was assumed at a two-tailed P < 0.05. The quantitative GC–TOF–MS data were subjected to multivariate statistical analysis using SIMCA-P ver. 14.1 (Umetrics, Umea, Sweden). The overall effect was visualised by the PCA and OPLS-DA, followed by variable importance in the projection analysis, to identify significant and important variables. A heat map was generated based on differential metabolites, using MetaboAnalyst 3.0 (http://metaboanalyst.ca).

### Network analysis

Two visual networks (component–target–phenotype, component–target–pathway) were constructed using the CODA. The first was designed in the following steps: (1) searching targets of signature components of SD, (2) searching related phenotypes and biological markers of vascular endothelial dysfunction, and (3) linking the components, targets and phenotypes, using the shortest paths between SD phytochemicals and markers of vascular endothelial dysfunction^39^. The biological marker–phenotype network is constructed by manual curation. The component–target–pathway was assembled in the following steps: (1) collecting all genes and metabolites that consisted of the above vascular subnetwork, (2) performing pathway enrichment analysis in a group of genes and metabolites, by Fisher’s exact test combined with a false discovery rate of <0.01, and (3) linking the component, targets, metabolites and enriched KEGG pathways http://www.genome.jp/kegg/pathway/html). Both networks were visualised using Cytoscape 3.6.1 (http://cytoscape.org/).

## Supplementary information

Supplementary information

## Data Availability

The datasets generated and analysed during the current study are available from the corresponding authors on reasonable request.
