# Supplementary material for: Synergistic mechanisms of Sanghuang–Danshen phytochemicals on postprandial vascular dysfunction in healthy subjects: A network biology approach based on a clinical trial
Source: Sci Rep. 2019 Jul 5;9:9746. doi: 10.1038/s41598-019-46289-3 (PMC6611899; doi:10.1038/s41598-019-46289-3)
Supplement: Supplementary file 1 — Supplementary information [file 41598_2019_46289_MOESM1_ESM.pdf]

Synergistic mechanisms of Sanghuang–Danshen phytochemicals on postprandial vascular dysfunction in healthy subjects: A network biology approach based on a clinical trial

Yeni Lim[1§], Woochang Hwang[2§], Ji Yeon Kim[3], Choong Hwan Lee[4], Yong-Jae Kim[5], Doheon Lee[2]* & Oran Kwon[1]*

[1]Department of Nutritional Science and Food Management, Ewha Womans University, Seoul 03760, Republic of Korea. [2]Department of Bio and Brain Engineering, KAIST, Daejeon 34141, Republic of Korea. [3]Department of Food Science and Technology, Seoul National University of Science and Technology, Seoul 01811, Republic of Korea. [4]Department of Bioscience and Biotechnology, Konkuk University, Seoul 05029, Republic of Korea. [5]Department of Neurology, Ewha Womans University School of Medicine, Seoul 07985, Republic of Korea.

[§]**Co-first authors.**

ynlim@ewha.ac.kr (Y.L); wchwang.kr@gmail.com (W.H)

***Co-corresponding authors.**

orank@ewha.ac.kr (O.K.); dhlee@kaist.ac.kr (D.L)

(A)

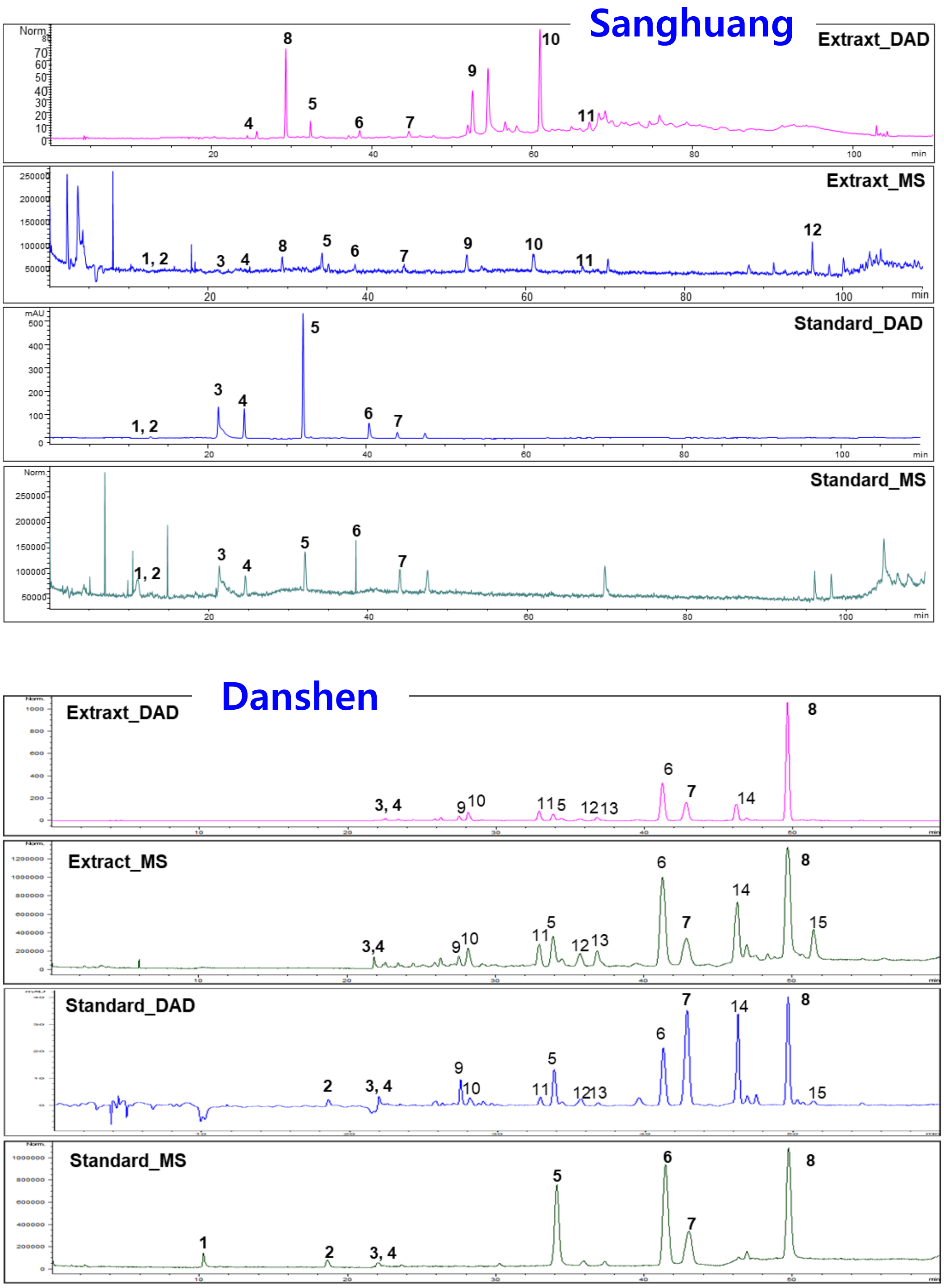

<Sanghuang> 1, succinic acid; 2, fumaric acid; 3, 5-hydroxymethyl-2-furaldehyde; 4, protocatechuic acid; 5, caffeic acid; 6, ellagic acid; 7, hispidin; 8, protocatechuicaldehyde; 9, davallialactone; 10, hypholomine B; 11, inoscavin A; 12, phellinsin A

<Danshen> 1, danshensu sodium salt; 2, rosmarinic acid; 3, salvianolic acid B; 4, salvianolic acid A; 5, 15,16-dihydrotanshinone I; 6, tanshinone I; 7, cryptotanshinone; 8, tanshinone IIA; 9, 1,2-didehydrotanshinone IIA; 10, tanshinone IIB; 11, 1-ketoisocryptotanshinone; 12, 1, 2-didehydromiltirone; 13, methyl tanshinate; 14, 1,2-dihydrotanshinone I; and 15, miltirone

(B)

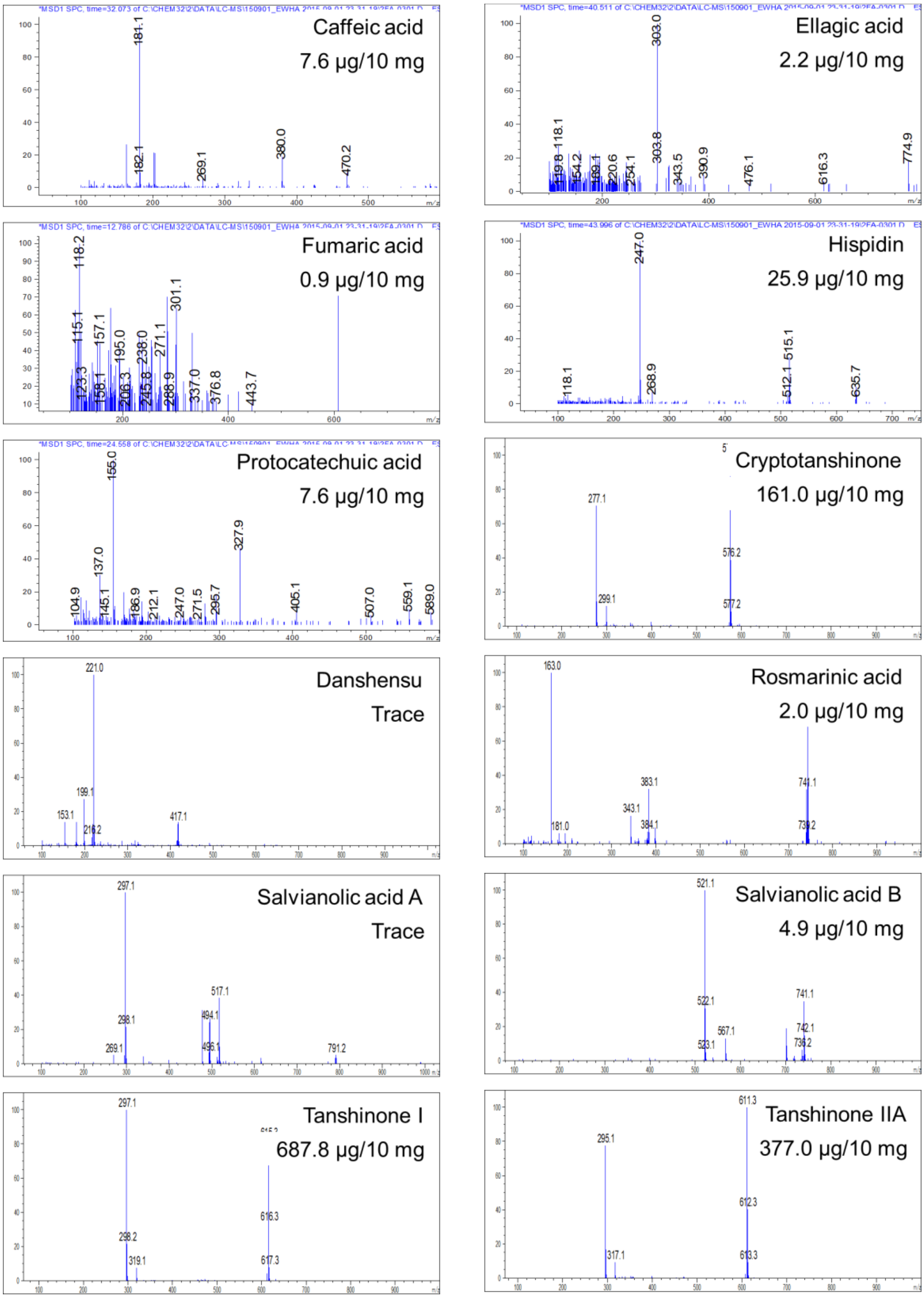

Supplementary Figure S1. Phytochemical profiling of Sanghuang-Danshen. (A) Total Ion Chromatogram of Sanghuang and Danshen. (B) Mass spectrum (positive mode) of 12 signature phytochemicals quantified.

Supplementary Table S1. Significantly altered metabolites and their metabolic pathways

Metabolite	*P*-value[1]	Related pathway
Arachidonic acid	0.0180	Arachidonic acid metabolism
Aspartic acid	0.0030	Aminoacyl-tRNA biosynthesis; Nitrogen metabolism; Glycine, serine and threonine metabolism; Cyanoamino acid metabolism; Cysteine and methionine metabolism; Arginine and proline metabolism; Alanine, aspartate and glutamate metabolism
Cholesterol	<0.0001	Primary bile acid biosynthesis
Glucose	0.0147	Glycolysis or Gluconeogenesis
Glycine	0.0139	Aminoacyl-tRNA biosynthesis; Nitrogen metabolism; Glycine, serine and threonine metabolism; Cyanoamino acid metabolism; Glutathione metabolism; Primary bile acid biosynthesis
Oleamide	<0.0001	-
Oleanitrile	<0.0001	-
Proline	0.0156	Aminoacyl-tRNA biosynthesis; Arginine and proline metabolism
Pyrophosphate	0.0013	-
Stearic acid	0.0002	Fatty acid biosynthesis
Tryptophan	0.0015	Aminoacyl-tRNA biosynthesis; Nitrogen metabolism; Glycine, serine and threonine metabolism
5-oxoproline	0.0363	Glutathione metabolism
9,12-octadecadienoic acid	0.0085	Linoleic acid metabolism

[1] *P*-values derived from the student's *t*-test between groups, $P < 0.05$.

Supplementary Table S2. The list of component-target-phenotype

Phellinus baumii	Fumaric acid	Herb	Compound
Phellinus baumii	protocatechuic acid	Herb	Compound
Phellinus baumii	Caffeic acid	Herb	Compound
Phellinus baumii	Ellagic acid	Herb	Compound
Phellinus baumii	Hispidin	Herb	Compound
Salvia miltiorrhiza Bunge	Tanshinone I	Herb	Compound
Salvia miltiorrhiza Bunge	Tanshinone IIA	Herb	Compound
Salvia miltiorrhiza Bunge	Cryptotanshinone	Herb	Compound
Salvia miltiorrhiza Bunge	salvianolic acid B	Herb	Compound
Salvia miltiorrhiza Bunge	danshensu	Herb	Compound
Salvia miltiorrhiza Bunge	rosmarinic acid	Herb	Compound
Salvia miltiorrhiza Bunge	Salvianolic acid A	Herb	Compound
salvianolic acid B	NOS2	Compound	Molecule
Ellagic acid	AKT1	Compound	Molecule
salvianolic acid B	AKT1	Compound	Molecule
Ellagic acid	APAF1	Compound	Molecule
Tanshinone IIA	APAF1	Compound	Molecule
Ellagic acid	IL1R1	Compound	Molecule
Tanshinone I	TNF	Compound	Molecule
Ellagic acid	IGF1R	Compound	Molecule
protocatechuic acid	CDKN1A	Compound	Molecule
Salvianolic acid A	TNF	Compound	Molecule
Ellagic acid	CDKN1A	Compound	Molecule
Ellagic acid	ATF3	Compound	Molecule
Tanshinone IIA	ATF3	Compound	Molecule
salvianolic acid B	NCF1	Compound	Molecule
protocatechuic acid	NFKBIB	Compound	Molecule
Ellagic acid	BCL2	Compound	Molecule
Hispidin	SMAD2	Compound	Molecule
protocatechuic acid	BCL2	Compound	Molecule
protocatechuic acid	NOS2	Compound	Molecule
Caffeic acid	CCL4	Compound	Molecule
Ellagic acid	SYK	Compound	Molecule
protocatechuic acid	IL1B	Compound	Molecule
salvianolic acid B	BCL2	Compound	Molecule
Tanshinone I	BCL2	Compound	Molecule
Caffeic acid	UGT1A10	Compound	Molecule
Caffeic acid	TP53	Compound	Molecule
protocatechuic acid	CYP1A2	Compound	Molecule
Ellagic acid	BCL2L1	Compound	Molecule
Ellagic acid	IL1B	Compound	Molecule
Ellagic acid	INSR	Compound	Molecule
Salvianolic acid A	BCL2L1	Compound	Molecule
salvianolic acid B	TLR4	Compound	Molecule
Fumaric acid	IFNG	Compound	Molecule
Ellagic acid	TNF	Compound	Molecule
salvianolic acid B	BCL2L1	Compound	Molecule
Caffeic acid	CASP3	Compound	Molecule

Caffeic acid	GSTP1	Compound	Molecule
Caffeic acid	CXCL2	Compound	Molecule
Ellagic acid	TGFB1	Compound	Molecule
rosmarinic acid	TNF	Compound	Molecule
Ellagic acid	ERBB2	Compound	Molecule
Ellagic acid	CASP3	Compound	Molecule
Tanshinone I	CYP1A2	Compound	Molecule
Fumaric acid	TNF	Compound	Molecule
protocatechuic acid	CASP3	Compound	Molecule
Salvianolic acid A	CASP3	Compound	Molecule
Tanshinone IIA	CCND1	Compound	Molecule
Tanshinone IIA	RXRA	Compound	Molecule
salvianolic acid B	CASP3	Compound	Molecule
Ellagic acid	CALR	Compound	Molecule
Tanshinone IIA	CASP3	Compound	Molecule
Caffeic acid	PTGS2	Compound	Molecule
Ellagic acid	CD14	Compound	Molecule
Caffeic acid	GSTM1	Compound	Molecule
protocatechuic acid	PTGS2	Compound	Molecule
protocatechuic acid	CYP1A1	Compound	Molecule
Ellagic acid	CCND1	Compound	Molecule
Ellagic acid	MMP9	Compound	Molecule
Ellagic acid	CSNK2A1	Compound	Molecule
Tanshinone IIA	CXCL8	Compound	Molecule
Caffeic acid	UGT1A3	Compound	Molecule
Ellagic acid	CYP1B1	Compound	Molecule
Ellagic acid	HRAS	Compound	Molecule
Tanshinone IIA	TNF	Compound	Molecule
Caffeic acid	PIK3R1	Compound	Molecule
salvianolic acid B	IL1B	Compound	Molecule
Caffeic acid	CYP2E1	Compound	Molecule
Ellagic acid	IRF9	Compound	Molecule
protocatechuic acid	CYP2E1	Compound	Molecule
Ellagic acid	FOS	Compound	Molecule
Ellagic acid	GSK3B	Compound	Molecule
Ellagic acid	HNRNPK	Compound	Molecule
Ellagic acid	TEK	Compound	Molecule
protocatechuic acid	TNF	Compound	Molecule
Hispidin	PRKCB	Compound	Molecule
Ellagic acid	EGFR	Compound	Molecule
Tanshinone I	NR1I2	Compound	Molecule
Cryptotanshinone	ICAM1	Compound	Molecule
Ellagic acid	CXCL8	Compound	Molecule
Caffeic acid	ALOX15	Compound	Molecule
Caffeic acid	UGT1A8	Compound	Molecule
salvianolic acid B	TGFB1	Compound	Molecule
Fumaric acid	ICAM1	Compound	Molecule
Caffeic acid	CXCL8	Compound	Molecule
Ellagic acid	JUN	Compound	Molecule
Salvianolic acid A	NOS2	Compound	Molecule

Ellagic acid	MET	Compound	Molecule
Cryptotanshinone	CYP1A2	Compound	Molecule
Ellagic acid	SRC	Compound	Molecule
Ellagic acid	NFKB1	Compound	Molecule
Ellagic acid	PTK2	Compound	Molecule
Ellagic acid	ZAP70	Compound	Molecule
Ellagic acid	CYP1A1	Compound	Molecule
Ellagic acid	NFKB2	Compound	Molecule
Ellagic acid	PCNA	Compound	Molecule
Fumaric acid	NFKBIA	Compound	Molecule
Tanshinone I	PARP1	Compound	Molecule
Ellagic acid	PLA2G2A	Compound	Molecule
Ellagic acid	RECK	Compound	Molecule
Ellagic acid	GPX1	Compound	Molecule
Ellagic acid	PTGS2	Compound	Molecule
danshensu	RELA	Compound	Molecule
Ellagic acid	RELA	Compound	Molecule
Ellagic acid	MAP3K8	Compound	Molecule
salvianolic acid B	CYCS	Compound	Molecule
Ellagic acid	HSPA1A	Compound	Molecule
Caffeic acid	TNF	Compound	Molecule
Caffeic acid	CDK2	Compound	Molecule
Ellagic acid	GPX5	Compound	Molecule
Caffeic acid	UGT1A7	Compound	Molecule
Salvianolic acid A	IFNG	Compound	Molecule
Fumaric acid	RELA	Compound	Molecule
Ellagic acid	GTF2E2	Compound	Molecule
salvianolic acid B	TNF	Compound	Molecule
Tanshinone I	CYP3A4	Compound	Molecule
Cryptotanshinone	CYP3A4	Compound	Molecule
protocatechuic acid	RELA	Compound	Molecule
Tanshinone IIA	RXRB	Compound	Molecule
Ellagic acid	KDR	Compound	Molecule
Caffeic acid	ALOX5	Compound	Molecule
Cryptotanshinone	CYP1A1	Compound	Molecule
protocatechuic acid	SYK	Compound	Molecule
Ellagic acid	HMOX1	Compound	Molecule
Salvianolic acid A	SIRT1	Compound	Molecule
Ellagic acid	MAPK11	Compound	Molecule
Ellagic acid	STAT3	Compound	Molecule
Source	Target	Source attribute	Target attribute
Ellagic acid	TGFBR1	Compound	Molecule
Ellagic acid	GSTA4	Compound	Molecule
Tanshinone I	CYP1A1	Compound	Molecule
Salvianolic acid A	SHC1	Compound	Molecule
salvianolic acid B	TGFBR1	Compound	Molecule
Ellagic acid	TGFBR2	Compound	Molecule
Ellagic acid	PDGFRB	Compound	Molecule
Ellagic acid	PRKCE	Compound	Molecule
Salvianolic acid A	IL1B	Compound	Molecule

salvianolic acid B	TGFBR2	Compound	Molecule
Ellagic acid	FGR	Compound	Molecule
Tanshinone IIA	TP53	Compound	Molecule
Ellagic acid	MAP3K1	Compound	Molecule
Ellagic acid	EP300	Compound	Molecule
Ellagic acid	SMAD3	Compound	Molecule
Caffeic acid	GSTM2	Compound	Molecule
Cryptotanshinone	VCAM1	Compound	Molecule
Fumaric acid	VCAM1	Compound	Molecule
protocatechuic acid	VEGFA	Compound	Molecule
HSPA1A	PYGB	Molecule	Molecule
BCL2	GCLC	Molecule	Molecule
STAT1	SOAT1	Molecule	Molecule
CXCL8	MAPK10	Molecule	Molecule
TLR4	SULT2B1	Molecule	Molecule
ERBB2	PIK3CB	Molecule	Molecule
MTF1	GCLC	Molecule	Molecule
GTF2E2	SND1	Molecule	Molecule
EP300	SHMT1	Molecule	Molecule
OPLAH	UBC	Molecule	Molecule
SMAD2	EP300	Molecule	Molecule
LIPA	UBC	Molecule	Molecule
IFNG	SHMT1	Molecule	Molecule
NOS2	citrulline	Molecule	Molecule
PLA2G4A	arachidonic acid	Molecule	Molecule
JMJD7-PLA2G4B	linoleic acid	Molecule	Molecule
NFATC2	PRODH	Molecule	Molecule
EP300	PLA2G2A	Molecule	Molecule
PTGS2	GAD1	Molecule	Molecule
AKT1	JUN	Molecule	Molecule
JUN	PTGS2	Molecule	Molecule
NFKBIA	GCLC	Molecule	Molecule
GLS	glutamic acid	Molecule	Molecule
EP300	PYGB	Molecule	Molecule
PARP1	TP53	Molecule	Molecule
TGFBR1	ALDH2	Molecule	Molecule
GPX1	DHCR7	Molecule	Molecule
PTK2	PYGL	Molecule	Molecule
CYP1A1	PYGL	Molecule	Molecule
NCL	PTGS1	Molecule	Molecule
TNF	SHH	Molecule	Molecule
SRC	GSTP1	Molecule	Molecule
GATM	glycine	Molecule	Molecule
RXRB	GSTP1	Molecule	Molecule
PLA2G16	arachidonic acid	Molecule	Molecule
HRAS	SHH	Molecule	Molecule
SHH	cholesterol	Molecule	Molecule
SRC	ANPEP	Molecule	Molecule
RXRA	HNF4A	Molecule	Molecule
VEGFA	PTGS2	Molecule	Molecule

SYK	FCGR2A	Molecule	Molecule
3H-proline	PYCR2	Molecule	Molecule
ICAM1	TP53	Molecule	Molecule
IL1B	PLA2G2A	Molecule	Molecule
TNF	PYGB	Molecule	Molecule
RXRB	CYP3A4	Molecule	Molecule
GSTA4	C14786	Molecule	Molecule
ALOX5	arachidonic acid	Molecule	Molecule
HNF1B	SI	Molecule	Molecule
ALOX5	PTPA	Molecule	Molecule
IGF1R	MAPK3	Molecule	Molecule
TGFBR2	SNAI1	Molecule	Molecule
CALR	SULT2B1	Molecule	Molecule
PRKCD	PLA2G4B	Molecule	Molecule
NOS2	SHMT1	Molecule	Molecule
IRF1	PYGB	Molecule	Molecule
GSTP1	SULT2B1	Molecule	Molecule
CD14	ALDH2	Molecule	Molecule
CYP2E1	linoleic acid	Molecule	Molecule
L-delta 1-pyrroline-5-carboxylate	3H-proline	Molecule	Molecule
SHC1	CYP2E1	Molecule	Molecule
CDK2	FOXO3	Molecule	Molecule
SYK	PIK3CG	Molecule	Molecule
oxaloacetate	aspartic acid	Molecule	Molecule
PIK3R1	EGFR	Molecule	Molecule
NFKBIB	AKT1	Molecule	Molecule
SNAI1	PTPA	Molecule	Molecule
NFKB1	CYP17A1	Molecule	Molecule
RELA	PTGS2	Molecule	Molecule
glucose	GLA	Molecule	Molecule
UGT1A10	estradiol	Molecule	Molecule
PCK2	oxaloacetate	Molecule	Molecule
HIF1A	PTGS2	Molecule	Molecule
TEK	CRK	Molecule	Molecule
SND1	MTF1	Molecule	Molecule
VEGFA	GAD2	Molecule	Molecule
RXRB	SHMT1	Molecule	Molecule
CYP1B1	PPP1CC	Molecule	Molecule
ICAM1	SULT2B1	Molecule	Molecule
CCND1	GATM	Molecule	Molecule
HSPA1A	SULT2B1	Molecule	Molecule
IFNG	IL6R	Molecule	Molecule
NFKBIB	RELA	Molecule	Molecule
FCGR3A	PRKCB	Molecule	Molecule
SRC	PRKCB	Molecule	Molecule
TP53	PRODH	Molecule	Molecule
UBC	ICAM1	Molecule	Molecule
GSK3B	CTNNB1	Molecule	Molecule
RXRB	PTPA	Molecule	Molecule

CCND1	PLA2G2A	Molecule	Molecule
PLCB1	PRODH	Molecule	Molecule
HNF1A	SI	Molecule	Molecule
CDKN1A	TP53	Molecule	Molecule
FOS	PRODH	Molecule	Molecule
ERBB2	GSS	Molecule	Molecule
CYP1A2	linoleic acid	Molecule	Molecule
ICAM1	PYGB	Molecule	Molecule
GSTM2	reduced glutathione	Molecule	Molecule
TGFB1	MAPK10	Molecule	Molecule
IGF1R	IGF1	Molecule	Molecule
PTK2	GSS	Molecule	Molecule
UBC	VCAM1	Molecule	Molecule
CXCL2	PYGB	Molecule	Molecule
glucose	GANC	Molecule	Molecule
SHC1	PTPRF	Molecule	Molecule
TLR4	GATM	Molecule	Molecule
CYP1A2	DHEA	Molecule	Molecule
DLG1	DAO	Molecule	Molecule
ANPEP	glycine	Molecule	Molecule
GRIN2B	PRODH	Molecule	Molecule
KDR	PRODH	Molecule	Molecule
ICAM1	PLA2G2A	Molecule	Molecule
DHEA	SULT2B1	Molecule	Molecule
HMOX1	GATM	Molecule	Molecule
KDR	PLA2G2A	Molecule	Molecule
TGFBR1	TP53	Molecule	Molecule
linoleic acid	PLA2G1B	Molecule	Molecule
GSTM1	C14786	Molecule	Molecule
GLA	FBXO6	Molecule	Molecule
chloroform/methanol	CYP2E1	Molecule	Molecule
PPARA	PTGS2	Molecule	Molecule
NR1I2	GSTP1	Molecule	Molecule
PIK3R1	GSS	Molecule	Molecule
SMAD3	TGFB1	Molecule	Molecule
HRAS	GATM	Molecule	Molecule
CYP1B1	GCLC	Molecule	Molecule
GANC	UBC	Molecule	Molecule
INSR	PIK3CA	Molecule	Molecule
DLG1	GAD1	Molecule	Molecule
CYP1B1	TP53	Molecule	Molecule
SMAD2	TGFB1	Molecule	Molecule
CYCS	CYP2E1	Molecule	Molecule
MAPK9	TP53	Molecule	Molecule
PLA2G2A	TP53	Molecule	Molecule
IRS1	SHH	Molecule	Molecule
RXRB	PTGS2	Molecule	Molecule
NOS2	IL1B	Molecule	Molecule
FOXO3	G6PC2	Molecule	Molecule
GSTM1	PYGL	Molecule	Molecule

TGFB1	SULT2B1	Molecule	Molecule
EP300	SULT2B1	Molecule	Molecule
IL1B	GATM	Molecule	Molecule
RXRA	PPARA	Molecule	Molecule
UGT1A3	NR3C1	Molecule	Molecule
PTPRF	PTPA	Molecule	Molecule
UGT1A8	androsterone	Molecule	Molecule
CRK	GATM	Molecule	Molecule
SIRT1	GAD2	Molecule	Molecule
FOXO4	G6PC3	Molecule	Molecule
CDK2	FOXO4	Molecule	Molecule
MET	GAD1	Molecule	Molecule
FOXO6	PCK2	Molecule	Molecule
MYC	CAD	Molecule	Molecule
UGT1A7	androsterone	Molecule	Molecule
PIK3R1	GNAI2	Molecule	Molecule
SI	glucose	Molecule	Molecule
5(S-HPETE	arachidonic acid	Molecule	Molecule
PCNA	CYP2E1	Molecule	Molecule
SYK	PIK3CA	Molecule	Molecule
SMAD3	JUN	Molecule	Molecule
PTK2	GNMT	Molecule	Molecule
HRAS	JUN	Molecule	Molecule
IFNG	CYP27A1	Molecule	Molecule
SMAD3	EP300	Molecule	Molecule
ATF3	PTGS2	Molecule	Molecule
ALDH7A1	beta-alanine	Molecule	Molecule
BCL2L1	GCLC	Molecule	Molecule
CSNK2A1	NFKBIA	Molecule	Molecule
PIK3R1	GATM	Molecule	Molecule
MIR122	CYP7A1	Molecule	Molecule
MAP3K8	GCLC	Molecule	Molecule
RELA	MAPK3	Molecule	Molecule
PLA2G2A	linoleic acid	Molecule	Molecule
arachidonic acid	PTGS2	Molecule	Molecule
TEK	GLUL	Molecule	Molecule
DLG1	PRODH	Molecule	Molecule
PTGS2	PYGL	Molecule	Molecule
MAPK11	JUN	Molecule	Molecule
STAT3	GATM	Molecule	Molecule
PLA2G4B	linoleic acid	Molecule	Molecule
CXCL2	TP53	Molecule	Molecule
HSPA1A	TP53	Molecule	Molecule
PRKCE	GCLC	Molecule	Molecule
TGFBR1	SNAI1	Molecule	Molecule
CXCL2	GATM	Molecule	Molecule
CASP3	PRODH	Molecule	Molecule
STAT3	MYC	Molecule	Molecule
linoleic acid	PLA2G2A	Molecule	Molecule
PYCR2	VCAM1	Molecule	Molecule

IL1R1	NFKB1	Molecule	Molecule
CYP46A1	cholesterol	Molecule	Molecule
TEK	IRS1	Molecule	Molecule
CYCS	GPX1	Molecule	Molecule
MAP3K1	SHMT1	Molecule	Molecule
CYP2J2	linoleic acid	Molecule	Molecule
TBP	ALAS2	Molecule	Molecule
CYP3A4	SULT2B1	Molecule	Molecule
CYCS	PCK1	Molecule	Molecule
CCND1	GCLC	Molecule	Molecule
AKT1	PPP1R3A	Molecule	Molecule
MAPK10	TP53	Molecule	Molecule
FOS	CYP2J2	Molecule	Molecule
HNRNPK	CYP17A1	Molecule	Molecule
CSNK2A1	CYP1A2	Molecule	Molecule
CD14	TNF	Molecule	Molecule
CCND1	PYGB	Molecule	Molecule
TP53	CYP2E1	Molecule	Molecule
STAT3	PTPA	Molecule	Molecule
ERBB2	MAPK3	Molecule	Molecule
CDKN1A	MAPK3	Molecule	Molecule
CYP2E1	SHMT1	Molecule	Molecule
NFKB1	PTGS2	Molecule	Molecule
CYP2E1	GOT1	Molecule	Molecule
PTGS2	GSS	Molecule	Molecule
HMOX1	CYP46A1	Molecule	Molecule
SOAT1	cholesterol	Molecule	Molecule
HCFC1	GNMT	Molecule	Molecule
PLA2G4A	linoleic acid	Molecule	Molecule
ERBB2	SULT2B1	Molecule	Molecule
CALR	PYGB	Molecule	Molecule
VCAM1	PIK3CB	Molecule	Molecule
glutamic acid	aspartic acid	Molecule	Molecule
MMP9	GLUL	Molecule	Molecule
UGT1A3	PRODH	Molecule	Molecule
RELA	SHMT1	Molecule	Molecule
GSK3B	SULT2B1	Molecule	Molecule
TGFBR1	TGFBR2	Molecule	Molecule
MAPK3	PLA2G2A	Molecule	Molecule
ALOX5	ALDH7A1	Molecule	Molecule
IL1R1	GSTM1	Molecule	Molecule
CASP8	PYGL	Molecule	Molecule
SRC	PIK3CB	Molecule	Molecule
CCL4	TP53	Molecule	Molecule
ALDH2	beta-alanine	Molecule	Molecule
RELA	TP53	Molecule	Molecule
SIRT1	PTGS2	Molecule	Molecule
STAT1	PCK1	Molecule	Molecule
C14786	CYP2E1	Molecule	Molecule
CASP9	MYC	Molecule	Molecule

GSS	glycine	Molecule	Molecule
EGFR	PYGL	Molecule	Molecule
G6PC3	glucose	Molecule	Molecule
GSK3B	CYP2E1	Molecule	Molecule
SHMT1	glycine	Molecule	Molecule
IL1B	GOT1	Molecule	Molecule
HNRNPK	CYP3A4	Molecule	Molecule
INSR	CYP46A1	Molecule	Molecule
IGF1R	PYGL	Molecule	Molecule
TGFBR1	GSTM1	Molecule	Molecule
CYP1B1	SULT2B1	Molecule	Molecule
UBC	NCL	Molecule	Molecule
BCL2	PTPA	Molecule	Molecule
GSTM2	GOT1	Molecule	Molecule
JUND	GCLC	Molecule	Molecule
AKT1	PTGS2	Molecule	Molecule
UBC	NOS3	Molecule	Molecule
MAP3K8	PYGB	Molecule	Molecule
MAPK11	HRAS	Molecule	Molecule
VEGFA	SULT2B1	Molecule	Molecule
GSTA4	TNF	Molecule	Molecule
GSTP1	PYGB	Molecule	Molecule
reduced glutathione	GSTM1	Molecule	Molecule
CDK2	FOXO6	Molecule	Molecule
STAT6	ALOX15	Molecule	Molecule
GSTM1	SULT2B1	Molecule	Molecule
MAPK11	FOXO6	Molecule	Molecule
FOS	SHH	Molecule	Molecule
GSK3B	GCLC	Molecule	Molecule
PPP1CC	PYGM	Molecule	Molecule
RXRB	PPARG	Molecule	Molecule
NOS2	MAPK1	Molecule	Molecule
APAF1	SHH	Molecule	Molecule
AKT1	SULT2B1	Molecule	Molecule
MET	CYP2E1	Molecule	Molecule
GPX1	PCK1	Molecule	Molecule
arachidonic acid	PTGS1	Molecule	Molecule
TEK	PLCB1	Molecule	Molecule
CXCL2	GCLC	Molecule	Molecule
CSNK2A1	PTGS2	Molecule	Molecule
linoleic acid	PLA2G4F	Molecule	Molecule
NFKB1	AKT1	Molecule	Molecule
CYP2E1	UBC	Molecule	Molecule
IRF9	STAT3	Molecule	Molecule
FOS	SHMT1	Molecule	Molecule
JUN	TP53	Molecule	Molecule
PRODH	3H-proline	Molecule	Molecule
RXRA	PPARG	Molecule	Molecule
CDKN1A	GGT1	Molecule	Molecule
RELA	GCLC	Molecule	Molecule

GSTA4	reduced glutathione	Molecule	Molecule
PYGM	glucose	Molecule	Molecule
KDR	CYP17A1	Molecule	Molecule
GPX5	15(S-HPETE	Molecule	Molecule
VCAM1	PYGB	Molecule	Molecule
APAF1	HNF1A	Molecule	Molecule
HRAS	MAPK3	Molecule	Molecule
CYP3A4	SHMT1	Molecule	Molecule
NCF1	STS	Molecule	Molecule
MAPK14	GCLC	Molecule	Molecule
HMOX1	PLA2G2A	Molecule	Molecule
CD14	PTGS2	Molecule	Molecule
PIK3R1	HIF1A	Molecule	Molecule
NFKBIA	SHH	Molecule	Molecule
FOS	PYGL	Molecule	Molecule
PRKCB	arachidonic acid	Molecule	Molecule
CXCL8	MAPK3	Molecule	Molecule
calcium ions	NOS3	Molecule	Molecule
IGF1R	SULT2B1	Molecule	Molecule
SND1	APOE	Molecule	Molecule
HMOX1	PYGB	Molecule	Molecule
CXCL8	SULT2B1	Molecule	Molecule
CYP1A2	GCK	Molecule	Molecule
IFNG	MAPK1	Molecule	Molecule
reduced glutathione	GSTP1	Molecule	Molecule
TNF	GATM	Molecule	Molecule
BCL2L1	PTGS2	Molecule	Molecule
MET	SHH	Molecule	Molecule
CYCS	PTGS2	Molecule	Molecule
GSTP1	GOT1	Molecule	Molecule
BCL2L1	TP53	Molecule	Molecule
ALDH1B1	beta-alanine	Molecule	Molecule
SYK	FCGR1A	Molecule	Molecule
FCGR2A	STS	Molecule	Molecule
STAT3	CYP2E1	Molecule	Molecule
PCNA	PTGS2	Molecule	Molecule
SULT2B1	cholesterol	Molecule	Molecule
MAPK3	PTPA	Molecule	Molecule
CYP1A2	SHMT1	Molecule	Molecule
PPP1R3A	PYGL	Molecule	Molecule
RXRB	CYP17A1	Molecule	Molecule
NFKBIA	PLA2G2A	Molecule	Molecule
GSK3B	GATM	Molecule	Molecule
AKT1	CYP2E1	Molecule	Molecule
RECK	SHMT2	Molecule	Molecule
DAO	glycine	Molecule	Molecule
AKT1	SHMT1	Molecule	Molecule
PPARG	PRODH	Molecule	Molecule
KDR	GATM	Molecule	Molecule
BCL2	SULT2B1	Molecule	Molecule

MAP3K8	PLA2G2A	Molecule	Molecule
PIK3CA	PCK1	Molecule	Molecule
GAD1	glutamic acid	Molecule	Molecule
VCAM1	PLA2G2A	Molecule	Molecule
ALOX15	GCLC	Molecule	Molecule
ASS1	FBXO6	Molecule	Molecule
pyroglutamate	GGCT	Molecule	Molecule
UGT1A3	chloroform/methanol	Molecule	Molecule
EGFR	PCK1	Molecule	Molecule
PRKCB	PTPA	Molecule	Molecule
APOE	PRODH	Molecule	Molecule
BCL2L1	SHH	Molecule	Molecule
RXRA	GGT1	Molecule	Molecule
CYP2E1	GCLC	Molecule	Molecule
CYP3A4	TP53	Molecule	Molecule
PIK3CG	GAD1	Molecule	Molecule
NR3C1	CYP27A1	Molecule	Molecule
CALR	TP53	Molecule	Molecule
NCF1	ALOX15	Molecule	Molecule
CYP3A4	HNF1B	Molecule	Molecule
NR1I2	CYP17A1	Molecule	Molecule
CASP3	CYP46A1	Molecule	Molecule
ZAP70	DLG1	Molecule	Molecule
PRKCB	DAO	Molecule	Molecule
RXRA	CYP2E1	Molecule	Molecule
BCL2	PTGS2	Molecule	Molecule
POLD1	SULT2B1	Molecule	Molecule
CDKN1A	GATM	Molecule	Molecule
CYCS	GLUL	Molecule	Molecule
CDK2	TP53	Molecule	Molecule
SHC1	SRC	Molecule	Molecule
PTGS2	PRODH	Molecule	Molecule
PRKCB	DHCR7	Molecule	Molecule
CYP1B1	PTGS2	Molecule	Molecule
PCNA	GSTP1	Molecule	Molecule
PDGFRB	PIK3CB	Molecule	Molecule
CSNK2A1	TP53	Molecule	Molecule
PDGFRB	PIK3CG	Molecule	Molecule
TNF	CYP2E1	Molecule	Molecule
gamma-glutamylcysteine	pyroglutamate	Molecule	Molecule
BCL2L1	GATM	Molecule	Molecule
FGR	PIK3CA	Molecule	Molecule
CYP7A1	cholesterol	Molecule	Molecule
VCAM1	SHH	Molecule	Molecule
BP-7,8-diol	CYP3A4	Molecule	Molecule
ATF3	SULT2B1	Molecule	Molecule
CASP9	PRODH	Molecule	Molecule
MAPK3	JMJD7-PLA2G4B	Molecule	Molecule
JUN	GSS	Molecule	Molecule
CALR	GCLC	Molecule	Molecule

VEGFA	CYP2E1	Molecule	Molecule
SMAD3	CYP2E1	Molecule	Molecule
EGFR	SHH	Molecule	Molecule
FGR	PIK3CG	Molecule	Molecule
ASS1	aspartic acid	Molecule	Molecule
SIRT1	SHMT1	Molecule	Molecule
TNF	GCLC	Molecule	Molecule
CAMK2B	PRODH	Molecule	Molecule
FOS	GAD1	Molecule	Molecule
TGFB1	SHH	Molecule	Molecule
RXRA	SULT2B1	Molecule	Molecule
IGF1R	PIK3CB	Molecule	Molecule
AR	GNMT	Molecule	Molecule
ALOX5	CYP27A1	Molecule	Molecule
ICAM1	GATM	Molecule	Molecule
PLA2G1B	arachidonic acid	Molecule	Molecule
PIK3CG	PRKCD	Molecule	Molecule
PCNA	ALDH1B1	Molecule	Molecule
PCNA	TP53	Molecule	Molecule
MAPK14	PYGB	Molecule	Molecule
IGF1R	GLUL	Molecule	Molecule
HNRNPK	TP53	Molecule	Molecule
ASNS	VCAM1	Molecule	Molecule
MAP3K1	MAPK14	Molecule	Molecule
NOS2	CALML3	Molecule	Molecule
PDGFRB	PLCG2	Molecule	Molecule
GSK3B	GAD1	Molecule	Molecule
GSTM2	PLA2G2A	Molecule	Molecule
MET	GATM	Molecule	Molecule
CYP1A1	SULT2B1	Molecule	Molecule
NFKB1	NFKBIA	Molecule	Molecule
ATF3	FOXO3	Molecule	Molecule
CYP27A1	cholesterol	Molecule	Molecule
CYP2E1	SULT2B1	Molecule	Molecule
NFKB1	TP53	Molecule	Molecule
FGR	PIK3CB	Molecule	Molecule
IGF1	GAD1	Molecule	Molecule
IL1B	STS	Molecule	Molecule
UGT1A3	TNF	Molecule	Molecule
GPX5	reduced glutathione	Molecule	Molecule
EP300	STAT6	Molecule	Molecule
HSPA1A	GATM	Molecule	Molecule
NOS2	CYP27A1	Molecule	Molecule
BCL2	GAD1	Molecule	Molecule
CXCL2	CYP27A1	Molecule	Molecule
RECK	PTGS2	Molecule	Molecule
PRKCB	GAD1	Molecule	Molecule
PIK3R1	IGF1	Molecule	Molecule
PLA2G2A	PYGB	Molecule	Molecule
PLA2G2A	GCLC	Molecule	Molecule

TGFBR1	PTGS2	Molecule	Molecule
ALOX15	TP53	Molecule	Molecule
MMP9	GCLC	Molecule	Molecule
TGFBR2	GSS	Molecule	Molecule
GSK3B	PRODH	Molecule	Molecule
MMP9	MAPK1	Molecule	Molecule
HRAS	PRKCB	Molecule	Molecule
PIK3CA	SHH	Molecule	Molecule
G6PC2	glucose	Molecule	Molecule
SRC	GNAI3	Molecule	Molecule
GGT1	glutamic acid	Molecule	Molecule
JUN	GGT1	Molecule	Molecule
MAPK9	PLA2G4A	Molecule	Molecule
JUN	PYGL	Molecule	Molecule
STAT3	SULT2B1	Molecule	Molecule
GNAI2	PLA2G4A	Molecule	Molecule
PCK2	glucose	Molecule	Molecule
TLR4	FOS	Molecule	Molecule
VEGFA	ALDH4A1	Molecule	Molecule
TGFBR2	CYP2E1	Molecule	Molecule
reduced glutathione	glutamic acid	Molecule	Molecule
UGT1A10	androsterone	Molecule	Molecule
TLR4	PYGL	Molecule	Molecule
CYP4F3	UBC	Molecule	Molecule
TEK	ALAS2	Molecule	Molecule
HRAS	CAMK2B	Molecule	Molecule
aspartic acid	ASS1	Molecule	Molecule
TLR4	GCLC	Molecule	Molecule
NR1I2	CYP3A4	Molecule	Molecule
CCL4	CYP27A1	Molecule	Molecule
ALDH4A1	L-delta 1-pyrroline-5-carboxylate	Molecule	Molecule
KDR	GAD1	Molecule	Molecule
VEGFA	PYGB	Molecule	Molecule
SND1	IRF1	Molecule	Molecule
PARP1	PCK1	Molecule	Molecule
RECK	TP53	Molecule	Molecule
NFKBIA	GATM	Molecule	Molecule
NCF1	GCLC	Molecule	Molecule
GPX5	5(S-HPETE	Molecule	Molecule
PCK1	glucose	Molecule	Molecule
RECK	FOXO3	Molecule	Molecule
GPX1	CYP2E1	Molecule	Molecule
CASP3	GAD2	Molecule	Molecule
PTK2	MAPK3	Molecule	Molecule
PYGL	glucose	Molecule	Molecule
IRF9	STAT1	Molecule	Molecule
ALOX15	arachidonic acid	Molecule	Molecule
reduced glutathione	gamma-glutamylcysteine	Molecule	Molecule
CTNNB1	PCK1	Molecule	Molecule

SND1	DHCR7	Molecule	Molecule
JUN	SHH	Molecule	Molecule
APAF1	CASP9	Molecule	Molecule
IFNG	MAPK3	Molecule	Molecule
ALAS2	glycine	Molecule	Molecule
PIK3R1	IL2RA	Molecule	Molecule
APAF1	PTGS2	Molecule	Molecule
PRKCE	PLA2G6	Molecule	Molecule
FOS	PTGS2	Molecule	Molecule
NR1I2	TP53	Molecule	Molecule
FGR	PIK3R1	Molecule	Molecule
RELA	CDX2	Molecule	Molecule
NOS2	GCLC	Molecule	Molecule
PTK2	PRKCB	Molecule	Molecule
CCL4	PYGB	Molecule	Molecule
CYP3A4	GGT1	Molecule	Molecule
PIK3R1	HRAS	Molecule	Molecule
PTGS2	arachidonic acid	Molecule	Molecule
PTGS2	GATM	Molecule	Molecule
AKT1	PRODH	Molecule	Molecule
ATF3	PRODH	Molecule	Molecule
SRC	EGFR	Molecule	Molecule
NR1I2	GOT1	Molecule	Molecule
PTGS2	CYP2E1	Molecule	Molecule
STAT3	GSS	Molecule	Molecule
CXCL8	SHMT1	Molecule	Molecule
INSR	PLA2G16	Molecule	Molecule
PRKCE	PLCB1	Molecule	Molecule
GOT1	aspartic acid	Molecule	Molecule
CYP17A1	20(S-hydroxycholesterol	Molecule	Molecule
TP53	GCLC	Molecule	Molecule
ERBB2	MYC	Molecule	Molecule
TLR4	MAPK3	Molecule	Molecule
OPLAH	pyroglutamate	Molecule	Molecule
MMP9	PYGL	Molecule	Molecule
CYP3A4	linoleic acid	Molecule	Molecule
UGT1A8	estradiol	Molecule	Molecule
MAP3K1	PRKCB	Molecule	Molecule
cholesterol	LIPA	Molecule	Molecule
CASP3	GCLC	Molecule	Molecule
EGFR	PIK3CG	Molecule	Molecule
HMOX1	GCLC	Molecule	Molecule
SYK	PIK3CB	Molecule	Molecule
ZAP70	MAPK14	Molecule	Molecule
EP300	TP53	Molecule	Molecule
EGFR	PIK3CB	Molecule	Molecule
TP53	TBP	Molecule	Molecule
1,2-dibromoethane	CYP2E1	Molecule	Molecule
HNRNPK	MYC	Molecule	Molecule
CXCL8	PYGL	Molecule	Molecule

CD14	GNA12	Molecule	Molecule
SRC	CYP17A1	Molecule	Molecule
TGFBR2	GATM	Molecule	Molecule
NFKBIB	NFKB1	Molecule	Molecule
SYK	GSTM1	Molecule	Molecule
GPX1	reduced glutathione	Molecule	Molecule
TGFB1	GCLC	Molecule	Molecule
PIK3CA	PYGL	Molecule	Molecule
MAP3K8	NFKB1	Molecule	Molecule
GCAT	FBXO6	Molecule	Molecule
SND1	GAD1	Molecule	Molecule
SIRT1	PCK1	Molecule	Molecule
CASP3	SHMT1	Molecule	Molecule
GSTM2	PYGB	Molecule	Molecule
PARP1	GLUL	Molecule	Molecule
HNRNPK	PTGS2	Molecule	Molecule
CDK2	PTGS2	Molecule	Molecule
reduced glutathione	glycine	Molecule	Molecule
ZAP70	PIK3CA	Molecule	Molecule
IL1B	GCLC	Molecule	Molecule
SMAD2	GPX1	Molecule	Molecule
MAPK1	PLA2G2A	Molecule	Molecule
UGT1A3	GAD1	Molecule	Molecule
SIRT1	SULT2B1	Molecule	Molecule
PRKCE	ACTB	Molecule	Molecule
GSTM1	GOT1	Molecule	Molecule
UGT1A7	estradiol	Molecule	Molecule
CALML3	PYGL	Molecule	Molecule
PLA2G4F	linoleic acid	Molecule	Molecule
PLA2G2A	CYP27A1	Molecule	Molecule
IRF9	FOS	Molecule	Molecule
CD247	FCGR3A	Molecule	Molecule
HRAS	PYGL	Molecule	Molecule
GSTP1	reduced glutathione	Molecule	Molecule
CAD	aspartic acid	Molecule	Molecule
VCAM1	GATM	Molecule	Molecule
ATF3	SHMT1	Molecule	Molecule
NR1I2	PTGS2	Molecule	Molecule
BCL2L1	PYGL	Molecule	Molecule
CYP1B1	SHMT1	Molecule	Molecule
UGT1A10	chloroform/methanol	Molecule	Molecule
MYC	PCK1	Molecule	Molecule
PARP1	CYP2E1	Molecule	Molecule
SMAD2	GNMT	Molecule	Molecule
ALOX15	GPX1	Molecule	Molecule
ALOX5	GSTP1	Molecule	Molecule
PLCG2	PLA2G4A	Molecule	Molecule
ICAM1	GAD2	Molecule	Molecule
CYP1A2	GGT1	Molecule	Molecule
CCL4	PLA2G2A	Molecule	Molecule

SND1	DAO	Molecule	Molecule
GNAI3	PLA2G4A	Molecule	Molecule
PTPA	glucose	Molecule	Molecule
TP53	PTGS2	Molecule	Molecule
CRK	GCLC	Molecule	Molecule
glycine	LAP3	Molecule	Molecule
CCL4	GCLC	Molecule	Molecule
CYP2E1	arachidonic acid	Molecule	Molecule
LAP3	3H-proline	Molecule	Molecule
NFKBIA	PYGB	Molecule	Molecule
ERBB2	PTPA	Molecule	Molecule
IL2RA	PRODH	Molecule	Molecule
ATF2	CYP11A1	Molecule	Molecule
CRK	PYGB	Molecule	Molecule
FOS	GCLC	Molecule	Molecule
arachidonic acid	CYP2E1	Molecule	Molecule
EGFR	MAPK3	Molecule	Molecule
TP53	PTPA	Molecule	Molecule
CYP1B1	BP-7,8-diol	Molecule	Molecule
IL1R1	CASP3	Molecule	Molecule
C14787	CYP2E1	Molecule	Molecule
ACTB	SHH	Molecule	Molecule
PTK2	SHH	Molecule	Molecule
GPX1	5(S-HPETE	Molecule	Molecule
SIRT1	TP53	Molecule	Molecule
ICAM1	GCLC	Molecule	Molecule
GLUL	glutamic acid	Molecule	Molecule
MAPK11	NFATC2	Molecule	Molecule
PARP1	SULT2B1	Molecule	Molecule
TGFB1	PCK1	Molecule	Molecule
HNRNPK	SHMT1	Molecule	Molecule
NCF1	PRKCB	Molecule	Molecule
PCNA	POLD1	Molecule	Molecule
aspartic acid	GOT1	Molecule	Molecule
SP1	SULT2B1	Molecule	Molecule
RXRB	ALDH7A1	Molecule	Molecule
PLA2G6	arachidonic acid	Molecule	Molecule
pyroglutamate	OPLAH	Molecule	Molecule
TNF	CAD	Molecule	Molecule
PLA2G2A	arachidonic acid	Molecule	Molecule
FCGR1A	PTGS2	Molecule	Molecule
IGF1R	SHMT1	Molecule	Molecule
TP53	GAD1	Molecule	Molecule
MAPK11	JUND	Molecule	Molecule
citrulline	ASS1	Molecule	Molecule
CYCS	CASP8	Molecule	Molecule
PLA2G16	linoleic acid	Molecule	Molecule
MMP9	GATM	Molecule	Molecule
NCF1	TP53	Molecule	Molecule
TGFBR2	SULT2B1	Molecule	Molecule

PRKCE	PYGB	Molecule	Molecule
20(S-hydroxycholesterol	cholesterol	Molecule	Molecule
UGT1A3	DAO	Molecule	Molecule
GSTM2	MIR122	Molecule	Molecule
CDKN1A	PTPA	Molecule	Molecule
UGT1A8	chloroform/methanol	Molecule	Molecule
ALOX5	TP53	Molecule	Molecule
PLA2G2A	GATM	Molecule	Molecule
CD14	GSTP1	Molecule	Molecule
beta-alanine	aspartic acid	Molecule	Molecule
TGFB1	PYGL	Molecule	Molecule
APAF1	GSTP1	Molecule	Molecule
CCL4	GATM	Molecule	Molecule
MAPK11	ATF2	Molecule	Molecule
estradiol	AR	Molecule	Molecule
SND1	PTGS2	Molecule	Molecule
INSR	PIK3CB	Molecule	Molecule
PRKCE	GATM	Molecule	Molecule
TP53	SULT2B1	Molecule	Molecule
PLA2G4F	calcium ions	Molecule	Molecule
CD14	CYP27A1	Molecule	Molecule
MAPK11	GNAI3	Molecule	Molecule
FOXO3	GCLC	Molecule	Molecule
VCAM1	GCLC	Molecule	Molecule
RECK	SP1	Molecule	Molecule
PCNA	TNF	Molecule	Molecule
IL6R	PRODH	Molecule	Molecule
GAD2	glutamic acid	Molecule	Molecule
HNF4A	PCK1	Molecule	Molecule
SMAD2	MYC	Molecule	Molecule
CYCS	CASP9	Molecule	Molecule
SND1	PLA2G4A	Molecule	Molecule
arachidonic acid	CYP4F3	Molecule	Molecule
IL1R1	RELA	Molecule	Molecule
GSS	gamma-glutamylcysteine	Molecule	Molecule
STAT6	PTGS2	Molecule	Molecule
JUN	PLA2G4A	Molecule	Molecule
CDKN1A	SULT2B1	Molecule	Molecule
MAPK11	FCGR1A	Molecule	Molecule
CASP9	calcium ions	Molecule	Molecule
TGFB1	GNMT	Molecule	Molecule
INSR	GSTA4	Molecule	Molecule
GCK	sucrose	Molecule	Molecule
CYCS	BCL2	Molecule	Molecule
CYP1A2	TP53	Molecule	Molecule
NFKB2	NFKBIA	Molecule	Molecule
MET	PTGS2	Molecule	Molecule
CYP2E1	TP53	Molecule	Molecule
BCL2	GATM	Molecule	Molecule
IL1B	PYGB	Molecule	Molecule

MAP3K8	GSTP1	Molecule	Molecule
MAP3K1	MAPK9	Molecule	Molecule
CCND1	SULT2B1	Molecule	Molecule
SYK	PLCG2	Molecule	Molecule
PRKCB	GRIN2B	Molecule	Molecule
ZAP70	CD247	Molecule	Molecule
PTK2B	PRKCB	Molecule	Molecule
PARP1	SHMT1	Molecule	Molecule
GSTM1	reduced glutathione	Molecule	Molecule
TGFBR1	GLS	Molecule	Molecule
TGFBR1	CYP1A2	Molecule	Molecule
CYP1A1	GCLC	Molecule	Molecule
NCF1	GPX1	Molecule	Molecule
ATF3	CYP2E1	Molecule	Molecule
STAT3	GCLC	Molecule	Molecule
CASP3	PTGS2	Molecule	Molecule
GNA12	PYGB	Molecule	Molecule
MAP3K8	GATM	Molecule	Molecule
MAPK14	PLA2G2A	Molecule	Molecule
RECK	GCLC	Molecule	Molecule
BCL2L1	CYP2E1	Molecule	Molecule
SRC	SHMT1	Molecule	Molecule
TGFBR2	PTGS2	Molecule	Molecule
EGFR	GATM	Molecule	Molecule
CALR	GATM	Molecule	Molecule
GAD1	aspartic acid	Molecule	Molecule
CASP8	SHH	Molecule	Molecule
CYP1A1	TP53	Molecule	Molecule
UGT1A7	chloroform/methanol	Molecule	Molecule
CDX2	SI	Molecule	Molecule
STS	cholesterol	Molecule	Molecule
RELA	CYP27A1	Molecule	Molecule
CALR	PLA2G2A	Molecule	Molecule
glutamic acid	OPLAH	Molecule	Molecule
SIRT1	CYP2E1	Molecule	Molecule
GSTA4	1,2-dibromoethane	Molecule	Molecule
CYP1A1	C14787	Molecule	Molecule
GSTP1	PLA2G2A	Molecule	Molecule
MMP9	SULT2B1	Molecule	Molecule
SHC1	TNF	Molecule	Molecule
PTK2	PIK3CB	Molecule	Molecule
CDK2	HCFC1	Molecule	Molecule
SMAD3	MYC	Molecule	Molecule
CYP1A1	SHMT1	Molecule	Molecule
INSR	MAPK3	Molecule	Molecule
AKT1	PPP1CC	Molecule	Molecule
STAT1	GLS	Molecule	Molecule
ALOX15	CYP27A1	Molecule	Molecule
CXCL8	GCLC	Molecule	Molecule
LAP3	UBC	Molecule	Molecule

ALOX15	linoleic acid	Molecule	Molecule
FOXO3	PCK2	Molecule	Molecule
VEGFA	GATM	Molecule	Molecule
NFKB1	MAPK3	Molecule	Molecule
SMAD3	CYP27A1	Molecule	Molecule
FBXO6	PTGS1	Molecule	Molecule
CCND1	ALDH4A1	Molecule	Molecule
VCAM1	CAD	Molecule	Molecule
CASP3	CYP1A2	Molecule	Molecule
RECK	CYP2E1	Molecule	Molecule
NR1I2	PCK1	Molecule	Molecule
ERBB2	GATM	Molecule	Molecule
CDK2	CSNK2A1	Molecule	Molecule
glycine	GCAT	Molecule	Molecule
GCLC	gamma-glutamylcysteine	Molecule	Molecule
FOXO6	G6PC3	Molecule	Molecule
VEGFA	GCLC	Molecule	Molecule
BCL2	CYP2E1	Molecule	Molecule
IL1R1	PLA2G2A	Molecule	Molecule
HMOX1	TP53	Molecule	Molecule
STAT3	IL6R	Molecule	Molecule
HSPA1A	GCLC	Molecule	Molecule
TNF	PRODH	Molecule	Molecule
UBC	PTGS2	Molecule	Molecule
DHCR7	cholesterol	Molecule	Molecule
MAPK10	PLA2G4A	Molecule	Molecule
MAP3K1	CYP17A1	Molecule	Molecule
HNRNPK	GSTP1	Molecule	Molecule
GGCT	UBC	Molecule	Molecule
CDKN1A	ALDH7A1	Molecule	Molecule
15(S-HPETE	ALOX15	Molecule	Molecule
linoleic acid	PLA2G4A	Molecule	Molecule
aspartic acid	ASNS	Molecule	Molecule
IRF9	STAT6	Molecule	Molecule
TGFBR2	GGT1	Molecule	Molecule
androsterone	UGT1A3	Molecule	Molecule
PRKCB	PLA2G4B	Molecule	Molecule
FGR	PTK2B	Molecule	Molecule
RXRA	GNMT	Molecule	Molecule
CSNK2A1	CTNNB1	Molecule	Molecule
MET	PYGL	Molecule	Molecule
PDGFRB	CRK	Molecule	Molecule
IL1B	PRODH	Molecule	Molecule
CASP3	CASP8	Molecule	Molecule
CD14	CYP2E1	Molecule	Molecule
EP300	STAT1	Molecule	Molecule
TGFBR2	TP53	Molecule	Molecule
KDR	PYGB	Molecule	Molecule
MET	GCLC	Molecule	Molecule
CTNNB1	GOT1	Molecule	Molecule

PYGB	glucose	Molecule	Molecule
STAT3	PTGS2	Molecule	Molecule
NFKBIA	AKT1	Molecule	Molecule
GSK3B	PTGS2	Molecule	Molecule
CYP2E1	PYGL	Molecule	Molecule
HSPA1A	PLA2G2A	Molecule	Molecule
AKT1	GAD1	Molecule	Molecule
IGF1	PYGB	Molecule	Molecule
GNMT	glycine	Molecule	Molecule
GCLC	glutamic acid	Molecule	Molecule
BCL2	TP53	Molecule	Molecule
IFNG	GCLC	Molecule	Molecule
IFNG	GOT1	Molecule	Molecule
AKT1	GNMT	Molecule	Molecule
PARP1	PTGS2	Molecule	Molecule
CXCL2	PLA2G2A	Molecule	Molecule
MMP9	MAPK3	Molecule	Molecule
SHMT2	glycine	Molecule	Molecule
PLA2G6	linoleic acid	Molecule	Molecule
TEK	CYP2E1	Molecule	Molecule
calcium ions	PLA2G4F	Molecule	Molecule
PIK3CB	PRODH	Molecule	Molecule
MET	PRODH	Molecule	Molecule
AKT1	GAD2	Molecule	Molecule
GAD2	aspartic acid	Molecule	Molecule
NFKB2	PTGS2	Molecule	Molecule
EGFR	GCLC	Molecule	Molecule
MAPK1	TP53	Molecule	Molecule
CYP11A1	cholesterol	Molecule	Molecule
GOT1	UBC	Molecule	Molecule
linoleic acid	CYP2E1	Molecule	Molecule
SHC1	JUND	Molecule	Molecule
TNF	PLA2G2A	Molecule	Molecule
3H-proline	LAP3	Molecule	Molecule
sucrose	glucose	Molecule	Molecule
PTGS2	SULT2B1	Molecule	Molecule
KDR	GCLC	Molecule	Molecule
HMOX1	GOT1	Molecule	Molecule
ATF3	GCLC	Molecule	Molecule
MAPK3	GAD1	Molecule	Molecule
HRAS	GAD1	Molecule	Molecule
PDGFRB	PIK3CA	Molecule	Molecule
CSNK2A1	GSTM1	Molecule	Molecule
NR1I2	GCLC	Molecule	Molecule
Dyslipidemia	Systemic inflammation	Phenotype	Phenotype
Oxidative stress	Platelet activation	Phenotype	Phenotype
Platelet activation	COX1	Phenotype	Molecule
Platelet activation	ICAM1	Phenotype	Molecule
Platelet activation	VCAM1	Phenotype	Molecule
COX1	Aggregation	Molecule	Phenotype

ICAM1	Adhesion	Molecule	Phenotype
VCAM1	Adhesion	Molecule	Phenotype
COX2	Endothelial inflammation	Molecule	Phenotype
Aggregation	Adhesion	Phenotype	Phenotype
Adhesion	Endothelial inflammation	Phenotype	Phenotype
Systemic inflammation	Endothelial inflammation	Phenotype	Phenotype
Aggregation	Thrombosis	Phenotype	Phenotype
Thrombosis	Atherosclerosis	Phenotype	Phenotype
Endothelial inflammation	Atherosclerosis	Phenotype	Phenotype
Atherosclerosis	Myocardial infactions	Phenotype	Phenotype
Myocardial infactions	Heart failure	Phenotype	Phenotype
Cardiac dysfunction	Heart failure	Phenotype	Phenotype

Supplementary Table S3. The list of target gene names

ID	Component	Target	Gene name	Related functions
1	Ellagic acid, Salvianolic acid B	AKT serine/threonine kinase 1	AKT1	Endothelial inflammation
2	Ellagic acid, Tanshinone IIA	Apoptotic peptidase activating factor 1	APAF1	Platelet activation
3	Ellagic acid, Tanshinone IIA	Activating transcription factor 3	ATF3	Platelet activation
4	Ellagic acid, Protocatechuic acid, Salvianolic acid B, Tanshinone I	Apoptosis regulator	BCL2	Endothelial inflammation
5	Ellagic acid, Salvianolic acid B, Salvianolic acid A	BCL2 like 1	BCL2L1	Endothelial inflammation
6	Ellagic acid, Caffeic acid, Protocatechuic acid, Salvianolic acid A, Salvianolic acid B, Tanshinone IIA	Caspase 3	CASP3	Endothelial inflammation
7	Ellagic acid	CD14 molecule	CD14	Endothelial inflammation
8	Caffeic acid	Cyclin dependent kinase 2	CDK2	Endothelial inflammation
9	Ellagic acid	Casein kinase 2 alpha 1	CSNK2A1	Endothelial inflammation
10	Salvianolic acid B	Cytochrome c, somatic	CYCS	Endothelial inflammation
11	Ellagic acid	Cytochrome P450 family 1 subfamily B member 1	CYP1B1	Endothelial inflammation
12	Protocatechuic acid, Caffeic acid	Cytochrome P450 family 2 subfamily E member 1	CYP2E1	Endothelial inflammation
13	Ellagic acid	Fos proto-oncogene, AP-1 transcription factor subunit	FOS	Endothelial inflammation
14	Ellagic acid	Glycogen synthase kinase 3 beta	GSK3B	Endothelial inflammation
15	Ellagic acid	Heterogeneous nuclear ribonucleoprotein K	HNRNPK	Endothelial inflammation
16	Fumaric acid, Cryptotanshinone	Intercellular adhesion molecule 1	ICAM-1	Adhesion molecule production
17	Ellagic acid	Jun proto-oncogene, AP-1 transcription factor subunit	JUN	Endothelial inflammation
18	Ellagic acid	MET proto-oncogene, receptor tyrosine kinase	MET	Endothelial inflammation
19	Ellagic acid	Nuclear factor kappa B subunit 1	NFKB1	Endothelial inflammation
20	Ellagic acid	Nuclear factor kappa B subunit 2	NFKB2	Endothelial inflammation
21	Ellagic acid	Proliferating cell nuclear antigen	PCNA	Endothelial inflammation
22	Ellagic acid	Phospholipase A2 group IIA	PLA2G2A	Adhesion molecule production
23	Ellagic acid	Reversion inducing cysteine rich protein with kazal motifs	RECK	Endothelial inflammation
24	Ellagic acid, Fumaric acid, Protocatechuic acid, Danshensu	RELA proto-oncogene, NF-kB subunit	RELA	Endothelial inflammation

25	Tanshinone IIA	Retinoid X receptor beta	RXRB	Endothelial inflammation
26	Salvioanolic acid A	Sirtuin 1	SIRT1	Endothelial inflammation
27	Ellagic acid	Signal transducer and activator of transcription 3	STAT3	Endothelial inflammation
28	Ellagic acid, Salvianolic acid B	Transforming growth factor beta receptor 1	TGFBR1	Endothelial inflammation
29	Ellagic acid, Salvianolic acid B	Transforming growth factor beta receptor 2	TGFBR2	Endothelial inflammation
30	Caffeic acid, Tanshinone IIA	Tumor protein p53	TP53	endothelial inflammation
31	Fumaric acid, Cryptotanshinone	Vascular cell adhesion molecule 1	VCAM-1	adhesion molecule production
32	Protocatechuic acid	Vascular endothelial growth factor A	VEGFA	Platelet activation

Supplementary Table S4. The functional annotation and pathway enrichment analysis

Component	Path Name	Enriched Gene&MB	P-value	FDR	Path Category	Endothelial inflammation	Adhesion molecule production	Platelet activation
EA	Pathways in cancer	BCL2L1|PTGS2|PTK2|PDGFRB|HRAS|NFKBIA|EGFR|SMAD3|CTNNB1|AKT1|MAPK10|MMP9|CASP9|STAT3|STAT1|PIK3CA|PIK3CB|JUN|NTRK1|PIK3CG|MAPK3|PIK3R5|MAPK1|PIK3R1|NFKB1|MYC|MAPK9|CDKN1A|TGFB1|IGF1R|IGF1|FOS|PRKCB|PLCG2|SHH|EP300|NFKB2|RELA|TP53|CASP3|calcium ions|CCND1|CXCL8|ERBB2|CASP8|GSK3B|MET|cholesterol|BCL2	1.70E-23	1.46E-21	Cancers	AKT1, BCL2, BCL2L1, CASP3, FOS, GSK3B, JUN, MET, NFKB1, NFKB2, RELA, STAT3, TP53		
CA	Metabolic pathways	PCK2|PTGS2|PTGS1|glycine|LAP3|GSS|G6PC3|GCLC|ALOX15|PYCR2|CYP4F3|ASS1|GAD1|GAD2|CAD|CYP27A1|3H-proline|DAO|reduced glutathione|GATM|ALAS2|SHMT1|estradiol|GOT1|arachidonic acid|glucose|linoleic acid|NOS3|DHEA|ALOX5|estrone|glutamic acid|GANC|GGT1|ALDH7A1|gamma-glutamylcysteine|beta-alanine|oxaloacetate|CYP1A1|CYP1A2|CYP7A1|ASNS|CYP2E1|PRODH|cholesterol|aspartic acid	7.46E-06	0.000191846	Cancers	CYP2E1		
PCA	Metabolic pathways	PTGS2|PTGS1|glycine|LAP3|GSS|GCLC|PYCR2|CYP4F3|ASS1|GAD1|GAD2|CAD|PRODH|3H-proline|DAO|reduced glutathione|GATM|DHEA|citrulline|GOT1|arachidonic acid|glucose|linoleic acid|NOS2|NOS3|GCK|glutamic acid|GANC|GGT1|gamma-glutamylcysteine|SHMT1|DHCR24|CYP1A1|CYP1A2|ALDH4A1|L-delta 1-pyrroline-5-carboxylate|ASNS|CYP2E1|CYP27A1|SI|sucrose|cholesterol|aspartic acid	4.97E-07	3.87E-06	Immune system	CYP2E1		
SB	Metabolic pathways	PTGS2|PTGS1|PCK1|glycine|LAP3|GSS|CYCS|GCLC|GLUL|PYCR2|GLS|ASS1|GAD1|GAD2|CAD|CYP27A1|CYP4F3|reduced glutathione|GATM|citrulline|GOT1|arachidonic acid|glucose|linoleic acid|NOS2|NOS3|glutamic acid|GANC|3H-proline|gamma-glutamylcysteine|SHMT1|DHCR24|ASNS|CYP2E1|PRODH|GGT1|cholesterol|ALOX15|aspartic acid	2.34E-05	0.000177011	Cancers	CYCS, CYP2E1		

TIIA	Metabolic pathways	PTGS2|PTGS1|PCK1|glycine|LAP3|GCLC|CYP17A1|PYCR2|CYP4F3|ASS1|GAD1|GAD2|CAD|3H-proline|reduced glutathione|GATM|ALAS2|GOT1|arachidonic acid|glucose|linoleic acid|NOS3|glutamic acid|GANC|GGT1|ALDH7A1|gamma-glutamylcysteine|G6PC2|beta-alanine|SHMT1|CYP2C9|ALDH4A1|L-delta 1-pyrroline-5-carboxylate|ASNS|CYP2E1|PRODH|SI|cholesterol|aspartic acid	2.91E-06	7.59E-05	Immune system	CYP2E1	
EA	Hepatitis B	TGFB1|NFATC2|HRAS|NFKBIA|SMAD3|AKT1|PTK2B|MAPK10|STAT6|STAT3|STAT1|PIK3CA|PIK3CB|MAP3K1|JUN|PIK3CG|MAPK3|PIK3R5|MAPK1|PIK3R1|NFKB1|MYC|MAPK9|CDKN1A|APAF1|SRC|TGFBR1|FOS|PRKCB|TNF|EP300|RELA|TP53|CASP3|CASP9|MMP9|BCL2|ATF2	2.17E-26	5.57E-24	Cancers	AKT1, BCL2, CASP3, FOS, JUN, NFKB1, RELA, STAT3, TGFBR1, TP53	APAF1
TI	Metabolic pathways	PTGS2|PTGS1|PCK1|glycine|LAP3|reduced glutathione|CYP17A1|PYCR2|CYP4F3|ASS1|GAD1|CAD|PRODH|3H-proline|GCLC|GATM|SHMT1|GOT1|arachidonic acid|glucose|linoleic acid|NOS3|GCK|glutamic acid|GANC|GGT1|gamma-glutamylcysteine|CYP3A4|DHEA|CYP1A1|CYP1A2|ASNS|CYP2E1|CYP27A1|SI|sucrose|cholesterol|aspartic acid	3.30E-09	4.24E-07	Infectious diseases	CYP2E1	
EA	MAPK signaling pathway	PDGFRB|TGFB1|HRAS|EGFR|JUND|MAPK14|AKT1|HSPA1A|MAPK10|MAPK11|CRK|IL1B|IL1R1|MAP3K8|MAP3K1|JUN|NTRK1|PLA2G4F|MAPK3|MAPK1|NFKB1|NFKB2|MAPK9|TGFBR2|TGFBR1|PRKCB|TNF|PLA2G4B|PLA2G4A|MYC|RELA|TP53|CASP3|calcium ions|CD14|GNA12|ATF2	2.44E-14	2.73E-13	Cancers	AKT1, CASP3, CD14, JUN, NFKB1, NFKB2, RELA, TGFBR1, TGFBR2, TP53	
CT	Metabolic pathways	PTGS2|PTGS1|glycine|LAP3|PYCR2|CYP4F3|ASS1|GAD2|CAD|CYP27A1|3H-proline|GCLC|GATM|SHMT1|GOT1|arachidonic acid|glucose|linoleic acid|NOS3|GCK|glutamic acid|GANC|GGT1|gamma-glutamylcysteine|CYP3A4|DHEA|CYP1A1|CYP1A2|ASNS|CYP2E1|PRODH|SI|sucrose|cholesterol|aspartic acid	2.68E-09	6.89E-07	Cancers	CYP2E1	
EA	PI3K-Akt signaling pathway	BCL2L1|PCK2|PCK1|PDGFRB|CDKN1A|EGFR|G6PC3|G6PC2|FOXO3|AKT1|INSR|PTK2|CASP9|TEK|MET|PIK3CA|PIK3CB|NOS3|MAPK3|MAPK1|PIK3R1|NFKB1|MYC|HRAS|KDR|IGF1R|IGF1|RELA|TP53|SYK|IRS1|GSK3B|CCND1|BCL2|ATF2	5.34E-17	1.06E-15	Cancers	AKT1, BCL2, BCL2L1, GSK3B, MET, NFKB1, RELA, TP53	

EA	Osteoclast differentiation	TGFB1|NFKBIA|FCGR3A|JUND|MAPK14|AKT1|MAPK10|MAPK11|FCGR1A|IL1B|IL1R1|STAT1|PIK3CA|PIK3CB|JUN|PIK3CG|MAPK3|PIK3R5|MAPK1|PIK3R1|NFKB1|RELA|MAPK9|TGFBR2|TGFBR1|FOS|FCGR2A|PLCG2|TNF|NFKB2|calcium ions|SYK|IRF9	1.41E-23	1.46E-21	Cancers	AKT1, FOS, JUN, NFKB1, NFKB2, RELA, TGFBR1, TGFBR2		
EA	HTLV-I infection	PDGFRB|NFATC2|TGFB1|HRAS|NFKBIA|SMAD3|AKT1|IL1R1|DLG1|PIK3CA|PIK3CB|MAP3K1|JUN|PIK3CG|POLD1|PIK3R5|ICAM1|NFKB1|RELA|CDKN1A|PCNA|TGFBR2|TGFBR1|PIK3R1|TNF|EP300|NFKB2|TP53|calcium ions|CALR|GSK3B|ATF3|ATF2	3.11E-16	5.33E-15	Endocrine system	AKT1, GSK3B, JUN, NFKB1, NFKB2, PCNA, RELA, TGFBR1, TGFBR2, TP53	ICAM1	ATF3
SA	Metabolic pathways	PTGS2|PTGS1|PCK1|glycine|LAP3|PYCR2|CYP4F3|ASS1|GAD2|CAD|CYP27A1|3H-proline|GCLC|GATM|citrulline|GOT1|arachidonic acid|glucose|linoleic acid|NOS2|NOS3|glutamic acid|GANC|gamma-glutamylcysteine|SHMT1|CYP1A2|ASNS|CYP2E1|PRODH|ANPEP|cholesterol|aspartic acid	4.20E-05	0.000830051	Cancers	CYP2E1		
EA	Proteoglycans in cancer	TGFB1|PTK2|HRAS|EGFR|MAPK14|PLCG2|MAPK11|STAT3|PIK3CA|PIK3CB|PIK3CG|MAPK3|PIK3R5|MAPK1|PIK3R1|MYC|CDKN1A|KDR|ERBB2|SRC|IGF1R|IGF1|PRKCB|PPP1CC|CTNNB1|TP53|CASP3|AKT1|CCND1|MET|MMP9|ACTB	5.59E-13	4.23E-12	Nervous system	AKT1, CASP3, MET, STAT3, TP53		
EA	Ras signaling pathway	BCL2L1|PDGFRB|HRAS|EGFR|AKT1|INSR|MAPK10|PLA2G1B|TEK|PIK3CA|PIK3CB|PIK3CG|PLA2G6|MAPK3|PIK3R5|MAPK1|ZAP70|PIK3R1|NFKB1|RELA|MAPK9|KDR|IGF1R|IGF1|PLA2G2A|PLA2G4B|PLA2G4A|PLA2G4F|PLA2G16|calcium ions|PLCG2|MET	3.40E-12	2.13E-11	Nervous system	AKT1, BCL2L1, MET, NFKB1, RELA	PLA2G2A	
EA	FoxO signaling pathway	PCK2|TGFB1|PCK1|HRAS|EGFR|TGFBR1|G6PC3|SMAD3|MAPK14|AKT1|INSR|MAPK10|MAPK11|STAT3|PIK3CA|PIK3CB|PIK3CG|MAPK3|PIK3R5|MAPK1|PIK3R1|MAPK9|CDKN1A|TGFBR2|IGF1R|IGF1|glutamic acid|G6PC2|FOXO3|CCND1|IRS1|FOXO6	1.23E-19	4.52E-18	Signal transduction	AKT1, STAT3, TGFBR1, TGFBR2		
EA	Insulin resistance	PCK2|PCK1|NFKBIA|G6PC3|G6PC2|AKT1|INSR|MAPK10|STAT3|PPP1R3A|PIK3CA|PIK3CB|PIK3R5|PIK3CG|PYGB|PIK3R1|NFKB1|RELA|MAPK9|PYGL|glucose|NOS3|PRKCB|PRKCE|PRKCD|TNF|PPP1CC|IRS1|GSK3B|PTPA	4.26E-21	2.73E-19	Carbohydrate metabolism	AKT1, GSK3B, NFKB1, RELA, STAT3		

FA	Metabolic pathways	PTGS2|PTGS1|glycine|LAP3|PYCR2|CYP4F3|ASS1|GAD1|GAD2|CAD|PRODH|3H-proline|GCLC|GATM|GOT1|arachidonic acid|glucose|linoleic acid|NOS3|glutamic acid|GANC|gamma-glutamylcysteine|SHMT1|ASNS|CYP2E1|CYP27A1|SI|cholesterol|aspartic acid	1.46E-05	0.000178747	Cardiovascular diseases	CYP2E1
EA	Focal adhesion	PDGFRB|PTK2|HRAS|EGFR|CTNNB1|AKT1|MAPK10|CRK|PIK3CA|PIK3CB|JUN|PIK3CG|MAPK3|PIK3R5|MAPK1|PIK3R1|MAPK9|KDR|ERBB2|SRC|IGF1R|IGF1|PRKCB|PPP1CC|CCND1|GSK3B|MET|ACTB|BCL2	2.12E-15	3.20E-14	Endocrine system	AKT1, BCL2, GSK3B, JUN, MET
EA	Neurotrophin signaling pathway	HRAS|NFKBIA|MAPK14|AKT1|MAPK10|MAPK11|CRK|PIK3CA|PIK3CB|MAP3K1|JUN|NTRK1|PIK3CG|MAPK3|PIK3R5|MAPK1|PIK3R1|NFKB1|RELA|MAPK9|PRKCD|PLCG2|FOXO3|TP53|calcium ions|IRS1|GSK3B	9.98E-16	1.60E-14	Carbohydrate metabolism	AKT1, GSK3B, JUN, NFKB1, RELA, TP53
EA	Epstein-Barr virus infection	CDKN1A|NFKBIA|MAPK14|PLCG2|MAPK10|MAPK11|GTF2E2|STAT3|PIK3CA|PIK3CB|JUN|FGR|PIK3CG|PIK3R5|PIK3R1|NFKB1|NFKB2|MAPK9|SND1|EP300|MYC|RELA|TP53|AKT1|SYK|GSK3B|ATF2	5.68E-20	2.92E-18	Excretory system	AKT1, GSK3B, JUN, NFKB1, NFKB2, RELA, STAT3, TP53
EA	Chemokine signaling pathway	PTK2|SRC|HRAS|NFKBIA|FOXO3|AKT1|PTK2B|CRK|GNAI3|STAT3|STAT1|PIK3CA|PIK3CB|FGR|PIK3CG|MAPK3|PIK3R5|MAPK1|PIK3R1|NFKB1|RELA|PLCB1|PRKCB|PRKCD|calcium ions|CXCL8|GSK3B	1.60E-10	8.04E-10	Infectious diseases	AKT1, GSK3B, NFKB1, RELA, STAT3
EA	Oxytocin signaling pathway	PTGS2|NFATC2|SRC|HRAS|EGFR|GNAI3|PIK3CA|PIK3CB|JUN|PIK3CG|MAPK3|PIK3R5|MAPK1|PIK3R1|CDKN1A|arachidonic acid|PLCB1|NOS3|FOS|PRKCB|PLA2G4B|PLA2G4A|PLA2G4F|PPP1CC|calcium ions|CCND1|ACTB	8.13E-12	4.86E-11	Metabolism of other amino acids	FOS, JUN
EA	Inflammatory mediator regulation of TRP channels	SRC|MAPK14|PLCG2|MAPK10|MAPK11|IL1B|IL1R1|PIK3CA|PIK3CB|NTRK1|PIK3CG|PLA2G6|PIK3R5|PIK3R1|MAPK9|arachidonic acid|PLCB1|IGF1|PRKCB|PRKCE|PRKCD|PLA2G4B|PLA2G4A|PLA2G4F|PPP1CC|calcium ions	1.37E-13	1.25E-12	Endocrine system	
EA	Prostate cancer	PDGFRB|HRAS|NFKBIA|EGFR|CTNNB1|AKT1|CASP9|PIK3CA|PIK3CB|PIK3CG|MAPK3|PIK3R5|MAPK1|PIK3R1|NFKB1|RELA|CDKN1A|ERBB2|IGF1R|IGF1|EP300|TP53|CCND1|GSK3B|GSTP1|BCL2	2.99E-19	9.61E-18	Global and overview maps	AKT1, BCL2, GSK3B, NFKB1, RELA, TP53

EA	Rap1 signaling pathway	PDGFRB|HRAS|EGFR|MAPK14|AKT1|INSR|MAPK11|CRK|GNAI3|TEK|PIK3CA|PIK3CB|PIK3CG|MAPK3|PIK3R5|MAPK1|PIK3R1|KDR|PLCB1|IGF1R|IGF1|PRKCB|CTNNB1|calcium ions|MET|ACTB	2.94E-09	1.40E-08	Infectious diseases	AKT1, MET
EA	MicroRNAs in cancer	PTGS2|PDGFRB|HRAS|EGFR|RECK|PLCG2|CRK|GLS|STAT3|PIK3CA|MAPK1|NFKB1|MYC|CDKN1A|ERBB2|PRKCB|PRKCE|TP53|CASP3|CCND1|HMOX1|IRS1|MET|MMP9|HNRNPK|BCL2	6.33E-07	2.32E-06	Metabolism of other amino acids	BCL2, CASP3, HNRNPK, MET, NFKB1, RECK, STAT3, TP53
EA	Hepatitis C	HRAS|NFKBIA|EGFR|MAPK14|AKT1|MAPK10|MAPK11|STAT3|STAT1|PIK3CA|PIK3CB|PIK3CG|MAPK3|PIK3R5|MAPK1|PIK3R1|NFKB1|RELA|MAPK9|CDKN1A|TNF|IRF1|TP53|IRF9|GSK3B	3.54E-19	1.01E-17	Cell growth and death	AKT1, GSK3B, NFKB1, RELA, STAT3, TP53
EA	Platelet activation	PTGS1|SRC|MAPK14|PLCG2|MAPK11|GNAI3|PIK3CA|PIK3CB|PIK3CG|MAPK3|PIK3R5|MAPK1|AKT1|PIK3R1|arachidonic acid|PLCB1|NOS3|FCGR2A|PLA2G4B|PLA2G4A|PLA2G4F|PPP1CC|calcium ions|SYK|ACTB	2.19E-12	1.41E-11	Endocrine and metabolic diseases	AKT1
DA	Metabolic pathways	PTGS2|PTGS1|glycine|LAP3|PYCR2|CYP4F3|ASS1|GAD1|PRODH|3H-proline|GCLC|GOT1|arachidonic acid|glucose|linoleic acid|NOS3|GANC|gamma-glutamylcysteine|SHMT1|ASNS|CYP2E1|CYP27A1|SI|cholesterol|aspartic acid	4.81E-06	0.000205954	Metabolism of other amino acids	CYP2E1
EA	T cell receptor signaling pathway	NFATC2|HRAS|NFKBIA|MAPK14|AKT1|CD247|MAPK11|DLG1|MAP3K8|PIK3CA|PIK3CB|JUN|PIK3CG|MAPK3|PIK3R5|MAPK1|ZAP70|PIK3R1|NFKB1|RELA|MAPK9|FOS|calcium ions|GSK3B	2.88E-15	4.12E-14	Cancers	AKT1, FOS, GSK3B, JUN, NFKB1, RELA
EA	Toll-like receptor signaling pathway	NFKBIA|MAPK14|AKT1|MAPK10|MAPK11|IL1B|MAP3K8|STAT1|PIK3CA|PIK3CB|JUN|PIK3CG|MAPK3|PIK3R5|MAPK1|PIK3R1|NFKB1|RELA|MAPK9|FOS|TNF|CXCL8|CASP8|CD14	1.34E-14	1.64E-13	Circulatory system	AKT1, CD14, FOS, JUN, NFKB1, RELA
EA	Chagas disease (American trypanosomiasis)	TGFB1|NFKBIA|MAPK14|AKT1|CD247|MAPK10|MAPK11|GNAI3|PIK3CA|PIK3CB|JUN|PIK3CG|MAPK3|PIK3R5|MAPK1|PIK3R1|NFKB1|RELA|MAPK9|PLCB1|FOS|TNF|CALR|CASP8	3.01E-16	5.33E-15	Infectious diseases	AKT1, FOS, JUN, NFKB1, RELA
EA	Prolactin signaling pathway	HRAS|FOXO3|CYP17A1|MAPK10|MAPK11|STAT3|STAT1|PIK3CA|PIK3CB|PIK3CG|MAPK3|PIK3R5|MAPK1|PIK3R1|NFKB1|RELA|MAPK9|SRC|FOS|MAPK14|IRF1|AKT1|CCND1|GSK3B	3.95E-18	1.01E-16	Infectious diseases	AKT1, FOS, GSK3B, NFKB1, RELA, STAT3

EA	VEGF signaling pathway	PTGS2|NFATC2|PTK2|HRAS|MAPK14|PLCG2|MAPK11|PIK3CA|PIK3CB|PIK3CG|MAPK3|PIK3R5|MAPK1|PIK3R1|KDR|SRC|NOS3|PRKCB|PLA2G4B|PLA2G4A|PLA2G4F|AKT1|calcium ions|CASP9	1.02E-19	4.36E-18	Signal transduction	AKT1
EA	Toxoplasmosis	NFKBIA|MAPK14|AKT1|HSPA1A|MAPK10|MAPK11|GNAI3|STAT3|STAT1|PIK3CA|PIK3CB|PIK3CG|MAPK3|PIK3R5|MAPK1|PIK3R1|NFKB1|RELA|MAPK9|TNF|CASP3|CASP8|CASP9	1.86E-14	2.17E-13	Cancers	AKT1, CASP3, NFKB1, RELA, STAT3
EA	Sphingolipid signaling pathway	HRAS|MAPK14|AKT1|MAPK10|MAPK11|GNAI3|PIK3CA|PIK3CB|PIK3CG|MAPK3|PIK3R5|MAPK1|PIK3R1|NFKB1|RELA|MAPK9|PLCB1|NOS3|PRKCE|TNF|TP53|GNA12|BCL2	2.64E-13	2.12E-12	Cell growth and death	AKT1, BCL2, NFKB1, RELA, TP53
EA	Insulin signaling pathway	PCK2|PCK1|HRAS|G6PC3|G6PC2|AKT1|INSR|MAPK10|CRK|PPP1R3A|PIK3CA|PIK3CB|PYGB|PIK3CG|MAPK3|PIK3R5|MAPK1|PIK3R1|MAPK9|PYGL|PPP1CC|IRS1|GSK3B	1.96E-10	9.68E-10	Endocrine system	AKT1, GSK3B
EA	Influenza A	NFKBIA|MAPK14|AKT1|MAPK10|MAPK11|IL1B|GSK3B|STAT1|PIK3CA|PIK3CB|JUN|PIK3CG|MAPK3|PIK3R5|MAPK1|PIK3R1|NFKB1|RELA|MAPK9|IRF9|CASP9|ATF2	1.18E-13	1.12E-12	Amino acid metabolism	AKT1, GSK3B, JUN, NFKB1, RELA
EA	Fc gamma R-mediated phagocytosis	FCGR3A|PLCG2|FCGR1A|CRK|PIK3CA|PIK3CB|PIK3CG|PLA2G6|MAPK3|PIK3R5|MAPK1|AKT1|PIK3R1|PRKCB|PRKCE|PRKCD|FCGR2A|PLA2G4B|PLA2G4A|PLA2G4F|calcium ions|SYK	1.51E-13	1.34E-12	Cancers	AKT1
EA	Thyroid hormone signaling pathway	SRC|HRAS|CTNNB1|PLCG2|GSK3B|STAT1|PIK3CA|PIK3CB|PIK3CG|MAPK3|PIK3R5|MAPK1|AKT1|PIK3R1|MYC|PLCB1|PRKCB|TP53|calcium ions|CCND1|CASP9|ACTB	1.18E-13	1.12E-12	Cancers	AKT1, GSK3B, TP53
EA	Pancreatic cancer	BCL2L1|EGFR|SMAD3|AKT1|MAPK10|STAT3|STAT1|PIK3CA|PIK3CB|PIK3CG|MAPK3|PIK3R5|MAPK1|PIK3R1|NFKB1|RELA|MAPK9|ERBB2|TGFBR2|TGFBR1|CCND1|CASP9	2.27E-17	4.85E-16	Metabolism of other amino acids	AKT1, BCL2L1, NFKB1, RELA, STAT3, TGFBR1, TGFBR2
EA	HIF-1 signaling pathway	ERBB2|IGF1R|IGF1|STAT3|AKT1|PIK3CA|PIK3CB|PRKCB|EGFR|INSR|calcium ions|IL6R|PLCG2|MAPK3|PIK3R5|MAPK1|PIK3R1|NFKB1|RELA|EP300|PIK3CG	8.58E-15	1.16E-13	Endocrine and metabolic diseases	AKT1, NFKB1, RELA, STAT3
EA	TNF signaling pathway	MAP3K8|CASP3|MAPK3|PIK3CA|PIK3CB|NFKBIA|JUN|CASP8|PIK3CG|MAPK14|AKT1|PIK3R5|MAPK1|MAPK10|MAPK11|PIK3R1|NFKB1|RELA|MAPK9|TNF|ATF2	1.18E-14	1.51E-13	Endocrine and metabolic diseases	AKT1, CASP3, JUN, NFKB1, RELA

EA	Estrogen signaling pathway	MMP9|PLCB1|NOS3|GNAI3|MAPK3|calcium ions|SP1|PIK3CB|FOS|JUN|PRKCD|SRC|PIK3CG|AKT1|PIK3R5|MAPK1|PIK3R1|PIK3CA|EGFR|HRAS|ATF2	4.64E-12	2.84E-11	Endocrine system	AKT1, FOS, JUN	
SB	Pathways in cancer	BCL2L1|PTGS2|NOS2|PRKCB|TP53|CASP3|calcium ions|FOS|JUN|TGFB1|CYCS|CASP9|AKT1|MAPK10|CASP8|cholesterol|SHH|MAPK8|MAPK9|MAPK3|BCL2	4.26E-10	2.19E-08	Signal transduction	AKT1, BCL2, BCL2L1, CASP3, CYCS, FOS, JUN, TP53	
EA	Colorectal cancer	MAPK9|PIK3R1|FOS|CCND1|PIK3CA|PIK3CB|JUN|CASP9|CTNNB1|AKT1|PIK3R5|MAPK1|MAPK10|GSK3B|PIK3CG|BCL2|MYC|SMAD3|MAPK3|CASP3	5.80E-18	1.36E-16	Amino acid metabolism	AKT1, BCL2, CASP3, FOS, GSK3B, JUN	
EA	ErbB signaling pathway	SRC|PTK2|MAPK3|PIK3CA|PIK3CB|EGFR|JUN|PIK3CG|PLCG2|AKT1|PIK3R5|MAPK1|MAPK10|GSK3B|PIK3R1|CDKN1A|CRK|MYC|MAPK9|HRAS	1.68E-12	1.14E-11	Amino acid metabolism	AKT1, GSK3B, JUN	
EA	Apoptosis	BCL2L1|TP53|CASP3|PIK3CA|PIK3CB|NFKBIA|NTRK1|CASP8|PIK3CG|AKT1|PIK3R5|TNF|APAF1|PIK3R1|NFKB1|IL1B|CASP9|RELA|IL1R1|BCL2	2.16E-12	1.41E-11	Lipid metabolism	AKT1, BCL2, BCL2L1, CASP3, NFKB1, RELA, TP53	APAF1
EA	B cell receptor signaling pathway	NFATC2|PRKCB|HRAS|FOS|SYK|PIK3CA|PIK3CB|NFKBIA|JUN|calcium ions|PIK3CG|PLCG2|AKT1|PIK3R5|MAPK1|PIK3R1|NFKB1|RELA|GSK3B|MAPK3	3.18E-14	3.40E-13	Lipid metabolism	AKT1, FOS, GSK3B, JUN, NFKB1, RELA	
EA	Non-alcoholic fatty liver disease (NAFLD)	PIK3CA|CASP3|PIK3CG|PIK3CB|JUN|IRS1|CASP8|IL6R|AKT1|INSR|TNF|MAPK10|GSK3B|PIK3R1|PIK3R5|NFKB1|IL1B|MAPK9|RELA|glucose	1.96E-13	1.63E-12	Lipid metabolism	AKT1, CASP3, GSK3B, JUN, NFKB1, RELA	
PCA	Pathways in cancer	PTGS2|NOS2|TP53|MAPK3|calcium ions|PIK3CA|PIK3CB|NFKB1|CASP8|PLCG2|AKT1|AKT3|VEGFA|cholesterol|MAPK1|RELA|BCL2|CDKN1A|DHEA|CASP3	3.49E-09	8.15E-08	Nervous system	AKT1, BCL2, CASP3, NFKB1, RELA, TP53	VEGFA
EA	Tuberculosis	TGFB1|MAPK3|STAT1|calcium ions|SYK|CASP8|CASP9|MAPK14|AKT1|CD14|MAPK1|MAPK10|MAPK11|BCL2|NFKB1|RELA|MAPK9|IL1B|TNF|CASP3	1.14E-07	4.49E-07	Signal transduction	AKT1, BCL2, CASP3, CD14, NFKB1, RELA	
CA	2-Oxocarboxylic acid metabolism	GOT1|oxaloacetate|glutamic acid|aspartic acid	0.00419974	0.0348172	Signal transduction		

CT	2-Oxocarboxylic acid metabolism	GOT1|glutamic acid|aspartic acid	0.005737087	0.077601655	Metabolism of other amino acids	
FA	2-Oxocarboxylic acid metabolism	GOT1|glutamic acid|aspartic acid	0.005737087	0.020399688	Lipid metabolism	
SA	2-Oxocarboxylic acid metabolism	GOT1|glutamic acid|aspartic acid	0.009217462	0.03948146	Infectious diseases	
TI	2-Oxocarboxylic acid metabolism	GOT1|glutamic acid|aspartic acid	0.008170374	0.104989309	Lipid metabolism	
EA	Acute myeloid leukemia	MYC|STAT3|MAPK3|CCND1|PIK3CA|HRAS|PIK3CG|AKT1|PIK3R5|MAPK1|PIK3R1|NFKB1|RELA|PIK3CB	4.56E-09	2.13E-08	Endocrine system	AKT1, NFKB1, RELA, STAT3
FA	Acute myeloid leukemia	AKT1|PIK3CB|MAPK1|RELA|MAPK3	0.000264675	0.001533306	Excretory system	AKT1, RELA
PCA	Acute myeloid leukemia	MAPK3|PIK3CA|PIK3CB|AKT1|AKT3|NFKB1|MAPK1|RELA	2.50E-06	1.53E-05	Digestive system	AKT1, NFKB1, RELA
TIIA	Acute myeloid leukemia	CCND1|MAPK3|MYC|MAPK1	0.009252758	0.039632648	Immune system	
EA	Adherens junction	ERBB2|SRC|IGF1R|TGFBR2|EGFR|TGFBR1|CTNNB1|MAPK3|MAPK1|ACTB|SNAI1|CSNK2A1|MET	1.55E-07	5.94E-07	Infectious diseases	CSNK2A1, MET, TGFBR1, TGFBR2
SA	Adherens junction	SRC|MAPK1|EGFR|MAPK3|PTPRF	0.000934671	0.007748727	Neurodegenerative diseases	
EA	Adipocytokine signaling pathway	PCK2|PCK1|STAT3|NFKBIA|IRS1|G6PC3|G6PC2|TNF|MAPK10|NFKB1|RELA|MAPK9	3.75E-07	1.40E-06	Neurodegenerative diseases	NFKB1, RELA, STAT3
SB	Adipocytokine signaling pathway	MAPK9|MAPK8|PCK1|MAPK10|TNF	0.001840609	0.007060246	Infectious diseases	

TIIA	Adipocytokine signaling pathway	PPARA|PCK1|TNF|G6PC2	0.009252758	0.039632648	Cellular community - eukaryotes		
EA	Adrenergic signaling in cardiomyocytes	PLCB1|MAPK3|calcium ions|PIK3CA|PIK3CB|PPP1CC|PIK3CG|MAPK14|AKT1|PIK3R5|MAPK1|MAPK11|PIK3R1|BCL2|GNAI3|ATF2	8.39E-05	0.000231867	Endocrine and metabolic diseases	AKT1, BCL2	
PCA	Adrenergic signaling in cardiomyocytes	calcium ions|CALML3|PIK3CA|PIK3CB|AKT1|AKT3|MAPK1|MAPK3|BCL2	0.000732075	0.002577304	Development	AKT1, BCL2	
CA	African trypanosomiasis	TNF|VCAM1|ICAM1|calcium ions	0.001422205	0.0158916	Circulatory system		ICAM1, VCAM1
CT	African trypanosomiasis	VCAM1|ICAM1|calcium ions	0.002508703	0.042982448	Amino acid metabolism		ICAM1, VCAM1
DA	African trypanosomiasis	VCAM1|ICAM1|calcium ions	0.001219928	0.014250979	Metabolism of cofactors and vitamins		ICAM1, VCAM1
EA	African trypanosomiasis	PLCB1|calcium ions|PRKCB|TNF|VCAM1|ICAM1	0.000254483	0.000660628	Infectious diseases		ICAM1, VCAM1
FA	African trypanosomiasis	IFNG|TNF|VCAM1|ICAM1|calcium ions	6.45E-06	9.76E-05	Infectious diseases		ICAM1, VCAM1
PCA	African trypanosomiasis	TNF|VCAM1|ICAM1|calcium ions	0.000775295	0.002692577	Signal transduction		ICAM1, VCAM1
SA	African trypanosomiasis	IFNG|TNF|VCAM1|ICAM1|calcium ions	1.51E-05	0.000533558	Xenobiotics biodegradation and metabolism		ICAM1, VCAM1
SB	African trypanosomiasis	PRKCB|TNF|VCAM1|ICAM1|calcium ions	5.13E-05	0.0003066	Signal transduction		ICAM1, VCAM1

TI	African trypanosomiasis	TNF|VCAM1|ICAM1|calcium ions	0.000240112	0.005142399	Cancers	ICAM1, VCAM1
TIIA	African trypanosomiasis	TNF|VCAM1|ICAM1|calcium ions	0.000572408	0.004903628	Sensory system	ICAM1, VCAM1
CA	Alanine, aspartate and glutamate metabolism	aspartic acid|ASNS|glutamic acid|oxaloacetate|GOT1|ASS1|GAD1|GAD2|CAD	1.46E-06	4.17E-05	Circulatory system	
CT	Alanine, aspartate and glutamate metabolism	aspartic acid|ASNS|glutamic acid|GOT1|ASS1|GAD2|CAD	2.33E-06	9.99E-05	Infectious diseases	
DA	Alanine, aspartate and glutamate metabolism	aspartic acid|GOT1|ASS1|GAD1|ASNS	0.000110887	0.002590721	Development	
EA	Alanine, aspartate and glutamate metabolism	GOT1|ALDH4A1|aspartic acid|L-delta 1-pyrroline-5-carboxylate|ASNS|glutamic acid|GLUL|GLS|ASS1|GAD1|GAD2|CAD	8.18E-07	2.88E-06	Infectious diseases	
FA	Alanine, aspartate and glutamate metabolism	aspartic acid|ASNS|glutamic acid|GOT1|ASS1|GAD1|GAD2|CAD	1.44E-07	1.23E-05	Metabolism of other amino acids	
PCA	Alanine, aspartate and glutamate metabolism	ALDH4A1|aspartic acid|L-delta 1-pyrroline-5-carboxylate|ASNS|glutamic acid|GOT1|ASS1|GAD1|GAD2|CAD	2.73E-08	3.35E-07	Nervous system	
SA	Alanine, aspartate and glutamate metabolism	aspartic acid|ASNS|glutamic acid|GOT1|ASS1|GAD2|CAD	7.49E-06	0.00039891	Sensory system	

SB	Alanine, aspartate and glutamate metabolism	GOT1|aspartic acid|ASNS|glutamic acid|GLUL|GLS|ASS1|GAD1|GAD2|CAD	2.45E-08	5.26E-07	Infectious diseases	
TI	Alanine, aspartate and glutamate metabolism	aspartic acid|ASNS|glutamic acid|GOT1|ASS1|GAD1|CAD	5.57E-06	0.000286153	Metabolism of other amino acids	
TIIA	Alanine, aspartate and glutamate metabolism	ALDH4A1|aspartic acid|L-delta 1-pyrroline-5-carboxylate|ASNS|glutamic acid|GOT1|ASS1|GAD1|GAD2|CAD	1.24E-08	1.07E-06	Endocrine and metabolic diseases	
EA	Aldosterone-regulated sodium reabsorption	IGF1|IRS1|PIK3CA|PIK3CB|PIK3R5|PIK3CG|INSR|PIK3R1	1.86E-05	5.50E-05	Lipid metabolism	
CA	alpha-Linolenic acid metabolism	PLA2G2A|PLA2G4A|PLA2G4F|PLA2G1B	0.004642452	0.037284692	Cancers	PLA2G2A
CT	alpha-Linolenic acid metabolism	PLA2G2A|PLA2G4F|PLA2G1B	0.0061983	0.079648156	Endocrine system	PLA2G2A
DA	alpha-Linolenic acid metabolism	PLA2G2A|PLA2G4A|PLA2G4F|PLA2G1B	0.000194096	0.003837134	Endocrine system	PLA2G2A
EA	alpha-Linolenic acid metabolism	PLA2G2A|PLA2G16|PLA2G6|PLA2G1B|PLA2G4B|PLA2G4A|PLA2G4F	0.000225541	0.00059147	Nervous system	PLA2G2A
FA	alpha-Linolenic acid metabolism	PLA2G2A|PLA2G4A|PLA2G4F|PLA2G1B	0.000510009	0.002621446	Signal transduction	PLA2G2A
PCA	alpha-Linolenic acid metabolism	PLA2G2A|PLA2G4A|PLA2G4F|PLA2G1B	0.002586751	0.008107256	Global and overview maps	PLA2G2A
SA	alpha-Linolenic acid metabolism	PLA2G2A|PLA2G4F|PLA2G1B	0.009944565	0.041897594	Metabolism of cofactors and vitamins	PLA2G2A

SB	alpha-Linolenic acid metabolism	PLA2G2A|PLA2G4A|PLA2G4F|PLA2G1B	0.002484385	0.009070988	Endocrine and metabolic diseases		PLA2G2A	
TIIA	alpha-Linolenic acid metabolism	PLA2G2A|PLA2G4A|PLA2G4F|PLA2G1B	0.001928247	0.013354747	Amino acid metabolism		PLA2G2A	
EA	Alzheimer's disease	PLCB1|CASP3|calcium ions|CASP8|CASP9|GSK3B|APAF1	0.001169251	0.002808388	Nervous system	CASP3, GSK3B		APAF1
PCA	Alzheimer's disease	CALML3|CASP3|CASP8|calcium ions	0.006679891	0.017882625	Metabolism of other amino acids	CASP3		
SB	Alzheimer's disease	CASP3|calcium ions|CALML3|CASP8|CASP9|CYCS	8.92E-05	0.000487747	Cancers	CASP3, CYCS		
TIIA	Alzheimer's disease	calcium ions|CASP3|APAF1|CASP9	0.005031697	0.024868193	Signal transduction	CASP3		APAF1
EA	Amoebiasis	PLCB1|PIK3CA|PIK3CB|PRKCB|PIK3CG|PIK3R5|PIK3R1|NFKB1|IL1B|RELA|IL1R1	2.31E-07	8.72E-07	Signal transduction	NFKB1, RELA		
PCA	Amoebiasis	PIK3CA|IL1B|RELA|PIK3CB|NFKB1	0.000654519	0.002369175	Nervous system	NFKB1, RELA		
EA	Amphetamine addiction	PPP1CC|calcium ions|PRKCB|JUN|glutamic acid|FOS|ATF2	0.007331751	0.015444755	Signal transduction	FOS, JUN		
SA	Amphetamine addiction	CALML3|SIRT1|glutamic acid|calcium ions	0.008187965	0.037586137	Nervous system	SIRT1		
SB	Amphetamine addiction	calcium ions|CALML3|PRKCB|JUN|glutamic acid|FOS	0.00052728	0.002377384	Cancers	FOS, JUN		
EA	Amyotrophic lateral sclerosis (ALS)	BCL2L1|GPX1|CASP3|glutamic acid|CASP9|MAPK14|TNF|MAPK11|APAF1|BCL2	1.23E-05	3.71E-05	Cellular community - eukaryotes	BCL2, BCL2L1, CASP3		APAF1
SA	Amyotrophic lateral sclerosis (ALS)	BCL2L1|TNF|CASP3|glutamic acid	0.004006388	0.023945159	Nervous system	BCL2L1, CASP3		
SB	Amyotrophic lateral sclerosis (ALS)	BCL2L1|CASP3|CYCS|CASP9|TNF|glutamic acid|BCL2	1.75E-05	0.00014092	Cancers	BCL2, BCL2L1, CASP3, CYCS		

TIIA	Amyotrophic lateral sclerosis (ALS)	CASP9|TNF|glutamic acid|APAF1|CASP3	0.001057029	0.007989894	Development	CASP3		APAF1
FA	Apoptosis	TP53|PIK3CB|NFKBIA|AKT1|TNF|RELA	0.000170989	0.00102196	Cancers	AKT1, RELA, TP53		
PCA	Apoptosis	TP53|CASP3|PIK3CA|PIK3CB|CASP8|AKT1|AKT3|NFKB1|IL1B|RELA|TNF|BCL2	3.88E-09	8.32E-08	Infectious diseases	AKT1, BCL2, CASP3, NFKB1, RELA, TP53		
SA	Apoptosis	BCL2L1|IL1B|TNF|TP53|CASP3	0.003036888	0.019036103	Neurodegenerative diseases	BCL2L1, CASP3, TP53		
SB	Apoptosis	BCL2L1|TP53|CASP3|CASP8|CASP9|AKT1|TNF|CYCS|IL1B|BCL2	4.45E-07	5.20E-06	Cancers	AKT1, BCL2, BCL2L1, CASP3, CYCS, TP53		
TIIA	Apoptosis	TNF|TP53|CASP3|APAF1|CASP9	0.006636495	0.031010532	Cancers	CASP3, TP53		APAF1
CA	Arachidonic acid metabolism	PTGS2|PTGS1|ALOX5|CYP2E1|PLA2G2A|ALOX15|GGT1|PLA2G1B|PLA2G4A|CYP4F3|PLA2G4F|arachidonic acid	2.18E-07	8.01E-06	Infectious diseases	CYP2E1	PLA2G2A	
CT	Arachidonic acid metabolism	PTGS2|PTGS1|CYP2E1|PLA2G2A|GGT1|PLA2G1B|CYP4F3|PLA2G4F|arachidonic acid	5.61E-07	2.88E-05	Infectious diseases	CYP2E1	PLA2G2A	
DA	Arachidonic acid metabolism	PTGS2|PTGS1|CYP2E1|PLA2G2A|PLA2G1B|PLA2G4A|CYP4F3|PLA2G4F|arachidonic acid	5.91E-08	1.52E-05	Infectious diseases	CYP2E1	PLA2G2A	
EA	Arachidonic acid metabolism	GPX1|PTGS2|PTGS1|PLA2G1B|PLA2G16|CYP2E1|PLA2G6|ALOX15|GGT1|GPX5|CYP2J2|PLA2G2A|PLA2G4B|PLA2G4A|CYP4F3|PLA2G4F|arachidonic acid	3.28E-08	1.43E-07	Metabolism of other amino acids	CYP2E1	PLA2G2A	
FA	Arachidonic acid metabolism	PTGS2|PTGS1|CYP2E1|PLA2G2A|PLA2G1B|PLA2G4A|CYP4F3|PLA2G4F|arachidonic acid	5.61E-07	1.92E-05	Carbohydrate metabolism	CYP2E1	PLA2G2A	
PCA	Arachidonic acid metabolism	PTGS2|PTGS1|CYP2E1|PLA2G2A|GGT1|PLA2G1B|PLA2G4A|CYP4F3|PLA2G4F|arachidonic acid	2.89E-06	1.73E-05	Endocrine system	CYP2E1	PLA2G2A	
SA	Arachidonic acid metabolism	PTGS2|PTGS1|CYP2E1|PLA2G2A|PLA2G1B|CYP4F3|PLA2G4F|arachidonic acid	2.25E-05	0.000576342	Infectious diseases	CYP2E1	PLA2G2A	

SB	Arachidonic acid metabolism	PTGS2|PTGS1|CYP2E1|PLA2G2A|ALOX15|GGT1|PLA2G1B|PLA2G4A|CYP4F3|PLA2G4F|arachidonic acid	2.99E-07	4.04E-06	Excretory system	CYP2E1	PLA2G2A
TI	Arachidonic acid metabolism	PTGS2|PTGS1|CYP2E1|GGT1|PLA2G1B|CYP4F3|PLA2G4F|arachidonic acid	1.62E-05	0.000595645	Endocrine system	CYP2E1	
TIIA	Arachidonic acid metabolism	CYP2C9|PTGS2|PTGS1|CYP2E1|PLA2G2A|GGT1|PLA2G1B|PLA2G4A|CYP4F3|PLA2G4F|arachidonic acid	1.45E-07	7.46E-06	Nervous system	CYP2E1	PLA2G2A
CA	Arginine and proline metabolism	3H-proline|LAP3|DAO|glutamic acid|PRODH|GATM|NOS3|ALDH7A1|PYCR2|GOT1|ASS1|aspartic acid	4.20E-07	1.35E-05	Infectious diseases		
CT	Arginine and proline metabolism	3H-proline|LAP3|glutamic acid|PRODH|GATM|NOS3|PYCR2|GOT1|ASS1|aspartic acid	7.86E-08	5.05E-06	Infectious diseases		
DA	Arginine and proline metabolism	3H-proline|LAP3|PRODH|NOS3|PYCR2|GOT1|ASS1|aspartic acid	1.37E-06	7.06E-05	Cancers		
EA	Arginine and proline metabolism	GLS|ALDH4A1|NOS3|LAP3|L-delta 1-pyrroline-5-carboxylate|DAO|ALDH2|PRODH|GATM|GLUL|3H-proline|glutamic acid|ALDH1B1|PYCR2|GOT1|ASS1|aspartic acid	8.12E-08	3.31E-07	Lipid metabolism		
FA	Arginine and proline metabolism	NOS3|LAP3|glutamic acid|PRODH|GATM|3H-proline|PYCR2|GOT1|ASS1|aspartic acid	7.86E-08	1.01E-05	Global and overview maps		
PCA	Arginine and proline metabolism	NOS2|3H-proline|ALDH4A1|LAP3|L-delta 1-pyrroline-5-carboxylate|DAO|glutamic acid|PRODH|GATM|NOS3|citrulline|PYCR2|GOT1|ASS1|aspartic acid	5.17E-11	5.09E-09	Carbohydrate metabolism		
SA	Arginine and proline metabolism	NOS2|3H-proline|LAP3|glutamic acid|PRODH|GATM|NOS3|citrulline|PYCR2|GOT1|ASS1|aspartic acid	3.15E-09	8.10E-07	Immune system		
SB	Arginine and proline metabolism	NOS2|GLS|LAP3|glutamic acid|PRODH|GATM|GLUL|3H-proline|NOS3|citrulline|PYCR2|GOT1|ASS1|aspartic acid	5.34E-10	2.23E-08	Infectious diseases		

TI	Arginine and proline metabolism	3H-proline|LAP3|glutamic acid|PRODH|GATM|NOS3|PYCR2|GOT1|ASS1|aspartic acid	2.75E-07	2.36E-05	Endocrine and metabolic diseases	
TIIA	Arginine and proline metabolism	3H-proline|ALDH4A1|LAP3|L-delta 1-pyrroline-5-carboxylate|glutamic acid|PRODH|GATM|NOS3|ALDH7A1|PYCR2|GOT1|ASS1|aspartic acid	2.48E-09	3.84E-07	Amino acid metabolism	
CA	Ascorbate and aldarate metabolism	UGT1A10|UGT1A8|UGT1A9|ALDH7A1|UGT1A7|UGT1A3	3.74E-05	0.000687191	Endocrine system	
DA	B cell receptor signaling pathway	AKT1|RELA|calcium ions|MAPK3	0.001891435	0.020254117	Cancers	AKT1, RELA
FA	B cell receptor signaling pathway	MAPK3|PIK3CB|NFKBIA|calcium ions|AKT1|MAPK1|RELA	4.43E-06	7.12E-05	Cancers	AKT1, RELA
PCA	B cell receptor signaling pathway	MAPK3|NFKBIB|SYK|PIK3CB|calcium ions|PLCG2|AKT1|AKT3|PIK3CA|NFKB1|MAPK1|RELA	3.41E-10	1.75E-08	Neurodegenerative diseases	AKT1, NFKB1, RELA
SB	B cell receptor signaling pathway	MAPK3|PRKCB|JUN|calcium ions|AKT1|FOS	0.000571955	0.002534351	Signal transduction	AKT1, FOS, JUN
EA	Bacterial invasion of epithelial cells	SRC|PTK2|PIK3CA|PIK3CB|PIK3CG|CTNNB1|MET|PIK3R5|PIK3R1|CRK|ACTB	1.11E-06	3.80E-06	Cancers	MET
CA	beta-Alanine metabolism	ALDH7A1|aspartic acid|beta-alanine|GAD2|GAD1	0.002401005	0.024682328	Cancers	
EA	beta-Alanine metabolism	ALDH2|GAD1|ALDH1B1|beta-alanine|GAD2|aspartic acid	0.007781868	0.016259676	Infectious diseases	
TIIA	beta-Alanine metabolism	ALDH7A1|aspartic acid|beta-alanine|GAD2|GAD1	0.000812449	0.006327253	Immune system	
CA	Biosynthesis of amino acids	3H-proline|glycine|glutamic acid|SHMT1|ALDH7A1|PYCR2|GOT1|ASS1|oxaloacetate|aspartic acid	0.000151354	0.002288121	Circulatory system	
CT	Biosynthesis of amino acids	3H-proline|glycine|glutamic acid|PYCR2|GOT1|ASS1|SHMT1|aspartic acid	5.11E-05	0.00145901	Immune system	

DA	Biosynthesis of amino acids	3H-proline|glycine|PYCR2|GOT1|ASS1|SHMT1|aspartic acid	7.20E-05	0.001909374	Endocrine system	
EA	Biosynthesis of amino acids	3H-proline|glycine|L-delta 1-pyrroline-5-carboxylate|glutamic acid|GLUL|PYCR2|GOT1|ASS1|SHMT2|SHMT1|aspartic acid	0.005691543	0.012395987	Signal transduction	
FA	Biosynthesis of amino acids	3H-proline|glycine|glutamic acid|PYCR2|GOT1|ASS1|SHMT1|aspartic acid	5.11E-05	0.000397912	Global and overview maps	
PCA	Biosynthesis of amino acids	3H-proline|glycine|L-delta 1-pyrroline-5-carboxylate|glutamic acid|citrulline|PYCR2|GOT1|ASS1|SHMT1|aspartic acid	3.78E-05	0.000179782	Development	
SA	Biosynthesis of amino acids	3H-proline|glycine|glutamic acid|citrulline|PYCR2|GOT1|ASS1|SHMT1|aspartic acid	2.65E-05	0.000576342	Endocrine and metabolic diseases	
SB	Biosynthesis of amino acids	3H-proline|glycine|glutamic acid|GLUL|citrulline|PYCR2|GOT1|ASS1|SHMT1|aspartic acid	3.43E-05	0.000231868	Cancers	
TI	Biosynthesis of amino acids	3H-proline|glycine|glutamic acid|PYCR2|GOT1|ASS1|SHMT1|aspartic acid	0.000128856	0.003446207	Signal transduction	
TIIA	Biosynthesis of amino acids	3H-proline|glycine|L-delta 1-pyrroline-5-carboxylate|glutamic acid|ALDH7A1|PYCR2|GOT1|ASS1|SHMT1|aspartic acid	1.86E-05	0.000298665	Immune system	
EA	Bladder cancer	ERBB2|TP53|HRAS|EGFR|MAPK3|MAPK1	0.000203731	0.000545404	Infectious diseases	TP53
FA	Bladder cancer	MAPK3|MAPK1|TP53	0.002246211	0.009310908	Infectious diseases	TP53
PCA	Bladder cancer	MAPK3|MAPK1|TP53	0.007629	0.019804576	Metabolism of other amino acids	TP53
SA	Bladder cancer	MAPK3|MAPK1|EGFR|TP53	0.00024457	0.002857023	Circulatory system	TP53
TIIA	Bladder cancer	MAPK3|MAPK1|EGFR|TP53	0.000493049	0.004482828	Cancers	TP53
FA	Butanoate metabolism	glutamic acid|GAD1|GAD2	0.008261417	0.027573821	Metabolism of other amino acids	
EA	cAMP signaling pathway	MAPK3|calcium ions|PIK3CA|PIK3CB|FOS|JUN|PIK3CG|AKT1|PIK3R5|MAPK1|MAPK10|PIK3R1|NFKB1|RELA|MAPK9|GNAI3|PPP1CC	0.001468361	0.003462098	Xenobiotics biodegradation and metabolism	AKT1, FOS, JUN, NFKB1, RELA

PCA	cAMP signaling pathway	calcium ions|CALML3|PIK3CA|PIK3CB|AKT1|AKT3|NFKB1|MAPK1|RELA|MAPK3	0.001750191	0.005731858	Nervous system	AKT1, NFKB1, RELA
SB	cAMP signaling pathway	FOS|CALML3|calcium ions|JUN|AKT1|MAPK10|MAPK8|MAPK9|MAPK3	0.005565755	0.017028559	Endocrine system	AKT1, FOS, JUN
EA	Carbohydrate digestion and absorption	PIK3CA|PIK3CB|PRKCB|PIK3CG|AKT1|PIK3R5|PIK3R1	7.68E-07	2.74E-06	Nervous system	AKT1
PCA	Carbohydrate digestion and absorption	PIK3CA|PIK3CB|AKT3|AKT1	0.000118647	0.000516817	Infectious diseases	AKT1
EA	Cell cycle	PCNA|TGFB1|TP53|CCND1|CDKN1A|SMAD3|GSK3B|MYC|EP300	0.000213824	0.000566522	Infectious diseases	GSK3B, PCNA, TP53
EA	cGMP-PKG signaling pathway	PLCB1|NFATC2|NOS3|MAPK3|calcium ions|PIK3CA|PIK3CB|PRKCE|IRS1|INSR|PIK3CG|AKT1|PIK3R5|MAPK1|GNA12|PIK3R1|ATF2|GNAI3|PPP1CC	6.56E-06	2.03E-05	Cancers	AKT1
FA	cGMP-PKG signaling pathway	NOS3|calcium ions|PIK3CB|AKT1|MAPK1|MAPK3	0.008122299	0.027466194	Signal transduction	AKT1
PCA	cGMP-PKG signaling pathway	NOS3|calcium ions|CALML3|PIK3CA|PIK3CB|AKT1|AKT3|MAPK1|MAPK3	0.001526567	0.005162208	Lipid metabolism	AKT1
FA	Chagas disease (American trypanosomiasis)	MAPK3|IFNG|PIK3CB|NFKBIA|AKT1|MAPK1|RELA|TNF	1.94E-06	3.84E-05	Infectious diseases	AKT1, RELA
PCA	Chagas disease (American trypanosomiasis)	NOS2|MAPK3|PIK3CA|PIK3CB|CASP8|AKT1|AKT3|NFKB1|RELA|TNF|MAPK1	6.79E-08	6.94E-07	Lipid metabolism	AKT1, NFKB1, RELA

SA	Chagas disease (American trypanosomiasis)	IFNG|MAPK3|NOS2|MAPK1|TNF	0.003549898	0.021721996	Nervous system	
SB	Chagas disease (American trypanosomiasis)	TGFB1|NOS2|AKT1|FOS|JUN|CASP8|MAPK3|TNF|MAPK10|TLR4|MAPK8|MAPK9	5.21E-09	1.34E-07	Immune system	AKT1, FOS, JUN
CA	Chemical carcinogenesis	CYP1A1|CYP1A2|GSTT2|UGT1A10|chloroform/methanol|GSTM1|GSTM2|CYP2E1|trichloroethylene|GSTP1|UGT1A8|UGT1A9|UGT1A7|UGT1A3	1.60E-10	2.06E-08	Endocrine system	CYP2E1, MET
CT	Chemical carcinogenesis	CYP1A1|CYP3A4|CYP1A2|CYP2E1	0.009199198	0.10279104	Amino acid metabolism	CYP2E1
EA	Chemical carcinogenesis	CYP1A1|CYP1A2|CYP1B1|GSTA4|GSTM1|CYP2E1|GSTP1|BP-7,8-diol|CYP3A4	0.001772532	0.004103969	Infectious diseases	CYP1B1, CYP2E1
TI	Chemical carcinogenesis	GSTP1|CYP1A1|CYP3A4|CYP1A2|CYP2E1	0.002373463	0.040665324	Infectious diseases	CYP2E1
FA	Chemokine signaling pathway	MAPK3|PIK3CB|NFKBIA|calcium ions|AKT1|MAPK1|RELA	0.002865128	0.011156635	Cancers	AKT1, RELA
PCA	Chemokine signaling pathway	calcium ions|NFKBIB|PIK3CA|PIK3CB|AKT1|AKT3|NFKB1|MAPK1|RELA|MAPK3	0.000698407	0.002492925	Infectious diseases	AKT1, NFKB1, RELA
CA	Choline metabolism in cancer	EGFR|HIF1A|MAPK10|PIK3R1|PLA2G4A|PLA2G4F	0.003989451	0.0348172	Endocrine system	
DA	Choline metabolism in cancer	AKT1|PLA2G4A|PLA2G4F|MAPK3	0.003956044	0.03389011	Infectious diseases	AKT1
EA	Choline metabolism in cancer	PDGFRB|AKT1|FOS|SP1|HRAS|EGFR|JUN|MAPK9|PIK3CG|MAPK3|PIK3R5|MAPK1|MAPK10|PIK3R1|PIK3CA|PLA2G4B|PLA2G4A|PLA2G4F|PIK3CB	2.11E-11	1.18E-10	Development	AKT1, FOS, JUN
FA	Choline metabolism in cancer	MAPK3|PIK3CB|AKT1|MAPK1|PLA2G4A|PLA2G4F	0.000170989	0.00102196	Immune system	AKT1

PCA	Choline metabolism in cancer	AKT1|PIK3CA|PIK3CB|MAPK3|AKT3|MAPK1|PLA2G4A|PLA2G4F	3.90E-05	0.000182407	Immune system	AKT1
SB	Choline metabolism in cancer	FOS|JUN|PLA2G4F|MAPK3|AKT1|MAPK10|MAPK8|MAPK9|PLA2G4A	4.27E-06	4.22E-05	Cancers	AKT1, FOS, JUN
TIIA	Choline metabolism in cancer	MAPK3|PLA2G4A|MAPK1|EGFR|PLA2G4F	0.006636495	0.031010532	Transport and catabolism	
EA	Cholinergic synapse	PLCB1|PRKCB|GNAI3|MAPK3|calcium ions|PIK3CA|PIK3CB|FOS|PIK3CG|AKT1|PIK3R5|MAPK1|PIK3R1|HRAS|BCL2	1.34E-06	4.47E-06	Signal transduction	AKT1, BCL2, FOS
FA	Cholinergic synapse	AKT1|PIK3CB|MAPK1|calcium ions|MAPK3	0.0034035	0.013055218	Global and overview maps	AKT1
PCA	Cholinergic synapse	MAPK3|PIK3CA|PIK3CB|calcium ions|AKT1|AKT3|MAPK1|BCL2	0.000161625	0.000659329	Immune system	AKT1, BCL2
SB	Cholinergic synapse	FOS|PRKCB|calcium ions|AKT1|MAPK3|BCL2	0.004529795	0.014914112	Global and overview maps	AKT1, BCL2, FOS
DA	Chronic myeloid leukemia	AKT1|RELA|TP53|MAPK3	0.000841729	0.012018019	Carbohydrate metabolism	AKT1, RELA, TP53
EA	Chronic myeloid leukemia	BCL2L1|MYC|TP53|MAPK3|PIK3CA|HRAS|NFKBIA|SMAD3|AKT1|PIK3R5|MAPK1|PIK3R1|CDKN1A|NFKB1|RELA|PIK3CG|PIK3CB	1.11E-12	7.90E-12	Amino acid metabolism	AKT1, BCL2L1, NFKB1, RELA, TP53
FA	Chronic myeloid leukemia	TP53|MAPK3|PIK3CB|NFKBIA|AKT1|MAPK1|RELA	1.00E-06	2.16E-05	Infectious diseases	AKT1, RELA, TP53
PCA	Chronic myeloid leukemia	TP53|MAPK3|PIK3CA|PIK3CB|AKT1|AKT3|NFKB1|MAPK1|RELA|CDKN1A	7.94E-09	1.46E-07	Signal transduction	AKT1, NFKB1, RELA, TP53
SA	Chronic myeloid leukemia	BCL2L1|MAPK3|MAPK1|TP53|SHC1	0.000457612	0.004355792	Infectious diseases	BCL2L1, TP53

SB	Chronic myeloid leukemia	BCL2L1|AKT1|TP53|MAPK3	0.009728804	0.027475854	Endocrine and metabolic diseases	AKT1, BCL2L1, TP53
TIIA	Chronic myeloid leukemia	MAPK3|MYC|MAPK1|TP53	0.007655883	0.034518632	Cancers	TP53
FA	Colorectal cancer	AKT1|PIK3CB|MAPK1|MAPK3	0.001176606	0.005213582	Signal transduction	AKT1
PCA	Colorectal cancer	CASP3|MAPK3|PIK3CA|PIK3CB|AKT1|AKT3|MAPK1|BCL2	4.55E-07	3.65E-06	Neurodegenerative diseases	AKT1, BCL2, CASP3
SB	Colorectal cancer	CASP3|MAPK3|FOS|JUN|CASP9|AKT1|MAPK10|MAPK8|MAPK9|BCL2	1.34E-09	4.32E-08	Immune system	AKT1, BCL2, CASP3, FOS, JUN
TIIA	Colorectal cancer	CASP3|CCND1|CASP9|MAPK3|MAPK1|MYC	4.72E-05	0.000639052	Infectious diseases	CASP3
CA	Cyanoamino acid metabolism	GGT1|SHMT1|glycine	0.007427024	0.051587707	Endocrine and metabolic diseases	
CT	Cyanoamino acid metabolism	GGT1|SHMT1|glycine	0.0013697	0.029334403	Cancers	
EA	Cyanoamino acid metabolism	GGT1|SHMT1|SHMT2|glycine	0.00620371	0.013286278	Nervous system	
PCA	Cyanoamino acid metabolism	GGT1|SHMT1|glycine	0.004729081	0.013355757	Excretory system	
SB	Cyanoamino acid metabolism	GGT1|SHMT1|glycine	0.004584494	0.014914112	Global and overview maps	
TI	Cyanoamino acid metabolism	GGT1|SHMT1|glycine	0.001978822	0.036325519	Lipid metabolism	

TIIA	Cyanoamino acid metabolism	GGT1|SHMT1|glycine	0.003773882	0.021217319	Endocrine and metabolic diseases	
PCA	Cytosolic DNA-sensing pathway	NFKBIB|NFKB1|RELA	0.004729081	0.013355757	Nervous system	NFKB1, RELA
SB	D-Glutamine and D-glutamate metabolism	GLS|glutamic acid	0.006363641	0.01920064	Lipid metabolism	
EA	Dopaminergic synapse	PLCB1|PRKCB|PPP1CC|FOS|calcium ions|GSK3B|MAPK14|AKT1|MAPK10|MAPK11|MAPK9|GNAI3|ATF2	0.000269466	0.000692526	Infectious diseases	AKT1, FOS, GSK3B
SB	Dopaminergic synapse	PRKCB|FOS|CALML3|calcium ions|AKT1|MAPK10|MAPK8|MAPK9	0.000584901	0.002547788	Signal transduction	AKT1, FOS
EA	Dorso_ventral axis formation	MAPK3|MAPK1|EGFR	0.007098134	0.015076202	Global and overview maps	
FA	Dorso_ventral axis formation	MAPK3|MAPK1	0.006375811	0.021908225	Amino acid metabolism	
SA	Dorso_ventral axis formation	MAPK3|MAPK1|EGFR	0.000349827	0.003457907	Sensory system	
TIIA	Dorso_ventral axis formation	MAPK3|MAPK1|EGFR	0.000598968	0.004965635	Amino acid metabolism	
CA	Drug metabolism	CYP1A2|GSTT2|UGT1A10|GSTM1|GSTM2|CYP2E1|GSTP1|UGT1A8|UGT1A9|UGT1A7|UGT1A3	1.47E-05	0.000315548	Immune system	CYP2E1
EA	Endocytosis	TGFBR2|IGF1R|SRC|EGFR|NTRK1|SMAD3|MET|TGFBR1|KDR	0.001020018	0.002473063	Endocrine system	MET, TGFBR1, TGFBR2
EA	Endometrial cancer	CTNNB1|MAPK3|CCND1|PIK3CA|HRAS|EGFR|PIK3CG|FOXO3|AKT1|PIK3R5|MAPK1|PIK3R1|MYC|CASP9|PIK3CB	8.57E-13	6.29E-12	Carbohydrate metabolism	AKT1
FA	Endometrial cancer	AKT1|PIK3CB|MAPK1|MAPK3	0.00062554	0.003033278	Signal transduction	AKT1
PCA	Endometrial cancer	MAPK3|PIK3CA|PIK3CB|AKT1|AKT3|MAPK1	2.83E-05	0.000139774	Nervous system	AKT1
TIIA	Endometrial cancer	CCND1|EGFR|CASP9|FOXO3|MAPK3|MAPK1|MYC	1.15E-06	3.70E-05	Cancers	

EA	Epithelial cell signaling in Helicobacter pylori infection	SRC|JUN|NFKBIA|EGFR|MAPK14|MAPK10|MAPK11|CXCL8|NFKB1|RELA|MAPK9	1.79E-08	8.09E-08	Signal transduction	JUN, NFKB1, RELA
SB	Epithelial cell signaling in Helicobacter pylori infection	JUN|MAPK8|MAPK9|MAPK10	0.002242278	0.008474493	Immune system	JUN
FA	Epstein-Barr virus infection	AKT1|PIK3CB|NFKBIA|TP53|RELA	0.001498268	0.006417582	Cell growth and death	AKT1, RELA, TP53
PCA	Epstein-Barr virus infection	TP53|SYK|PLCG2|NFKBIB|PIK3CA|PIK3CB|AKT1|AKT3|NFKB1|RELA|CDKN1A	5.27E-08	5.65E-07	Cancers	AKT1, NFKB1, RELA, TP53
SB	Epstein-Barr virus infection	TP53|JUN|AKT1|MAPK10|MAPK8|MAPK9	0.001771912	0.006899719	Cancers	AKT1, JUN, TP53
FA	ErbB signaling pathway	AKT1|PIK3CB|MAPK1|MAPK3	0.009199198	0.030310178	Cancers	AKT1
PCA	ErbB signaling pathway	MAPK3|PIK3CA|PIK3CB|PLCG2|AKT1|AKT3|MAPK1|CDKN1A	3.57E-05	0.000173039	Infectious diseases	AKT1
SA	ErbB signaling pathway	MAPK3|MAPK1|EGFR|SHC1|SRC	0.0028785	0.018494365	Infectious diseases	
SB	ErbB signaling pathway	MAPK3|JUN|AKT1|MAPK10|MAPK8|MAPK9	0.001561975	0.006272307	Signal transduction	AKT1, JUN
CA	Estrogen signaling pathway	NOS3|estradiol|calcium ions|EGFR|PIK3R1|GNAI2	0.008159093	0.055181236	Signal transduction	
DA	Estrogen signaling pathway	AKT1|NOS3|calcium ions|MAPK3	0.006677917	0.046384454	Immune system	AKT1
FA	Estrogen signaling pathway	NOS3|calcium ions|PIK3CB|AKT1|MAPK1|MAPK3	0.00038293	0.002093891	Infectious diseases	AKT1
PCA	Estrogen signaling pathway	NOS3|calcium ions|CALML3|PIK3CA|PIK3CB|AKT1|AKT3|MAPK1|MAPK3	1.59E-05	8.33E-05	Amino acid metabolism	AKT1
SA	Estrogen signaling pathway	SRC|NOS3|SHC1|calcium ions|CALML3|EGFR|MAPK3|MAPK1	1.66E-05	0.000533558	Lipid metabolism	

SB	Estrogen signaling pathway	NOS3|FOS|CALML3|calcium ions|JUN|PRKCD|AKT1|MAPK3	0.000104092	0.000557328	Amino acid metabolism	AKT1, FOS, JUN
DA	Fc epsilon RI signaling pathway	AKT1|PLA2G4A|PLA2G4F|MAPK3	0.001103238	0.014176612	Lipid metabolism	AKT1
EA	Fc epsilon RI signaling pathway	PLA2G4A|MAPK3|SYK|PLA2G4F|HRAS|PIK3CG|MAPK14|PLCG2|PIK3R5|MAPK1|MAPK10|MAPK11|PIK3R1|PIK3CA|PLA2G4B|AKT1|MAPK9|PIK3CB	3.06E-13	2.39E-12	Neurodegenerative diseases	AKT1
FA	Fc epsilon RI signaling pathway	AKT1|PIK3CB|MAPK3|MAPK1|PLA2G4A|PLA2G4F	2.39E-05	0.000267053	Metabolism of other amino acids	AKT1
PCA	Fc epsilon RI signaling pathway	MAPK3|SYK|PIK3CA|PIK3CB|PLCG2|AKT3|AKT1|MAPK1|PLA2G4A|PLA2G4F	1.64E-08	2.23E-07	Metabolism of other amino acids	AKT1
SB	Fc epsilon RI signaling pathway	PLA2G4A|AKT1|MAPK3|MAPK10|MAPK8|MAPK9|PLA2G4F	2.83E-05	0.000196694	Immune system	AKT1
TIIA	Fc epsilon RI signaling pathway	MAPK3|PLA2G4A|MAPK1|PLA2G4F	0.009829753	0.040745912	Cell growth and death	
DA	Fc gamma R-mediated phagocytosis	AKT1|PLA2G4A|calcium ions|PLA2G4F|MAPK3	0.00068951	0.01042377	Substance dependence	AKT1
FA	Fc gamma R-mediated phagocytosis	MAPK3|PIK3CB|calcium ions|AKT1|MAPK1|PLA2G4A|PLA2G4F	3.17E-05	0.000313594	Signal transduction	AKT1
PCA	Fc gamma R-mediated phagocytosis	calcium ions|SYK|PIK3CA|PIK3CB|FCGR2A|PLCG2|AKT1|AKT3|FCGR1A|MAPK1|PLA2G4A|PLA2G4F|MAPK3	9.74E-10	3.13E-08	Cardiovascular diseases	AKT1
SB	Fc gamma R-mediated phagocytosis	NCF1|MAPK3|PRKCB|PRKCD|calcium ions|AKT1|PLA2G4A|PLA2G4F	7.07E-05	0.00040403	Development	AKT1
TIIA	Fc gamma R-mediated phagocytosis	MAPK3|PLA2G4A|MAPK1|PLA2G4F|calcium ions	0.009765739	0.040745912	Signal transduction	

PCA	Focal adhesion	MAPK3|PIK3CA|PIK3CB|AKT1|AKT3|VEGFA|MAPK1|BCL2	0.001622427	0.005415112	Infectious diseases	AKT1, BCL2	VEGFA
SB	Focal adhesion	MAPK3|PRKCB|JUN|AKT1|MAPK10|MAPK8|MAPK9|BCL2	0.001510453	0.006161688	Signal transduction	AKT1, BCL2, JUN	
CA	FoxO signaling pathway	PCK2|EGFR|G6PC3|FOXO3|CDK2|MAPK10|glutamic acid|PIK3R1|FOXO4|FOXO6	0.000117047	0.002005409	Global and overview maps	CDK2	
FA	FoxO signaling pathway	AKT1|PIK3CB|MAPK1|glutamic acid|MAPK3	0.009623944	0.030916919	Infectious diseases	AKT1	
PCA	FoxO signaling pathway	MAPK3|PIK3CA|PIK3CB|glutamic acid|AKT1|AKT3|MAPK1|CDKN1A	0.000866064	0.002967711	Infectious diseases	AKT1	
SB	FoxO signaling pathway	TGFB1|TGFBR2|TGFBR1|PCK1|MAPK3|glutamic acid|AKT1|MAPK10|MAPK8|MAPK9	2.62E-05	0.000187254	Infectious diseases	AKT1, TGFBR1, TGFBR2	
TIIA	FoxO signaling pathway	PCK1|CCND1|EGFR|glutamic acid|G6PC2|FOXO3|MAPK3|MAPK1	0.000505844	0.004482828	Signal transduction		
CT	Galactose metabolism	GLA|GCK|GANC|SI|sucrose|glucose	2.39E-05	0.000767777	Endocrine system		
DA	Galactose metabolism	GLA|GANC|SI|glucose	0.001103238	0.014176612	Metabolism of other amino acids		
FA	Galactose metabolism	GLA|GANC|SI|glucose	0.002792319	0.011156635	Cancers		
PCA	Galactose metabolism	GLA|GCK|GANC|SI|sucrose|glucose	0.000275768	0.001057796	Amino acid metabolism		
TI	Galactose metabolism	GLA|GCK|GANC|SI|sucrose|glucose	4.97E-05	0.001598043	Cancers		
TIIA	Galactose metabolism	GLA|GANC|glucose|SI|G6PC2	0.001463327	0.010745	Amino acid metabolism		
EA	Gap junction	PLCB1|PRKCB|PDGFRB|HRAS|calcium ions|EGFR|SRC|MAPK3|MAPK1|GNAI3	0.001283837	0.003055055	Cell growth and death		
SA	Gap junction	MAPK3|MAPK1|EGFR|calcium ions|SRC	0.005219698	0.027376782	Infectious diseases		
DA	Glioma	AKT1|calcium ions|TP53|MAPK3	0.001504508	0.016811242	Amino acid metabolism	AKT1, TP53	
EA	Glioma	PDGFRB|IGF1R|IGF1|HRAS|calcium ions|PIK3CA|PIK3CB|PRKCB|EGFR|PIK3CG|PLCG2|AKT1|PIK3R5|MAPK1|PIK3R1|CDKN1A|TP53|MAPK3	1.51E-12	1.05E-11	Immune system	AKT1, TP53	

FA	Glioma	TP53|calcium ions|PIK3CB|AKT1|MAPK1|MAPK3	3.86E-05	0.00033401	Endocrine and metabolic diseases	AKT1, TP53
PCA	Glioma	TP53|MAPK3|CALML3|PIK3CA|PIK3CB|calcium ions|PLCG2|AKT1|AKT3|MAPK1|CDKN1A	2.60E-09	6.69E-08	Lipid metabolism	AKT1, TP53
SA	Glioma	TP53|SHC1|calcium ions|CALML3|EGFR|MAPK3|MAPK1	9.31E-06	0.00039891	Immune system	TP53
SB	Glioma	TP53|MAPK3|CALML3|PRKCB|calcium ions|AKT1	0.000409368	0.001912863	Endocrine system	AKT1, TP53
TIIA	Glioma	MAPK3|MAPK1|EGFR|TP53|calcium ions	0.002120614	0.013624943	Signal transduction	TP53
DA	Glutamatergic synapse	MAPK3|PLA2G4A|PLA2G4F|calcium ions	0.009155672	0.06033353	Lipid metabolism	
EA	Glutamatergic synapse	PLCB1|calcium ions|PRKCB|glutamic acid|GNAI3|MAPK3|MAPK1|PLA2G4B|PLA2G4A|PLA2G4F	0.00298507	0.006670982	Lipid metabolism	
FA	Glutamatergic synapse	calcium ions|glutamic acid|MAPK3|MAPK1|PLA2G4A|PLA2G4F	0.000622645	0.003033278	Signal transduction	
PCA	Glutamatergic synapse	calcium ions|glutamic acid|MAPK3|MAPK1|PLA2G4A|PLA2G4F	0.005789846	0.015829686	Lipid metabolism	
SA	Glutamatergic synapse	MAPK3|MAPK1|PLA2G4F|glutamic acid|calcium ions	0.008336225	0.037586137	Immune system	
SB	Glutamatergic synapse	calcium ions|PRKCB|glutamic acid|MAPK3|PLA2G4A|PLA2G4F	0.005486834	0.016989352	Immune system	
TIIA	Glutamatergic synapse	calcium ions|glutamic acid|MAPK3|MAPK1|PLA2G4A|PLA2G4F	0.003906978	0.021217319	Infectious diseases	
CA	Glutathione metabolism	GSS|GSTT2|glycine|GSTM1|LAP3|reduced glutathione|glutamic acid|GCLC|GSTP1|GGT1|GSTM2|pyroglutamate|gamma-glutamylcysteine|GGCT|OPLAH	9.87E-13	2.54E-10	Infectious diseases	
CT	Glutathione metabolism	glycine|LAP3|GCLC|glutamic acid|GGCT|GGT1|pyroglutamate|gamma-glutamylcysteine|OPLAH	2.33E-08	2.44E-06	Infectious diseases	
DA	Glutathione metabolism	glycine|LAP3|GCLC|GGCT|pyroglutamate|gamma-glutamylcysteine|OPLAH	9.61E-07	6.17E-05	Carbohydrate metabolism	
EA	Glutathione metabolism	GSS|glycine|GSTA4|GSTM1|LAP3|GCLC|glutamic acid|reduced glutathione|GPX1|GSTP1|GGT1|GPX5|pyroglutamate|gamma-glutamylcysteine|GGCT|OPLAH	8.63E-10	4.19E-09	Lipid metabolism	

FA	Glutathione metabolism	glycine|LAP3|GCLC|glutamic acid|GGCT|pyroglutamate|gamma-glutamylcysteine|OPLAH	3.83E-07	1.92E-05	Nervous system
PCA	Glutathione metabolism	GSS|glycine|GSTM1|LAP3|reduced glutathione|glutamic acid|GCLC|GGT1|pyroglutamate|gamma-glutamylcysteine|GGCT|OPLAH	4.88E-10	2.09E-08	Amino acid metabolism
SA	Glutathione metabolism	glycine|LAP3|GCLC|glutamic acid|GGCT|ANPEP|gamma-glutamylcysteine|OPLAH|pyroglutamate	1.09E-07	1.39E-05	Endocrine system
SB	Glutathione metabolism	GSS|GSTT2|glycine|GSTM1|LAP3|reduced glutathione|glutamic acid|GCLC|GGT1|pyroglutamate|gamma-glutamylcysteine|GGCT|OPLAH	2.59E-11	3.33E-09	Global and overview maps
TI	Glutathione metabolism	glycine|reduced glutathione|LAP3|GCLC|glutamic acid|GGCT|GSTP1|GGT1|pyroglutamate|gamma-glutamylcysteine|OPLAH	2.43E-10	6.23E-08	Nervous system
TIIA	Glutathione metabolism	glycine|LAP3|reduced glutathione|glutamic acid|GCLC|GSTP1|GGT1|pyroglutamate|gamma-glutamylcysteine|GGCT|OPLAH	2.99E-09	3.84E-07	Nervous system
CA	Glycine, serine and threonine metabolism	GNMT|glycine|DAO|GATM|ALAS2|ALDH7A1|GCAT|SHMT1	2.57E-05	0.00050799	Cancers
CT	Glycine, serine and threonine metabolism	GCAT|SHMT1|glycine|GATM	0.004466777	0.063775655	Metabolism of other amino acids
EA	Glycine, serine and threonine metabolism	GNMT|glycine|DAO|GATM|ALAS2|GCAT|SHMT2|SHMT1	0.001815293	0.004165449	Lipid metabolism
FA	Glycine, serine and threonine metabolism	GCAT|SHMT1|glycine|GATM	0.004466777	0.016637127	Immune system
PCA	Glycine, serine and threonine metabolism	GATM|DAO|SHMT1|glycine|GCAT	0.003694267	0.011039844	Nervous system
SA	Glycine, serine and threonine metabolism	GATM|SHMT1|glycine|GCAT	0.008192853	0.037586137	Development
SB	Glycine, serine and threonine metabolism	GATM|GNMT|SHMT1|glycine|GCAT	0.003522054	0.012068904	Immune system

TI	Glycine, serine and threonine metabolism	GATM|SHMT1|glycine|GCAT	0.007026139	0.095037779	Cancers	
TIIA	Glycine, serine and threonine metabolism	GNMT|glycine|GATM|ALAS2|ALDH7A1|GCAT|SHMT1	4.25E-05	0.000639052	Cancers	
SB	Glyoxylate and dicarboxylate metabolism	GLUL|glutamic acid|SHMT1|glycine	0.009114982	0.026028338	Cancers	
DA	GnRH signaling pathway	MAPK3|PLA2G4A|PLA2G4F|calcium ions	0.005093925	0.040910581	Global and overview maps	
EA	GnRH signaling pathway	PLCB1|PRKCB|calcium ions|EGFR|HRAS|MAP3K1|JUN|PRKCD|SRC|MAPK14|MAPK3|PTK2B|MAPK1|MAPK10|MAPK11|PLA2G4B|PLA2G4A|MAPK9|PLA2G4F	7.95E-11	4.08E-10	Cancers	JUN
FA	GnRH signaling pathway	MAPK3|PLA2G4A|MAPK1|PLA2G4F|calcium ions	0.001944364	0.008191828	Cell growth and death	
PCA	GnRH signaling pathway	calcium ions|CALML3|MAPK3|MAPK1|PLA2G4A|PLA2G4F	0.002528399	0.008022204	Cancers	
SA	GnRH signaling pathway	SRC|calcium ions|CALML3|EGFR|MAPK3|MAPK1|PLA2G4F	8.40E-05	0.001439408	Endocrine and metabolic diseases	
SB	GnRH signaling pathway	calcium ions|CALML3|PRKCB|JUN|PRKCD|PLA2G4F|MAPK3|MAPK10|MAPK8|MAPK9|PLA2G4A	8.73E-08	1.73E-06	Nervous system	JUN
TIIA	GnRH signaling pathway	calcium ions|EGFR|MAPK3|MAPK1|PLA2G4A|PLA2G4F	0.001678776	0.011984594	Infectious diseases	
FA	Hepatitis B	TP53|MAPK3|PIK3CB|NFKBIA|AKT1|MAPK1|RELA|TNF	3.87E-05	0.00033401	Nervous system	AKT1, RELA, TP53
PCA	Hepatitis B	TP53|CASP3|MAPK3|PIK3CA|PIK3CB|AKT1|AKT3|NFKB1|MAPK1|RELA|CDKN1A|TNF|BCL2	7.02E-08	6.94E-07	Signal transduction	AKT1, BCL2, CASP3, NFKB1, RELA, TP53

SA	Hepatitis B	SRC|TP53|CASP3|MAPK3|MAPK1|TNF	0.004227502	0.024692456	Endocrine and metabolic diseases	CASP3, TP53	
SB	Hepatitis B	TGFB1|TGFBR1|TP53|CASP3|FOS|PRKCB|JUN|CYCS|CASP9|AKT1|TNF|MAPK10|TLR4|MAPK8|MAPK9|MAPK3|BCL2	5.65E-12	1.45E-09	Global and overview maps	AKT1, BCL2, CASP3, CYCS, FOS, JUN, TGFBR1, TP53	
TIIA	Hepatitis B	TP53|CASP3|CASP9|MAPK3|TNF|MAPK1|MYC|APAF1	0.000479855	0.004482828	Endocrine system	CASP3, TP53	APAF1
DA	Hepatitis C	AKT1|RELA|TP53|MAPK3	0.002593871	0.024689805	Signal transduction	AKT1, RELA, TP53	
FA	Hepatitis C	TP53|MAPK3|PIK3CB|NFKBIA|AKT1|MAPK1|RELA|TNF	5.96E-07	1.92E-05	Nervous system	AKT1, RELA, TP53	
PCA	Hepatitis C	TP53|MAPK3|PIK3CA|PIK3CB|AKT1|AKT3|TNF|NFKB1|RELA|CDKN1A|MAPK1	1.32E-08	1.89E-07	Cancers	AKT1, NFKB1, RELA, TP53	
SA	Hepatitis C	MAPK3|MAPK1|EGFR|TP53|TNF	0.001818659	0.012983203	Neurodegenerative diseases	TP53	
SB	Hepatitis C	TP53|MAPK3|AKT1|TNF|MAPK10|MAPK8|MAPK9	0.000126917	0.000639565	Cell growth and death	AKT1, TP53	
TIIA	Hepatitis C	MAPK3|MAPK1|EGFR|TP53|TNF	0.004045326	0.021217319	Signal transduction	TP53	
CA	Herpes simplex infection	CASP3|HCFC1|CDK2|TNF|MAPK10|CSNK2A2	0.009005203	0.05934198	Endocrine system	CASP3, CDK2	
EA	Herpes simplex infection	CASP3|STAT1|FOS|IRF9|NFKBIA|JUN|CASP8|TNF|MAPK10|NFKB1|RELA|MAPK9|CSNK2A1|PPP1CC	4.89E-06	1.53E-05	Endocrine system	CASP3, CSNK2A1, FOS, JUN, NFKB1, RELA	
PCA	Herpes simplex infection	CASP3|NFKBIB|CASP8|TNF|NFKB1|RELA	0.004116961	0.011888303	Immune system	CASP3, NFKB1, RELA	
SB	Herpes simplex infection	CASP3|FOS|JUN|CYCS|TNF|MAPK10|CASP8|MAPK8|MAPK9	1.72E-05	0.00014092	Signal transduction	CASP3, CYCS, FOS, JUN	
DA	HIF-1 signaling pathway	AKT1|RELA|calcium ions|MAPK3	0.002342595	0.023155651	Lipid metabolism	AKT1, RELA	

FA	HIF-1 signaling pathway	MAPK3|IFNG|PIK3CB|calcium ions|IL6R|AKT1|MAPK1|RELA	4.79E-07	1.92E-05	Cancers	AKT1, RELA		
PCA	HIF-1 signaling pathway	MAPK3|PIK3CA|PIK3CB|calcium ions|PLCG2|AKT1|AKT3|NFKB1|MAPK1|RELA	1.23E-07	1.17E-06	Excretory system	AKT1, NFKB1, RELA		
SA	HIF-1 signaling pathway	calcium ions|IFNG|EGFR|IL6R|MAPK3|MAPK1	0.000199565	0.002564406	Endocrine and metabolic diseases			
SB	HIF-1 signaling pathway	MAPK3|PRKCB|TLR4|calcium ions|AKT1	0.004824076	0.015119361	Immune diseases	AKT1		
FA	HTLV-I infection	TP53|calcium ions|PIK3CB|NFKBIA|AKT1|TNF|ICAM1|RELA	0.000365399	0.002041466	Nervous system	AKT1, RELA, TP53	ICAM1	
PCA	HTLV-I infection	TP53|calcium ions|PIK3CA|PIK3CB|AKT1|AKT3|ICAM1|NFKB1|RELA|CDKN1A|TNF	8.52E-05	0.000384286	Cancers	AKT1, NFKB1, RELA, TP53	ICAM1	
SB	HTLV-I infection	TGFB1|TGFBR2|TGFBR1|TP53|calcium ions|JUN|AKT1|TNF|ICAM1|MAPK8	0.000362114	0.001723394	Metabolism of other amino acids	AKT1, JUN, TGFBR1, TGFBR2, TP53	ICAM1	
CA	Huntington's disease	CASP3|glutamic acid|calcium ions	0.004865744	0.037893825	Xenobiotics biodegradation and metabolism	CASP3		
EA	Huntington's disease	PLCB1|CASP3|calcium ions|CASP8|CASP9|glutamic acid|APAF1	1.87E-06	6.10E-06	Nervous system	CASP3		APAF1
PCA	Huntington's disease	CASP8|CASP3|glutamic acid|calcium ions	0.000188603	0.00074811	Infectious diseases	CASP3		
SA	Huntington's disease	CASP3|glutamic acid|calcium ions	0.00144959	0.011289232	Carbohydrate metabolism	CASP3		
SB	Huntington's disease	glutamic acid|CASP3|calcium ions|CASP8|CASP9|CYCS	2.84E-07	4.04E-06	Immune system	CASP3, CYCS		
TIIA	Huntington's disease	APAF1|CASP9|CASP3|glutamic acid|calcium ions	5.80E-06	0.000124206	Cardiovascular diseases	CASP3		APAF1
EA	Inflammatory bowel disease (IBD)	TGFB1|STAT3|STAT1|STAT6|JUN|SMAD3|TNF|NFKB1|RELA	3.05E-05	8.81E-05	Endocrine system	JUN, NFKB1, RELA, STAT3		

SB	Inflammatory bowel disease (IBD)	TGFB1|TNF|JUN|TLR4	0.006425117	0.01920064	Immune system	JUN
PCA	Inflammatory mediator regulation of TRP channels	calcium ions|CALML3|PIK3CA|PIK3CB|PLA2G4F|PLCG2|IL1B|PLA2G4A|arachidonic acid	0.000189211	0.00074811	Substance dependence	
SA	Inflammatory mediator regulation of TRP channels	SRC|calcium ions|CALML3|IL1B|PLA2G4F|arachidonic acid	0.004740022	0.026020593	Signal transduction	
SB	Inflammatory mediator regulation of TRP channels	PLA2G4A|calcium ions|CALML3|PRKCB|PRKCD|PLA2G4F|MAPK10|IL1B|MAPK9|MAPK8|arachidonic acid	4.64E-06	4.41E-05	Infectious diseases	
FA	Influenza A	MAPK3|IFNG|PIK3CB|NFKBIA|AKT1|MAPK1|RELA	2.95E-05	0.000313594	Signal transduction	AKT1, RELA
PCA	Influenza A	MAPK3|NFKBIB|PIK3CA|PIK3CB|AKT1|AKT3|NFKB1|MAPK1|RELA|IL1B	1.07E-06	7.46E-06	Nervous system	AKT1, NFKB1, RELA
SB	Influenza A	MAPK3|JUN|CYCS|CASP9|AKT1|MAPK10|TLR4|IL1B|MAPK9|MAPK8	9.68E-07	1.04E-05	Endocrine and metabolic diseases	AKT1, CYCS, JUN
CA	Insulin resistance	PCK2|NOS3|G6PC3|PTPA|PYGB|TNF|MAPK10|PIK3R1|PYGL|glucose	1.26E-05	0.000294162	Cancers	
CT	Insulin resistance	PIK3CB|NOS3|PYGL|PYGB|glucose	0.003258569	0.049261889	Signal transduction	
DA	Insulin resistance	AKT1|RELA|NOS3|glucose	0.007715529	0.052181339	Carbohydrate metabolism	AKT1, RELA
FA	Insulin resistance	NOS3|PTPA|PIK3CB|NFKBIA|AKT1|PYGB|TNF|RELA|glucose	5.61E-07	1.92E-05	Global and overview maps	AKT1, RELA
PCA	Insulin resistance	NOS3|PPP1R3B|PTPA|PIK3CA|PIK3CB|AKT1|PYGB|AKT3|NFKB1|RELA|PYGL|PYGM|TNF|glucose	2.78E-10	1.75E-08	Endocrine system	AKT1, NFKB1, RELA
SA	Insulin resistance	PCK1|PTPA|NOS3|PTPRF|PYGB|TNF|PYGL|glucose	2.25E-05	0.000576342	Infectious diseases	

SB	Insulin resistance	PCK1|PPP1R3A|PTPA|PRKCB|PRKCD|NOS3|AKT1|PYGB|TNF|MAPK10|MAPK8|MAPK9|PYGL|glucose	2.39E-10	1.53E-08	Infectious diseases	AKT1	
TI	Insulin resistance	PCK1|PTPA|PYGB|NOS3|PYGL|TNF|glucose	0.000134094	0.003446207	Immune system		
TIIA	Insulin resistance	PCK1|NOS3|G6PC2|PTPA|PYGB|TNF|PPARA|PYGL|glucose	1.15E-05	0.000211499	Cellular community - eukaryotes		
PCA	Insulin signaling pathway	PPP1R3B|MAPK3|GCK|PIK3CA|PIK3CB|CALML3|AKT1|PYGB|AKT3|MAPK1|PYGL|PYGM	1.39E-06	9.15E-06	Neurodegenerative diseases	AKT1	
SA	Insulin signaling pathway	PCK1|SHC1|PTPRF|CALML3|MAPK3|PYGB|MAPK1|PYGL	0.00023939	0.002857023	Infectious diseases		
SB	Insulin signaling pathway	PCK1|PPP1R3A|MAPK3|CALML3|AKT1|PYGB|MAPK10|MAPK8|MAPK9|PYGL	5.03E-05	0.0003066	Signal transduction	AKT1	
EA	Jak-STAT signaling pathway	BCL2L1|STAT6|EP300|STAT3|STAT1|CCND1|PIK3CA|PIK3CB|PIK3CG|AKT1|PIK3R5|PIK3R1|MYC|IL6R|IRF9	0.000288238	0.000733437	Carbohydrate metabolism	AKT1, BCL2L1, STAT3	
CA	Legionellosis	CXCL8|CXCL2|CASP3|TNF	0.00419974	0.0348172	Global and overview maps	CASP3	
EA	Legionellosis	CASP3|CXCL8|NFKBIA|CD14|CASP9|HSPA1A|TNF|NFKB1|RELA|IL1B|APAF1	1.79E-08	8.09E-08	Amino acid metabolism	CASP3, CD14, NFKB1, RELA	APAF1
FA	Legionellosis	RELA|NFKBIA|TNF	0.005737087	0.020399688	Global and overview maps	RELA	
PCA	Legionellosis	NFKB1|IL1B|TNF|RELA|CASP3	0.000225869	0.000879521	Nervous system	CASP3, NFKB1, RELA	
SA	Legionellosis	IL1B|TNF|CASP3	0.009217462	0.03948146	Immune system	CASP3	
SB	Legionellosis	CASP3|CYCS|CASP9|TNF|TLR4|IL1B	1.65E-05	0.00014092	Endocrine system	CASP3, CYCS	
TIIA	Legionellosis	CXCL8|TNF|CASP3|APAF1|CASP9	0.000154747	0.001807723	Endocrine system	CASP3	APAF1
DA	Leishmaniasis	PTGS2|RELA|calcium ions|MAPK3	0.00031949	0.005473923	Amino acid metabolism	RELA	

EA	Leishmaniasis	PTGS2|PRKCB|STAT1|TGFB1|JUN|NFKBIA|FCGR3A|calcium ions|FCGR2A|MAPK14|MAPK3|MAPK1|MAPK11|FCGR1A|NFKB1|RELA	1.71E-13	1.47E-12	Lipid metabolism	JUN, NFKB1, RELA	
FA	Leishmaniasis	PTGS2|calcium ions|NFKBIA|MAPK3|MAPK1|RELA	3.52E-06	6.02E-05	Global and overview maps	RELA	
PCA	Leishmaniasis	PTGS2|NOS2|calcium ions|NFKBIB|FCGR2A|MAPK3|MAPK1|FCGR1A|NFKB1|RELA	5.70E-10	2.09E-08	Nervous system	NFKB1, RELA	
SA	Leishmaniasis	PTGS2|NOS2|MAPK1|calcium ions|MAPK3	0.000137794	0.001967385	Digestive system		
SB	Leishmaniasis	PTGS2|NOS2|TGFB1|PRKCB|JUN|calcium ions|MAPK3|TLR4	1.99E-07	3.40E-06	Global and overview maps	JUN	
TIIA	Leishmaniasis	PTGS2|MAPK1|calcium ions|MAPK3	0.003092316	0.018481983	Signal transduction		
EA	Leukocyte transendothelial migration	PTK2|ICAM1|PIK3CA|PIK3CB|PRKCB|PIK3R5|PIK3CG|CTNNB1|PLCG2|PTK2B|VCAM1|PIK3R1|ACTB|GNAI3	1.48E-06	4.87E-06	Endocrine system		ICAM1, VCAM1
CA	Linoleic acid metabolism	linoleic acid|CYP1A2|CYP2E1|PLA2G2A|ALOX15|PLA2G1B|PLA2G4A|PLA2G4F	1.03E-07	5.30E-06	Cancers	CYP2E1	PLA2G2A
CT	Linoleic acid metabolism	linoleic acid|CYP1A2|CYP2E1|PLA2G2A|PLA2G1B|CYP3A4|PLA2G4F	2.85E-08	2.44E-06	Metabolism of other amino acids	CYP2E1	PLA2G2A
DA	Linoleic acid metabolism	linoleic acid|CYP2E1|PLA2G2A|PLA2G1B|PLA2G4A|PLA2G4F	1.75E-07	2.24E-05	Infectious diseases	CYP2E1	PLA2G2A
EA	Linoleic acid metabolism	CYP2J2|linoleic acid|CYP1A2|PLA2G16|CYP2E1|PLA2G4F|PLA2G6|ALOX15|PLA2G1B|PLA2G4B|CYP3A4|PLA2G4A|PLA2G2A	2.12E-11	1.18E-10	Signal transduction	CYP2E1	PLA2G2A
FA	Linoleic acid metabolism	linoleic acid|CYP2E1|PLA2G2A|PLA2G1B|PLA2G4A|PLA2G4F	7.97E-07	2.05E-05	Endocrine system	CYP2E1	PLA2G2A
PCA	Linoleic acid metabolism	linoleic acid|CYP1A2|CYP2E1|PLA2G2A|PLA2G1B|PLA2G4A|PLA2G4F	5.96E-07	4.38E-06	Signal transduction	CYP2E1	PLA2G2A
SA	Linoleic acid metabolism	linoleic acid|CYP1A2|CYP2E1|PLA2G2A|PLA2G1B|PLA2G4F	2.22E-06	0.000190085	Signal transduction	CYP2E1	PLA2G2A
SB	Linoleic acid metabolism	linoleic acid|CYP2E1|PLA2G2A|ALOX15|PLA2G1B|PLA2G4A|PLA2G4F	5.52E-07	6.17E-06	Cancers	CYP2E1	PLA2G2A

TI	Linoleic acid metabolism	linoleic acid|CYP1A2|CYP2E1|PLA2G1B|CYP3A4|PLA2G4F	1.71E-06	0.000109835	Cancers	CYP2E1	
TIIA	Linoleic acid metabolism	CYP2C9|linoleic acid|CYP2E1|PLA2G2A|PLA2G1B|PLA2G4A|PLA2G4F	3.43E-07	1.47E-05	Signal transduction	CYP2E1	PLA2G2A
CA	Long-term depression	PLA2G4A|GNAI2|PLA2G4F|arachidonic acid|calcium ions	0.006443261	0.047108542	Cancers		
DA	Long-term depression	MAPK3|PLA2G4A|PLA2G4F|arachidonic acid|calcium ions	0.000139602	0.002989809	Signal transduction		
EA	Long-term depression	PLCB1|IGF1R|IGF1|calcium ions|HRAS|PRKCB|PLA2G4F|MAPK3|MAPK1|GNA12|PLA2G4B|PLA2G4A|GNAI3|arachidonic acid	2.29E-08	1.01E-07	Endocrine system		
FA	Long-term depression	calcium ions|MAPK3|MAPK1|PLA2G4A|PLA2G4F|arachidonic acid	4.22E-05	0.000339051	Immune system		
PCA	Long-term depression	calcium ions|MAPK3|MAPK1|PLA2G4A|PLA2G4F|arachidonic acid	0.000473485	0.001738368	Lipid metabolism		
SA	Long-term depression	MAPK3|MAPK1|PLA2G4F|arachidonic acid|calcium ions	0.001004416	0.008066713	Signal transduction		
SB	Long-term depression	calcium ions|PRKCB|MAPK3|PLA2G4A|PLA2G4F|arachidonic acid	0.000446106	0.00204731	Immune system		
TIIA	Long-term depression	calcium ions|MAPK3|MAPK1|PLA2G4A|PLA2G4F|arachidonic acid	0.000306754	0.003153433	Circulatory system		
EA	Long-term potentiation	PLCB1|PPP1CC|calcium ions|HRAS|PRKCB|glutamic acid|MAPK3|MAPK1|EP300	0.000681357	0.001667702	Infectious diseases		
FA	Long-term potentiation	MAPK3|MAPK1|glutamic acid|calcium ions	0.005794464	0.020399688	Endocrine system		
PCA	Long-term potentiation	CALML3|MAPK3|MAPK1|glutamic acid|calcium ions	0.005056411	0.013973094	Nervous system		
SA	Long-term potentiation	CALML3|MAPK3|MAPK1|glutamic acid|calcium ions	0.001606256	0.012141402	Immune system		
SB	Long-term potentiation	CALML3|MAPK3|PRKCB|glutamic acid|calcium ions	0.004824076	0.015119361	Infectious diseases		
DA	MAPK signaling pathway	PLA2G4A|TP53|MAPK3|calcium ions|AKT1|RELA|PLA2G4F	0.00365825	0.032419663	Infectious diseases	AKT1, RELA, TP53	
FA	MAPK signaling pathway	TP53|MAPK3|calcium ions|PLA2G4F|AKT1|MAPK1|RELA|PLA2G4A|TNF	0.000993411	0.004479065	Neurodegenerative diseases	AKT1, RELA, TP53	

PCA	MAPK signaling pathway	PLA2G4A|TP53|MAPK3|calcium ions|PLA2G4F|AKT1|AKT3|NFKB1|MAPK1|RELA|IL1B|TNF|CASP3	0.000154785	0.000641607	Cancers	AKT1, CASP3, NFKB1, RELA, TP53
SA	MAPK signaling pathway	TP53|CASP3|calcium ions|EGFR|JUND|MAPK3|MAPK1|IL1B|PLA2G4F|TNF	0.000891884	0.007640474	Cancers	CASP3, JUN, TP53
SB	MAPK signaling pathway	TGFB1|TGFBR2|TGFBR1|PRKCB|TP53|CASP3|calcium ions|MAPK8|JUN|PLA2G4F|AKT1|TNF|MAPK10|PLA2G4A|IL1B|MAPK9|MAPK3	2.74E-07	4.04E-06	Neurodegenerative diseases	AKT1, CASP3, JUN, TGFBR1, TGFBR2, TP53
TIIA	MAPK signaling pathway	TP53|CASP3|calcium ions|EGFR|PLA2G4F|MAPK3|TNF|MAPK1|MYC|PLA2G4A	0.003612784	0.021101943	Signal transduction	CASP3, TP53
EA	Measles	STAT3|TP53|STAT1|CCND1|PIK3CA|PIK3CB|NFKBIA|PIK3CG|AKT1|PIK3R5|PIK3R1|NFKB1|RELA|GSK3B|IRF9	8.98E-07	3.12E-06	Global and overview maps	AKT1, GSK3B, NFKB1, RELA, STAT3, TP53
FA	Measles	TP53|IFNG|PIK3CB|NFKBIA|AKT1|RELA	0.000428559	0.002247746	Endocrine system	AKT1, RELA, TP53
PCA	Measles	TP53|NFKBIB|PIK3CA|PIK3CB|AKT1|AKT3|NFKB1|RELA	0.000130467	0.000549673	Infectious diseases	AKT1, NFKB1, RELA, TP53
EA	Melanogenesis	PLCB1|calcium ions|HRAS|PRKCB|GSK3B|CTNNB1|MAPK3|MAPK1|GNAI3	0.00491521	0.010796659	Signal transduction	GSK3B
EA	Melanoma	PDGFRB|IGF1R|IGF1|TP53|MAPK3|CCND1|PIK3CA|HRAS|EGFR|PIK3CG|AKT1|PIK3R5|CDKN1A|PIK3R1|MAPK1|PIK3CB|MET	5.32E-11	2.85E-10	Amino acid metabolism	AKT1, MET, TP53
FA	Melanoma	AKT1|PIK3CB|MAPK1|TP53|MAPK3	0.000564608	0.002845183	Excretory system	AKT1, TP53
PCA	Melanoma	TP53|MAPK3|PIK3CA|PIK3CB|AKT1|AKT3|MAPK1|CDKN1A	8.67E-06	4.84E-05	Amino acid metabolism	AKT1, TP53
SA	Melanoma	MAPK3|MAPK1|EGFR|TP53	0.008629299	0.038236721	Cancers	TP53
TIIA	Melanoma	CCND1|MAPK3|MAPK1|EGFR|TP53	0.002784277	0.017037125	Endocrine system	TP53
CA	Metabolism of xenobiotics by cytochrome P450	CYP1A1|CYP1A2|UGT1A3|GSTT2|UGT1A10|GSTM1|GSTM2|CYP2E1|trichloroethylene|GSTP1|UGT1A8|UGT1A9|UGT1A7|1,2-dibromoethane|chloroform/methanol	3.22E-08	2.07E-06	Infectious diseases	CYP2E1, MET

CT	Metabolism of xenobiotics by cytochrome P450	CYP1A1|C14787|C14802|CYP1A2|CYP2E1|CYP3A4	0.002897221	0.046536619	Circulatory system	CYP2E1	
EA	Metabolism of xenobiotics by cytochrome P450	CYP1A1|C14787|CYP1B1|GSTA4|GSTM1|CYP1A2|CYP2E1|GSTP1|BP-7,8-diol|CYP3A4|1,2-dibromoethane	0.008775854	0.018188666	Signal transduction	CYP1B1, CYP2E1	
TI	Metabolism of xenobiotics by cytochrome P450	CYP1A1|C14787|C14802|CYP1A2|CYP2E1|GSTP1|CYP3A4	0.001111273	0.021969011	Signal transduction	CYP2E1	
PCA	MicroRNAs in cancer	PTGS2|TP53|CASP3|PIK3CA|CDKN1A|PLCG2|MAPK1|VEGFA|NFKB1|BCL2	0.007150406	0.018944891	Infectious diseases	BCL2, CASP3, NFKB1, TP53	VEGFA
EA	mTOR signaling pathway	IGF1|MAPK3|PIK3CA|PIK3CB|IRS1|PIK3CG|AKT1|PIK3R5|MAPK1|PIK3R1|TNF	7.29E-07	2.64E-06	Cancers	AKT1	
FA	mTOR signaling pathway	AKT1|PIK3CB|MAPK1|TNF|MAPK3	0.000141465	0.000886742	Cancers	AKT1	
PCA	mTOR signaling pathway	MAPK3|PIK3CA|PIK3CB|AKT1|AKT3|VEGFA|MAPK1|TNF	8.88E-07	6.34E-06	Immune system	AKT1	VEGFA
EA	Natural killer cell mediated cytotoxicity	NFATC2|CASP3|calcium ions|SYK|PIK3CA|PIK3CB|FCGR3A|PIK3R5|PIK3CG|PLCG2|MAPK3|PTK2B|MAPK1|ZAP70|PIK3R1|CD247|HRAS|TNF	1.45E-07	5.65E-07	Neurodegenerative diseases	CASP3	
FA	Natural killer cell mediated cytotoxicity	calcium ions|IFNG|PIK3CB|MAPK3|MAPK1|TNF	0.001438795	0.006267294	Development		
PCA	Natural killer cell mediated cytotoxicity	CASP3|calcium ions|SYK|PIK3CA|PIK3CB|PLCG2|MAPK3|MAPK1|TNF	0.000116237	0.000515051	Lipid metabolism	CASP3	
SA	Natural killer cell mediated cytotoxicity	SHC1|calcium ions|IFNG|MAPK3|MAPK1|TNF|CASP3	0.000639285	0.005867727	Immune system	CASP3	

CT	Neomycin, kanamycin and gentamicin biosynthesis	GCK|glucose	0.002096622	0.041448606	Carbohydrate metabolism	
PCA	Neomycin, kanamycin and gentamicin biosynthesis	GCK|glucose	0.004925402	0.013759003	Lipid metabolism	
TI	Neomycin, kanamycin and gentamicin biosynthesis	GCK|glucose	0.002696744	0.043316445	Carbohydrate metabolism	
DA	Neurotrophin signaling pathway	AKT1|RELA|calcium ions|TP53|MAPK3	0.002239504	0.023022105	Lipid metabolism	AKT1, RELA, TP53
FA	Neurotrophin signaling pathway	TP53|MAPK3|PIK3CB|NFKBIA|calcium ions|AKT1|MAPK1|RELA	2.13E-05	0.000249057	Cancers	AKT1, RELA, TP53
PCA	Neurotrophin signaling pathway	TP53|calcium ions|NFKBIB|PIK3CA|PIK3CB|CALML3|PLCG2|AKT1|AKT3|NFKB1|MAPK1|RELA|MAPK3	2.60E-08	3.34E-07	Folding, sorting and degradation	AKT1, NFKB1, RELA, TP53
SA	Neurotrophin signaling pathway	TP53|SHC1|calcium ions|CALML3|MAPK3|MAPK1	0.002812461	0.018494365	Endocrine system	TP53
SB	Neurotrophin signaling pathway	TP53|calcium ions|CALML3|JUN|PRKCD|AKT1|MAPK10|MAPK8|MAPK9|MAPK3	1.19E-05	0.000105203	Cancers	AKT1, JUN, TP53
EA	NF-kappa B signaling pathway	PRKCB|SYK|NFKBIA|CD14|TNF|ZAP70|NFKB1|NFKB2|CSNK2A1|IL1B|IL1R1	8.11E-08	3.31E-07	Immune system	CD14, CSNK2A1, NFKB1, NFKB2
PCA	NF-kappa B signaling pathway	SYK|NFKB1|IL1B|TNF	0.003780486	0.011167643	Infectious diseases	NFKB1
SB	NF-kappa B signaling pathway	IL1B|PRKCB|TLR4|TNF	0.003632857	0.01212525	Endocrine and metabolic diseases	

EA	NOD-like receptor signaling pathway	NFKBIA|CASP8|MAPK14|MAPK10|MAPK11|NFKB1|RELA|MAPK9	5.24E-05	0.000146398	Cancers	NFKB1, RELA
PCA	NOD-like receptor signaling pathway	NFKBIB|NFKB1|RELA|CASP8	0.003452821	0.010439707	Endocrine system	NFKB1, RELA
SB	NOD-like receptor signaling pathway	MAPK8|MAPK9|MAPK10|CASP8	0.003317537	0.011521716	Immune system	
FA	Non-alcoholic fatty liver disease (NAFLD)	PIK3CB|IL6R|AKT1|TNF|RELA|glucose	8.93E-05	0.000620009	Endocrine system	AKT1, RELA
PCA	Non-alcoholic fatty liver disease (NAFLD)	CASP3|PIK3CA|PIK3CB|CASP8|AKT1|AKT3|NFKB1|RELA|IL1B|TNF|glucose	1.32E-08	1.89E-07	Cancers	AKT1, CASP3, NFKB1, RELA
SA	Non-alcoholic fatty liver disease (NAFLD)	IL1B|IL6R|CASP3|TNF|glucose	0.001818659	0.012983203	Amino acid metabolism	CASP3
SB	Non-alcoholic fatty liver disease (NAFLD)	CASP3|JUN|CYCS|AKT1|TNF|MAPK10|CASP8|IL1B|MAPK9|MAPK8|glucose	1.17E-08	2.74E-07	Global and overview maps	AKT1, CASP3, CYCS, JUN
TIIA	Non-alcoholic fatty liver disease (NAFLD)	TNF|CASP3|RXRA|PPARA|glucose	0.004045326	0.021217319	Signal transduction	CASP3
DA	Non-small cell lung cancer	AKT1|calcium ions|MAPK3	0.006049651	0.044231838	Signal transduction	AKT1
EA	Non-small cell lung cancer	ERBB2|MAPK3|CCND1|PIK3CA|HRAS|PRKCB|EGFR|calcium ions|PIK3CG|FOXO3|AKT1|PIK3R5|MAPK1|PIK3R1|PLCG2|CASP9|PIK3CB	7.96E-14	8.19E-13	Infectious diseases	AKT1

FA	Non-small cell lung cancer	AKT1|PIK3CB|MAPK1|calcium ions|MAPK3	0.000104849	0.00069093	Cellular community - eukaryotes	AKT1
PCA	Non-small cell lung cancer	MAPK3|PIK3CA|PIK3CB|calcium ions|PLCG2|AKT1|AKT3|MAPK1	5.41E-07	4.09E-06	Nervous system	AKT1
SA	Non-small cell lung cancer	MAPK3|MAPK1|EGFR|calcium ions	0.002408287	0.016727829	Cancers	
SB	Non-small cell lung cancer	CASP9|AKT1|PRKCB|calcium ions|MAPK3	0.000762186	0.003264696	Cellular community - eukaryotes	AKT1
TIIA	Non-small cell lung cancer	calcium ions|CCND1|EGFR|CASP9|FOXO3|MAPK3|MAPK1	4.28E-06	0.000100054	Carbohydrate metabolism	
CA	Olfactory transduction	calcium ions	0.006598862	0.047108542	Cancers	
EA	Olfactory transduction	calcium ions	1.36E-05	4.07E-05	Neurodegenerative diseases	
DA	Osteoclast differentiation	AKT1|RELA|calcium ions|MAPK3	0.0097778	0.062822362	Immune system	AKT1, RELA
FA	Osteoclast differentiation	MAPK3|IFNG|PIK3CB|NFKBIA|calcium ions|AKT1|MAPK1|RELA|TNF	1.01E-06	2.16E-05	Infectious diseases	AKT1, RELA
PCA	Osteoclast differentiation	MAPK3|calcium ions|SYK|PIK3CA|PIK3CB|FCGR2A|PLCG2|AKT1|AKT3|FCGR1A|NFKB1|MAPK1|RELA|IL1B|TNF	5.95E-11	5.09E-09	Metabolism of other amino acids	AKT1, NFKB1, RELA
SA	Osteoclast differentiation	calcium ions|IFNG|JUND|MAPK3|MAPK1|IL1B|TNF	0.000271246	0.003030881	Endocrine and metabolic diseases	JUN
SB	Osteoclast differentiation	MAPK8|TGFBR2|NCF1|TGFB1|FOS|calcium ions|JUN|TGFBR1|AKT1|TNF|MAPK10|IL1B|MAPK9|MAPK3	6.08E-10	2.23E-08	Infectious diseases	AKT1, FOS, JUN, TGFBR1, TGFBR2
EA	Ovarian steroidogenesis	IGF1R|IGF1|CYP17A1|INSR|PLA2G4B|PLA2G4A|PLA2G4F	0.000438053	0.001093007	Nervous system	

CA	Oxytocin signaling pathway	PTGS2|NOS3|calcium ions|EGFR|PIK3R1|PLA2G4A|GNAI2|PLA2G4F|arachidonic acid	0.003527528	0.033576839	Infectious diseases		
CT	Oxytocin signaling pathway	PTGS2|NOS3|calcium ions|PIK3CB|PLA2G4F|arachidonic acid	0.006673497	0.081670891	Infectious diseases		
DA	Oxytocin signaling pathway	PTGS2|NOS3|calcium ions|MAPK3|PLA2G4A|PLA2G4F|arachidonic acid	0.000302132	0.005473923	Immune system		
FA	Oxytocin signaling pathway	PTGS2|NOS3|calcium ions|PIK3CB|MAPK3|MAPK1|PLA2G4A|PLA2G4F|arachidonic acid	4.03E-05	0.00033401	Neurodegenerative diseases		
PCA	Oxytocin signaling pathway	PTGS2|NOS3|calcium ions|CALML3|PIK3CA|PIK3CB|PLA2G4F|MAPK3|MAPK1|PLA2G4A|CDKN1A|arachidonic acid	9.66E-06	5.17E-05	Metabolism of other amino acids		
SA	Oxytocin signaling pathway	SRC|NOS3|PTGS2|CALML3|calcium ions|EGFR|MAPK3|MAPK1|PLA2G4F|arachidonic acid	2.69E-05	0.000576342	Immune system		
SB	Oxytocin signaling pathway	PTGS2|NOS3|FOS|CALML3|calcium ions|JUN|PRKCB|MAPK3|PLA2G4A|PLA2G4F|arachidonic acid	4.74E-05	0.000304797	Nervous system	FOS, JUN	
TIIA	Oxytocin signaling pathway	PTGS2|NOS3|calcium ions|CCND1|EGFR|MAPK3|MAPK1|PLA2G4A|PLA2G4F|arachidonic acid	0.000132288	0.001699907	Immune system		
EA	p53 signaling pathway	IGF1|TP53|CASP3|CDKN1A|CASP8|CASP9|APAF1	0.00291756	0.006577307	Nervous system	CASP3, TP53	APAF1
SB	p53 signaling pathway	CASP8|TP53|CASP3|CYCS|CASP9	0.001699825	0.006720845	Cancers	CASP3, CYCS, TP53	
TIIA	p53 signaling pathway	TP53|CASP3|APAF1|CASP9	0.008698353	0.038542702	Signal transduction	CASP3, TP53	APAF1
FA	Pancreatic cancer	AKT1|PIK3CB|MAPK1|RELA|MAPK3	0.000393373	0.002106184	Cancers	AKT1, RELA	
PCA	Pancreatic cancer	MAPK3|PIK3CA|PIK3CB|AKT1|AKT3|VEGFA|NFKB1|MAPK1|RELA	4.20E-07	3.60E-06	Amino acid metabolism	AKT1, NFKB1, RELA	VEGFA
SA	Pancreatic cancer	BCL2L1|MAPK3|MAPK1|EGFR	0.006571647	0.033778264	Signal transduction	BCL2L1	

SB	Pancreatic cancer	BCL2L1|TGFBR2|TGFBR1|MAPK3|CASP9|AKT1|MAPK10|MAPK8|MAPK9	3.82E-07	4.90E-06	Cancers	AKT1, BCL2L1, TGFBR1, TGFBR2	
TIIA	Pancreatic cancer	CCND1|MAPK3|MAPK1|EGFR|CASP9	0.001974632	0.013354747	Signal transduction		
CA	Pathways in cancer	PTGS2|TP53|PIK3R1|calcium ions|CXCL8|EGFR|HIF1A|AR|MAPK10|estradiol|cholesterol|SHH|DHEA|CASP3	0.000378106	0.005398508	Signal transduction	CASP3, TP53	
DA	Pathways in cancer	PTGS2|TP53|AKT1|calcium ions|MAPK3|cholesterol|RELA	0.004817894	0.0399419	Carbohydrate metabolism	AKT1, RELA, TP53	
FA	Pathways in cancer	PTGS2|TP53|AKT1|PIK3CB|NFKBIA|calcium ions|MAPK3|MAPK1|cholesterol|SHH|RELA	6.65E-05	0.000488338	Cancers	AKT1, RELA, TP53	
SA	Pathways in cancer	BCL2L1|PTGS2|NOS2|TP53|CASP3|calcium ions|EGFR|MAPK3|MAPK1|cholesterol|SHH	0.000326644	0.003457907	Cell growth and death	BCL2L1, CASP3, TP53	
TIIA	Pathways in cancer	PTGS2|TP53|CASP3|calcium ions|CCND1|CXCL8|PPARG|EGFR|RXRA|RXRB|CASP9|MAPK3|MAPK1|cholesterol|SHH|MYC	1.08E-06	3.70E-05	Signal transduction	CASP3, RXRB, TP53	
CA	Pentose and glucuronate interconversions	UGT1A7|UGT1A8|UGT1A9|UGT1A10|UGT1A3	0.001828938	0.01958488	Cancers		
EA	Pertussis	IRF1|RELA|FOS|JUN|MAPK14|MAPK3|MAPK1|MAPK10|MAPK11|NFKB1|IL1B|MAPK9|GNAI3	7.79E-11	4.08E-10	Infectious diseases	FOS, JUN, NFKB1, RELA	
FA	Pertussis	MAPK3|RELA|MAPK1	0.005737087	0.020399688	Infectious diseases	RELA	
PCA	Pertussis	NOS2|MAPK3|MAPK1|NFKB1|RELA|IL1B	1.75E-05	9.02E-05	Lipid metabolism	NFKB1, RELA	
SA	Pertussis	MAPK3|IL1B|MAPK1|NOS2	0.00087934	0.007640474	Endocrine system		
SB	Pertussis	NOS2|FOS|JUN|MAPK3|MAPK10|TLR4|IL1B|MAPK9|MAPK8	2.47E-09	7.05E-08	Endocrine system	FOS, JUN	
FA	PI3K-Akt signaling pathway	NOS3|TP53|MAPK3|PIK3CB|AKT1|MAPK1|RELA	0.002865128	0.011156635	Cancers	AKT1, RELA, TP53	
PCA	PI3K-Akt signaling pathway	NOS3|TP53|MAPK3|SYK|PIK3CA|PIK3CB|AKT1|AKT3|VEGFA|NFKB1|MAPK1|RELA|CDKN1A|BCL2	1.13E-06	7.65E-06	Circulatory system	AKT1, BCL2, NFKB1, RELA, TP53	VEGFA

SA	PI3K-Akt signaling pathway	BCL2L1|PCK1|TP53|EGFR|MAPK3|NOS3|MAPK1	0.007454817	0.03614883	Endocrine system	BCL2L1, TP53
SB	PI3K-Akt signaling pathway	BCL2L1|PCK1|TP53|MAPK3|CASP9|AKT1|NOS3|TLR4|BCL2	0.002506189	0.009070988	Signal transduction	AKT1, BCL2, BCL2L1, TP53
TIIA	PI3K-Akt signaling pathway	PCK1|TP53|CCND1|EGFR|RXRA|G6PC2|FOXO3|MAPK3|NOS3|MAPK1|MYC|CASP9	1.49E-05	0.00025482	Immune diseases	TP53
CA	Platelet activation	PTGS1|NOS3|calcium ions|PIK3R1|PLA2G4A|GNAI2|PLA2G4F|arachidonic acid	0.003284956	0.032470527	Signal transduction	
CT	Platelet activation	PTGS1|NOS3|calcium ions|PIK3CB|PLA2G4F|arachidonic acid	0.002324113	0.042664075	Infectious diseases	
DA	Platelet activation	PTGS1|NOS3|MAPK3|calcium ions|AKT1|PLA2G4A|PLA2G4F|arachidonic acid	8.64E-06	0.000317037	Carbohydrate metabolism	AKT1
FA	Platelet activation	PTGS1|NOS3|MAPK3|PIK3CB|calcium ions|AKT1|MAPK1|PLA2G4A|PLA2G4F|arachidonic acid	7.92E-07	2.05E-05	Endocrine and metabolic diseases	AKT1
PCA	Platelet activation	PTGS1|NOS3|calcium ions|SYK|PIK3CA|PIK3CB|FCGR2A|PLCG2|AKT1|AKT3|MAPK1|PLA2G4A|PLA2G4F|MAPK3|arachidonic acid	1.76E-09	5.03E-08	Biosynthesis of other secondary metabolites	AKT1
SA	Platelet activation	SRC|PTGS1|NOS3|calcium ions|MAPK3|MAPK1|PLA2G4F|arachidonic acid	0.00019541	0.002564406	Cardiovascular diseases	
SB	Platelet activation	PTGS1|NOS3|MAPK3|calcium ions|AKT1|PLA2G4A|PLA2G4F|arachidonic acid	0.00108672	0.004504629	Immune system	AKT1
TIIA	Platelet activation	PTGS1|NOS3|calcium ions|MAPK3|MAPK1|PLA2G4A|PLA2G4F|arachidonic acid	0.000686868	0.00551641	Endocrine system	
CA	Porphyrin and chlorophyll metabolism	UGT1A10|glycine|ALAS2|UGT1A8|UGT1A9|UGT1A7|UGT1A3	0.000147588	0.002288121	Cell growth and death	
TIIA	PPAR signaling pathway	PPARG|RXRA|RXRB|PPARA	8.28E-06	0.000163611	Carbohydrate metabolism	RXRB

CA	Primary bile acid biosynthesis	CYP27A1|CYP7A1|CYP46A1|glycine|cholesterol	0.006027227	0.045558745	Cancers	
SA	Primary bile acid biosynthesis	CYP27A1|CYP46A1|glycine|cholesterol	0.006953815	0.035041772	Immune system	
EA	Progesterone-mediated oocyte maturation	IGF1R|IGF1|AKT1|PIK3CA|PIK3CB|PIK3CG|MAPK14|MAPK3|PIK3R5|MAPK1|MAPK10|MAPK11|PIK3R1|MAPK9|GNAI3	9.14E-08	3.67E-07	Global and overview maps	AKT1
FA	Progesterone-mediated oocyte maturation	AKT1|PIK3CB|MAPK1|MAPK3	0.009598181	0.030916919	Immune system	AKT1
PCA	Progesterone-mediated oocyte maturation	MAPK3|PIK3CA|PIK3CB|AKT1|AKT3|MAPK1	0.001761933	0.005731858	Amino acid metabolism	AKT1
SB	Progesterone-mediated oocyte maturation	MAPK3|MAPK8|MAPK9|MAPK10|AKT1	0.008843328	0.02553635	Infectious diseases	AKT1
FA	Prolactin signaling pathway	AKT1|PIK3CB|MAPK1|RELA|MAPK3	0.000786672	0.003610263	Sensory system	AKT1, RELA
PCA	Prolactin signaling pathway	MAPK3|PIK3CA|PIK3CB|AKT1|AKT3|NFKB1|RELA|GCK|MAPK1	1.55E-06	9.95E-06	Signal transduction	AKT1, NFKB1, RELA
SB	Prolactin signaling pathway	MAPK3|FOS|AKT1|MAPK10|MAPK8|MAPK9	0.000841261	0.003544329	Signal transduction	AKT1, FOS
TIIA	Prolactin signaling pathway	FOXO3|MAPK3|MAPK1|CCND1|CYP17A1	0.003811222	0.021217319	Infectious diseases	
DA	Prostate cancer	AKT1|RELA|TP53|MAPK3	0.003618993	0.032419663	Lipid metabolism	AKT1, RELA, TP53

FA	Prostate cancer	TP53|MAPK3|PIK3CB|NFKBIA|AKT1|MAPK1|RELA	1.46E-05	0.000178747	Cancers	AKT1, RELA, TP53	
PCA	Prostate cancer	TP53|MAPK3|PIK3CA|PIK3CB|AKT1|AKT3|NFKB1|RELA|CDKN1A|MAPK1|BCL2	3.56E-08	4.16E-07	Substance dependence	AKT1, BCL2, NFKB1, RELA, TP53	
SB	Prostate cancer	AKT1|CASP9|TP53|BCL2|MAPK3	0.007983144	0.02331441	Immune diseases	AKT1, BCL2, TP53	
TIIA	Prostate cancer	TP53|CCND1|EGFR|CASP9|MAPK3|GSTP1|MAPK1	0.000146784	0.00179636	Cancers	TP53	
SB	Protein processing in endoplasmic reticulum	MAPK8|MAPK9|MAPK10|BCL2	0.007964547	0.02331441	Cancers	BCL2	
PCA	Proteoglycans in cancer	TP53|CASP3|MAPK3|PIK3CA|PIK3CB|PLCG2|AKT1|AKT3|VEGFA|MAPK1|CDKN1A	0.000442471	0.001648044	Immune system	AKT1, CASP3, TP53	VEGFA
CA	Proximal tubule bicarbonate reclamation	PCK2|oxaloacetate|glutamic acid	0.000482904	0.006531913	Nervous system		
EA	Proximal tubule bicarbonate reclamation	PCK2|PCK1|GLS|glutamic acid	0.000152099	0.000415844	Endocrine and metabolic diseases		
SA	Proximal tubule bicarbonate reclamation	PCK1|glutamic acid	0.004962036	0.02656757	Lipid metabolism		
SB	Proximal tubule bicarbonate reclamation	GLS|PCK1|glutamic acid	0.00028968	0.001404676	Carbohydrate metabolism		
TI	Proximal tubule bicarbonate reclamation	PCK1|glutamic acid	0.004553968	0.068845288	Amino acid metabolism		

TIIA	Proximal tubule bicarbonate reclamation	PCK1|glutamic acid	0.007081571	0.032499352	Xenobiotics biodegradation and metabolism			
EA	Purine metabolism	POLD1	0.00327059	0.007246048	Global and overview maps			
PCA	Rap1 signaling pathway	calcium ions|CALML3|PIK3CA|PIK3CB|AKT1|AKT3|VEGFA|MAPK1|MAPK3	0.003958127	0.011559529	Signal transduction	AKT1		VEGFA
DA	Ras signaling pathway	MAPK3|calcium ions|PLA2G4F|PLA2G2A|AKT1|PLA2G1B|RELA|PLA2G4A	0.000345912	0.005556215	Cancers	AKT1, RELA	PLA2G2A	
FA	Ras signaling pathway	MAPK3|PIK3CB|calcium ions|PLA2G4F|PLA2G2A|AKT1|MAPK1|PLA2G1B|RELA|PLA2G4A	7.92E-05	0.000565281	Infectious diseases	AKT1, RELA	PLA2G2A	
PCA	Ras signaling pathway	PLA2G4A|calcium ions|CALML3|PIK3CA|PIK3CB|PLCG2|PLA2G4F|PLA2G2A|AKT1|AKT3|VEGFA|PLA2G1B|NFKB1|MAPK1|RELA|MAPK3	3.32E-07	2.97E-06	Cancers	AKT1, NFKB1, RELA	PLA2G2A	VEGFA
SA	Ras signaling pathway	BCL2L1|SHC1|calcium ions|CALML3|EGFR|PLA2G2A|MAPK3|MAPK1|PLA2G1B|PLA2G4F	0.000343059	0.003457907	Endocrine system	BCL2L1	PLA2G2A	
SB	Ras signaling pathway	BCL2L1|PLA2G4A|calcium ions|CALML3|PLA2G4F|PLA2G2A|AKT1|MAPK10|PLA2G1B|MAPK8|MAPK9|MAPK3	0.00017356	0.000857787	Immune diseases	AKT1, BCL2L1	PLA2G2A	
EA	Regulation of actin cytoskeleton	PDGFRB|PPP1CC|SRC|PIK3CA|HRAS|EGFR|CD14|PIK3CG|MAPK3|PIK3R5|MAPK1|GNA12|PIK3R1|ACTB|PIK3CB	0.000639522	0.001580358	Endocrine system	CD14		
EA	Regulation of lipolysis in adipocytes	PLA2G16|PIK3CA|PIK3CB|IRS1|PIK3CG|AKT1|INSR|PIK3R1|PIK3R5|GNAI3|arachidonic acid	4.16E-06	1.32E-05	Cancers	AKT1		
PCA	Regulation of lipolysis in adipocytes	AKT1|PIK3CB|AKT3|arachidonic acid|PIK3CA	0.002254809	0.007243574	Nervous system	AKT1		
EA	Renal cell carcinoma	TGFB1|MAPK3|PIK3CA|PIK3CB|JUN|PIK3CG|AKT1|PIK3R5|MAPK1|PIK3R1|CRK|EP300|MET	4.35E-08	1.86E-07	Infectious diseases	AKT1, JUN, MET		
FA	Renal cell carcinoma	AKT1|PIK3CB|MAPK1|MAPK3	0.002619565	0.010686162	Cancers	AKT1		

PCA	Renal cell carcinoma	MAPK3|PIK3CA|PIK3CB|AKT1|AKT3|VEGFA|MAPK1	2.71E-05	0.000136617	Infectious diseases	AKT1		VEGFA
CA	Retinol metabolism	CYP1A1|CYP1A2|UGT1A10|UGT1A8|UGT1A9|UGT1A7|UGT1A3	0.00065431	0.008407889	Infectious diseases			
EA	Retrograde endocannabinoid signaling	PLCB1|calcium ions|PRKCB|glutamic acid|MAPK14|MAPK3|MAPK1|MAPK10|MAPK11|MAPK9|GNAI3	3.46E-05	9.77E-05	Cell growth and death			
FA	Retrograde endocannabinoid signaling	MAPK3|MAPK1|glutamic acid|calcium ions	0.006393451	0.021908225	Cancers			
SB	Retrograde endocannabinoid signaling	calcium ions|PRKCB|glutamic acid|MAPK3|MAPK10|MAPK8|MAPK9	0.000126917	0.000639565	Cancers			
EA	Rheumatoid arthritis	TEK|JUN|ICAM1|FOS	0.00231055	0.005254967	Endocrine system	FOS, JUN	ICAM1	
SB	Rheumatoid arthritis	JUN|TLR4|ICAM1|FOS	0.000113638	0.00059602	Immune system	FOS, JUN	ICAM1	
EA	RIG-I-like receptor signaling pathway	NFKBIA|MAP3K1|MAPK14|MAPK10|MAPK11|NFKB1|RELA|MAPK9	2.88E-05	8.40E-05	Lipid metabolism	NFKB1, RELA		
EA	Salmonella infection	FOS|JUN|MAPK14|MAPK3|CD14|MAPK1|MAPK10|MAPK11|NFKB1|RELA|MAPK9|ACTB	1.18E-06	3.99E-06	Infectious diseases	CD14, FOS, JUN, NFKB1, RELA		
SB	Salmonella infection	FOS|JUN|MAPK3|MAPK10|TLR4|MAPK8|MAPK9	4.89E-05	0.000306587	Cancers	FOS, JUN		
CA	Serotonergic synapse	PTGS2|PTGS1|CASP3|calcium ions|PLA2G4A|GNAI2|PLA2G4F	0.001410775	0.0158916	Infectious diseases	CASP3		
DA	Serotonergic synapse	PTGS2|PTGS1|calcium ions|MAPK3|PLA2G4A|PLA2G4F	7.43E-05	0.001909374	Metabolism of other amino acids			
EA	Serotonergic synapse	PLCB1|PTGS1|PRKCB|CASP3|PTGS2|HRAS|calcium ions|PLA2G4F|MAPK3|MAPK1|PLA2G4B|PLA2G4A|GNAI3	1.21E-05	3.70E-05	Infectious diseases	CASP3		
FA	Serotonergic synapse	PTGS2|PTGS1|calcium ions|MAPK3|MAPK1|PLA2G4A|PLA2G4F	3.41E-05	0.000324553	Immune system			

PCA	Serotonergic synapse	PTGS2|PTGS1|CASP3|calcium ions|MAPK3|MAPK1|PLA2G4A|PLA2G4F	8.29E-05	0.000380399	Metabolism of other amino acids	CASP3	
SA	Serotonergic synapse	PTGS2|PTGS1|CASP3|calcium ions|MAPK3|MAPK1|PLA2G4F	0.000104067	0.001623657	Signal transduction	CASP3	
SB	Serotonergic synapse	PTGS2|PTGS1|CASP3|calcium ions|PRKCB|MAPK3|PLA2G4A|PLA2G4F	7.66E-05	0.000427861	Cancers	CASP3	
TIIA	Serotonergic synapse	PTGS2|PTGS1|CASP3|calcium ions|MAPK3|MAPK1|PLA2G4A|PLA2G4F	4.65E-05	0.000639052	Signal transduction	CASP3	
EA	Shigellosis	CXCL8|NFKBIA|MAPK14|MAPK3|MAPK1|MAPK10|MAPK11|NFKB1|RELA|MAPK9|ACTB	8.11E-08	3.31E-07	Metabolism of other amino acids	NFKB1, RELA	
FA	Shigellosis	MAPK3|RELA|MAPK1|NFKBIA	0.000758531	0.003544407	Circulatory system	RELA	
PCA	Shigellosis	NFKBIB|MAPK3|RELA|MAPK1|NFKB1	0.00042192	0.00159461	Nervous system	NFKB1, RELA	
SB	Shigellosis	MAPK3|MAPK8|MAPK9|MAPK10	0.003632857	0.01212525	Signal transduction		
DA	Small cell lung cancer	PTGS2|RELA|AKT1	0.005695066	0.043047999	Cancers	AKT1, RELA	
EA	Small cell lung cancer	BCL2L1|PTGS2|PTK2|CCND1|PIK3CA|PIK3CB|NFKBIA|PIK3CG|AKT1|PIK3R5|PIK3R1|NFKB1|RELA|CASP9|APAF1	1.41E-11	8.23E-11	Endocrine system	AKT1, BCL2L1, NFKB1, RELA	APAF1
FA	Small cell lung cancer	PTGS2|PIK3CB|NFKBIA|RELA|AKT1	9.44E-05	0.000638651	Cell motility	AKT1, RELA	
PCA	Small cell lung cancer	PTGS2|NOS2|PIK3CA|PIK3CB|AKT1|AKT3|NFKB1|RELA	4.55E-07	3.65E-06	Signal transduction	AKT1, NFKB1, RELA	
SB	Small cell lung cancer	BCL2L1|PTGS2|NOS2|CYCS|CASP9|AKT1	6.98E-05	0.00040403	Signal transduction	AKT1, BCL2L1, CYCS	
TIIA	Small cell lung cancer	CCND1|PTGS2|APAF1|CASP9	0.004313827	0.022173069	Cancers		APAF1
DA	Sphingolipid signaling pathway	AKT1|RELA|NOS3|TP53|MAPK3	0.001216896	0.014250979	Cancers	AKT1, RELA, TP53	
FA	Sphingolipid signaling pathway	NOS3|TP53|MAPK3|PIK3CB|AKT1|MAPK1|RELA|TNF	7.72E-06	0.000104488	Cancers	AKT1, RELA, TP53	

PCA	Sphingolipid signaling pathway	NOS3|TP53|MAPK3|PIK3CA|PIK3CB|AKT1|AKT3|NFKB1|MAPK1|RELA|TNF|BCL2	4.90E-08	5.47E-07	Signal transduction	AKT1, BCL2, NFKB1, RELA, TP53
SA	Sphingolipid signaling pathway	MAPK3|MAPK1|TNF|TP53|NOS3	0.007692156	0.036608964	Nervous system	TP53
SB	Sphingolipid signaling pathway	NOS3|TP53|MAPK3|AKT1|TNF|MAPK10|MAPK8|MAPK9|BCL2	2.58E-05	0.000187254	Amino acid metabolism	AKT1, BCL2, TP53
CA	Starch and sucrose metabolism	glucose|UGT1A10|G6PC3|GANC|PYGB|UGT1A8|UGT1A9|UGT1A7|PYGL|UGT1A3	2.10E-07	8.01E-06	Infectious diseases	
CT	Starch and sucrose metabolism	GCK|SI|GANC|PYGB|sucrose|PYGL|glucose	3.24E-06	0.000119092	Infectious diseases	
EA	Starch and sucrose metabolism	SI|G6PC3|G6PC2|GANC|PYGB|PYGL|glucose	0.006196662	0.013286278	Carbohydrate metabolism	
FA	Starch and sucrose metabolism	GANC|PYGB|SI|glucose	0.003995565	0.015100887	Endocrine system	
PCA	Starch and sucrose metabolism	GCK|SI|GANC|PYGB|sucrose|PYGL|PYGM|glucose	6.11E-06	3.57E-05	Neurodegenerative diseases	
SA	Starch and sucrose metabolism	GANC|PYGB|PYGL|glucose	0.007350441	0.03614883	Nervous system	
TI	Starch and sucrose metabolism	GCK|SI|GANC|PYGB|sucrose|PYGL|glucose	7.71E-06	0.000330337	Amino acid metabolism	
TIIA	Starch and sucrose metabolism	SI|G6PC2|GANC|PYGB|PYGL|glucose	0.000306754	0.003153433	Infectious diseases	
CA	Steroid hormone biosynthesis	CYP1A1|CYP1A2|UGT1A7|estradiol|UGT1A10|DHEA|STS|estrone|CYP2E1|UGT1A8|UGT1A9|SULT2B1|cholesterol|CYP7A1|androsterone|UGT1A3	4.14E-09	3.55E-07	Amino acid metabolism	CYP2E1

CT	Steroid hormone biosynthesis	CYP1A1|CYP1A2|CYP2E1|SULT2B1|cholesterol|CYP3A4|DHEA	0.000512367	0.011970765	Infectious diseases	CYP2E1
PCA	Steroid hormone biosynthesis	CYP1A1|CYP1A2|cholesterol|CYP2E1|SULT2B1|STS|DHEA	0.006384294	0.017271194	Circulatory system	CYP2E1
TI	Steroid hormone biosynthesis	CYP1A1|CYP1A2|CYP2E1|CYP17A1|SULT2B1|cholesterol|CYP3A4|DHEA	0.000194119	0.004535335	Signal transduction	CYP2E1
DA	T cell receptor signaling pathway	AKT1|RELA|calcium ions|MAPK3	0.0061959	0.044231838	Biosynthesis of other secondary metabolites	AKT1, RELA
FA	T cell receptor signaling pathway	MAPK3|PIK3CB|NFKBIA|calcium ions|AKT1|MAPK1|RELA	3.93E-05	0.00033401	Cancers	AKT1, RELA
PCA	T cell receptor signaling pathway	calcium ions|NFKBIB|PIK3CA|PIK3CB|AKT1|AKT3|NFKB1|MAPK1|RELA|MAPK3	1.62E-06	1.02E-05	Infectious diseases	AKT1, NFKB1, RELA
SB	T cell receptor signaling pathway	FOS|calcium ions|JUN|AKT1|MAPK9|MAPK3	0.003159679	0.011123802	Endocrine system	AKT1, FOS, JUN
CA	Taurine and hypotaurine metabolism	GGT1|GAD1|GAD2	0.004153071	0.0348172	Metabolism of other amino acids	
PCA	Taurine and hypotaurine metabolism	GGT1|GAD1|GAD2	0.002622786	0.008121155	Endocrine system	
SB	Taurine and hypotaurine metabolism	GGT1|GAD1|GAD2	0.002541289	0.009070988	Amino acid metabolism	
TIIA	Taurine and hypotaurine metabolism	GGT1|GAD1|GAD2	0.002085497	0.013624943	Xenobiotics biodegradation and metabolism	
EA	Thyroid cancer	CCND1|HRAS|NTRK1|CTNNB1|MAPK3|MAPK1|MYC	3.38E-05	9.65E-05	Development	
TIIA	Thyroid cancer	PPARG|CCND1|RXRA|RXRB|MAPK3|MAPK1|MYC	1.01E-07	6.48E-06	Cancers	RXRB

DA	Thyroid hormone signaling pathway	AKT1|calcium ions|TP53|MAPK3	0.005302811	0.041297651	Immune system	AKT1, TP53
FA	Thyroid hormone signaling pathway	TP53|calcium ions|PIK3CB|AKT1|MAPK1|MAPK3	0.000268478	0.001533306	Endocrine system	AKT1, TP53
PCA	Thyroid hormone signaling pathway	TP53|calcium ions|PIK3CA|PIK3CB|PLCG2|AKT1|AKT3|MAPK1|MAPK3	9.28E-06	5.08E-05	Lipid metabolism	AKT1, TP53
SA	Thyroid hormone signaling pathway	MAPK3|MAPK1|TP53|calcium ions|SRC	0.004327369	0.024714083	Infectious diseases	TP53
SB	Thyroid hormone signaling pathway	TP53|MAPK3|PRKCB|calcium ions|CASP9|AKT1	0.002531671	0.009070988	Global and overview maps	AKT1, TP53
TIIA	Thyroid hormone signaling pathway	TP53|calcium ions|CCND1|CASP9|MAPK3|MAPK1|MYC	0.000286468	0.003153433	Cellular community - eukaryotes	TP53
FA	TNF signaling pathway	MAPK3|PIK3CB|NFKBIA|AKT1|MAPK1|RELA|TNF	7.22E-06	0.000103056	Endocrine system	AKT1, RELA
PCA	TNF signaling pathway	CASP3|MAPK3|PIK3CA|PIK3CB|CASP8|AKT1|AKT3|NFKB1|MAPK1|RELA|TNF	1.14E-08	1.89E-07	Cellular community - eukaryotes	AKT1, CASP3, NFKB1, RELA
SB	TNF signaling pathway	CASP3|MAPK3|JUN|CASP8|AKT1|TNF|MAPK10|MAPK8|MAPK9	1.41E-06	1.45E-05	Signal transduction	AKT1, CASP3, JUN
FA	Toll-like receptor signaling pathway	MAPK3|PIK3CB|NFKBIA|AKT1|MAPK1|RELA|TNF	5.88E-05	0.000444817	Immune system	AKT1, RELA

PCA	Toll-like receptor signaling pathway	MAPK3|PIK3CA|PIK3CB|CASP8|AKT1|AKT3|NFKB1|MAPK1|RELA|IL1B|TNF	3.35E-07	2.97E-06	Lipid metabolism	AKT1, NFKB1, RELA
SB	Toll-like receptor signaling pathway	FOS|MAPK8|JUN|CASP8|AKT1|TNF|MAPK10|TLR4|IL1B|MAPK9|MAPK3	2.99E-07	4.04E-06	Nervous system	AKT1, FOS, JUN
FA	Toxoplasmosis	MAPK3|IFNG|PIK3CB|NFKBIA|AKT1|MAPK1|RELA|TNF	3.27E-06	6.00E-05	Nervous system	AKT1, RELA
PCA	Toxoplasmosis	CASP3|MAPK3|NFKBIB|PIK3CA|PIK3CB|CASP8|AKT1|AKT3|NFKB1|MAPK1|RELA|TNF	1.32E-08	1.89E-07	Nervous system	AKT1, CASP3, NFKB1, RELA
SA	Toxoplasmosis	IFNG|MAPK3|MAPK1|CASP3|TNF	0.00475863	0.026020593	Infectious diseases	CASP3
SB	Toxoplasmosis	CASP3|MAPK3|CYCS|CASP9|AKT1|TNF|MAPK10|CASP8|TLR4|MAPK8|MAPK9	1.24E-07	2.28E-06	Nervous system	AKT1, CASP3, CYCS
EA	Transcriptional misregulation in cancer	SP1|CD14|RELA|FCGR1A|NFKB1	0.00036671	0.000923966	Lipid metabolism	CD14, NFKB1, RELA
PCA	Transcriptional misregulation in cancer	NFKB1|RELA|FCGR1A	0.003079195	0.00942087	Lipid metabolism	NFKB1, RELA
TIIA	Transcriptional misregulation in cancer	PPARG|RXRA|RXRB	0.00245062	0.015361205	Lipid metabolism	RXRB
FA	Tuberculosis	MAPK3|IFNG|calcium ions|AKT1|MAPK1|RELA|TNF	0.000686955	0.003269395	Cancers	AKT1, RELA
PCA	Tuberculosis	NOS2|MAPK3|calcium ions|SYK|CALML3|CASP8|AKT1|AKT3|NFKB1|MAPK1|RELA|BCL2|IL1B|TNF|CASP3	6.45E-09	1.28E-07	Global and overview maps	AKT1, BCL2, CASP3, NFKB1, RELA
SA	Tuberculosis	NOS2|CASP3|calcium ions|IFNG|CALML3|MAPK3|MAPK1|IL1B|TNF	6.21E-05	0.001139968	Endocrine and metabolic diseases	CASP3
SB	Tuberculosis	TGFB1|NOS2|CASP3|calcium ions|CALML3|MAPK8|CYCS|CASP9|AKT1|TNF|MAPK10|CASP8|TLR4|IL1B|MAPK9|MAPK3|BCL2	6.22E-11	5.33E-09	Endocrine system	AKT1, BCL2, CASP3, CYCS

EA	Type II diabetes mellitus	calcium ions|PIK3CA|PIK3CB|PRKCE|PIK3R5|IRS1|PIK3CG|MAPK3|INSR|MAPK1|MAPK10|PIK3R1|MAPK9|PRKCD|TNF	2.83E-11	1.55E-10	Metabolism of other amino acids	
FA	Type II diabetes mellitus	MAPK3|PIK3CB|MAPK1|TNF|calcium ions	0.000116129	0.000746129	Cancers	
PCA	Type II diabetes mellitus	calcium ions|PIK3CA|GCK|MAPK3|MAPK1|PIK3CB|TNF	8.47E-06	4.84E-05	Infectious diseases	
SA	Type II diabetes mellitus	MAPK3|MAPK1|TNF|calcium ions	0.002603391	0.017607142	Cancers	
SB	Type II diabetes mellitus	calcium ions|PRKCD|MAPK3|TNF|MAPK10|MAPK8|MAPK9	7.87E-06	7.22E-05	Immune system	
TIIA	Type II diabetes mellitus	MAPK3|MAPK1|TNF|calcium ions	0.005031697	0.024868193	Metabolism of other amino acids	
CT	Vascular smooth muscle contraction	PLA2G2A|calcium ions|arachidonic acid|PLA2G1B|PLA2G4F	0.008170395	0.095445064	Cancers	PLA2G2A
DA	Vascular smooth muscle contraction	calcium ions|PLA2G2A|MAPK3|PLA2G1B|PLA2G4A|PLA2G4F|arachidonic acid	4.58E-05	0.00147125	Amino acid metabolism	PLA2G2A
EA	Vascular smooth muscle contraction	PLCB1|PPP1CC|calcium ions|PRKCB|PRKCE|PRKCD|PLA2G6|MAPK3|MAPK1|GNA12|PLA2G1B|PLA2G4B|PLA2G4A|PLA2G4F|arachidonic acid|PLA2G2A	4.10E-06	1.32E-05	Excretory system	PLA2G2A
FA	Vascular smooth muscle contraction	calcium ions|PLA2G2A|MAPK3|MAPK1|PLA2G1B|PLA2G4A|PLA2G4F|arachidonic acid	3.07E-05	0.000313594	Biosynthesis of other secondary metabolites	PLA2G2A
PCA	Vascular smooth muscle contraction	calcium ions|CALML3|PLA2G2A|MAPK3|MAPK1|PLA2G1B|PLA2G4A|PLA2G4F|arachidonic acid	0.000123803	0.00053029	Nervous system	PLA2G2A

SA	Vascular smooth muscle contraction	calcium ions|CALML3|PLA2G2A|MAPK3|MAPK1|PLA2G1B|PLA2G4F|arachidonic acid	0.000107401	0.001623657	Infectious diseases		PLA2G2A	
SB	Vascular smooth muscle contraction	calcium ions|CALML3|PRKCB|PRKCD|PLA2G2A|MAPK3|PLA2G1B|PLA2G4A|PLA2G4F|arachidonic acid	1.85E-05	0.000144012	Sensory system		PLA2G2A	
TIIA	Vascular smooth muscle contraction	calcium ions|PLA2G2A|MAPK3|MAPK1|PLA2G1B|PLA2G4A|PLA2G4F|arachidonic acid	0.000386515	0.00382055	Endocrine system		PLA2G2A	
CA	VEGF signaling pathway	PTGS2|NOS3|calcium ions|PIK3R1|PLA2G4A|PLA2G4F	0.001028507	0.012586962	Signal transduction			
CT	VEGF signaling pathway	PTGS2|PIK3CB|NOS3|PLA2G4F|calcium ions	0.00042396	0.010895772	Immune system			
DA	VEGF signaling pathway	PTGS2|NOS3|AKT1|calcium ions|MAPK3|PLA2G4A|PLA2G4F	5.11E-07	4.37E-05	Cancers	AKT1		
FA	VEGF signaling pathway	PTGS2|NOS3|AKT1|PIK3CB|calcium ions|MAPK3|MAPK1|PLA2G4A|PLA2G4F	1.02E-08	2.62E-06	Sensory system	AKT1		
PCA	VEGF signaling pathway	PTGS2|NOS3|AKT1|PIK3CA|PIK3CB|calcium ions|PLCG2|MAPK3|AKT3|VEGFA|MAPK1|PLA2G4A|PLA2G4F	8.75E-12	2.25E-09	Development	AKT1		VEGFA
SA	VEGF signaling pathway	PTGS2|NOS3|SRC|calcium ions|MAPK3|MAPK1|PLA2G4F	9.31E-06	0.00039891	Endocrine and metabolic diseases			
SB	VEGF signaling pathway	PTGS2|NOS3|AKT1|PRKCB|calcium ions|CASP9|MAPK3|PLA2G4A|PLA2G4F	4.40E-07	5.20E-06	Signal transduction	AKT1		
TI	VEGF signaling pathway	PTGS2|NOS3|PLA2G4F|calcium ions	0.005956314	0.085042923	Nucleotide metabolism			
TIIA	VEGF signaling pathway	PTGS2|NOS3|calcium ions|CASP9|MAPK3|MAPK1|PLA2G4A|PLA2G4F	2.95E-06	7.59E-05	Endocrine system			

EA	Viral myocarditis	CASP8|ACTB|CASP3|ICAM1|CASP9	0.001703544	0.003980098	Lipid metabolism	CASP3	ICAM1
PCA	Viral myocarditis	CASP8|CASP3|ICAM1	0.007629	0.019804576	Signal transduction	CASP3	ICAM1
SB	Viral myocarditis	CASP8|CASP9|ICAM1|CYCS|CASP3	4.23E-05	0.000278862	Infectious diseases	CASP3, CYCS	ICAM1
TIIA	Viral myocarditis	CASP3|ICAM1|CASP9	0.006109905	0.029627274	Global and overview maps	CASP3	ICAM1
EA	Wnt signaling pathway	PLCB1|NFATC2|EP300|TP53|calcium ions|CCND1|PRKCB|JUN|GSK3B|CTNNB1|MAPK10|MYC|MAPK9|CSNK2A1	0.000193249	0.00052279	Signal transduction	CSNK2A1, GSK3B, JUN, TP53	
SB	Wnt signaling pathway	TP53|calcium ions|PRKCB|JUN|MAPK10|MAPK8|MAPK9	0.004737999	0.015119361	Signal transduction	JUN, TP53	

MB, metabolite; FDR, false discovery rate; CA, caffeic acid; CT, cryptotanshinone; DA, danshensu; EA, ellagic acid; FA, fumaric acid; PCA, protocatechuic acid; SA, salvianolic acid A; SB, salvianolic acid B; TI, tanshinone I; TIIA, tanshinone IIA.
